# Highly sensitive X-ray detectors with polymer-perovskite-embedded flexible teflon membranes

**DOI:** 10.1039/d5mh02084k

**Published:** 2026-05-28

**Authors:** Sema Sarisozen, Anne-Catherine Lehnen, Fan Hu, Gonul Ofkeli, Alexander von Reppert, Matthias Rössle, Sercan Ozen, Seydanur Kaya, Lucas Holte, Olena Maslyanchuk, Pedro B. Groszewicz, Matthias Hartlieb, Dieter Neher, Felix Lang

**Affiliations:** a Institute of Physics and Astronomy, University of Potsdam, Karl-Liebknecht-Straße 24-25 14476 Potsdam Germany felix.lang.1@uni-potsdam.de; b Institute of Chemistry, University of Potsdam, Karl-Liebknecht-Straße 24-25 Potsdam 14476 Germany; c Fraunhofer Institute for Applied Polymer Research (IAP), Geiselbergstraße 69 Potsdam Germany; d SE-ASPIN, Helmholtz-Zentrum Berlin für Materialien und Energie (HZB) 12489 Berlin Germany; e Department of Chemistry, Izmir Institute of Technology 35430 Izmir Turkey; f Helmholtz-Zentrum Berlin für Materialien und Energie GmbH, Wilhelm-Conrad-Röntgen Campus, BESSY II 12489 Berlin Germany; g Central Research Laboratory, Kastamonu University 37100 Kastamonu Turkey; h Department for Solution-Processing of Hybrid Materials and Devices, Helmholtz-Zentrum Berlin Berlin Germany; i Department of Radiation Science and Technology, Delft University of Technology Delft 2629JB Netherlands

## Abstract

Metal halide perovskites (MHPs) combine excellent optoelectronic properties with strong X-ray attenuation, offering a promising platform for high performance and adaptable radiation detectors beyond the limitations of conventional rigid semiconductors. However, state of the art performance has so far been restricted to rigid single crystal perovskite devices, while flexible film-based counterparts have significantly lagged. Here, we close this gap by embedding a polymer-perovskite composite into a mechanically robust yet flexible Teflon membrane. Through comprehensive spectroscopic analysis, we provided direct evidence for a dual-action interaction mechanism, where PMA passivates the inorganic Pb^2+^ lattice to enhance phase stability while also interacting with the organic FA^+^ cations to promote a more ordered local environment. This interaction enhances crystallinity and suppresses non-radiative recombination. As a result, our flexible detectors deliver an outstanding sensitivity of 2.3 × 10^5^ µC Gy_air_^−1^ cm^−2^ and an ultra-low detection limit of 0.09 nGy_air_ s^−1^. Importantly, this high performance is accompanied by excellent device to device reproducibility, long term stability in ambient conditions, and robust mechanical durability under bending. This work presents a comprehensive strategy for developing flexible perovskite based X-ray detectors that simultaneously achieve record sensitivity and practical reliability, enabling the development of next-generation wearable and medical imaging applications.

## Abstract 2

We report a dual-action mechanism using PMA in polymer-perovskite composites within PTFE membranes. This yields flexible X-ray detectors combining high sensitivity with excellent mechanical durability and operational stability.

## Graphical abstract

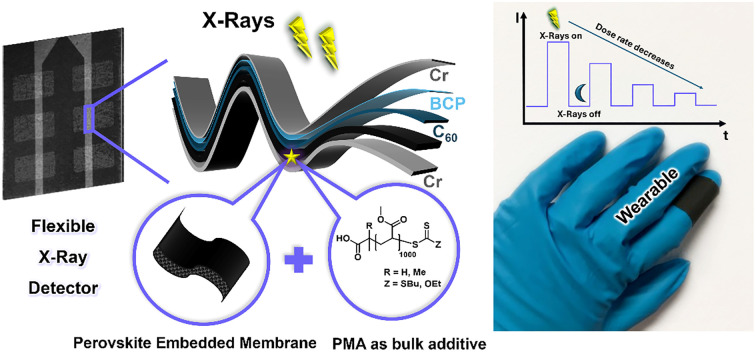

**New concepts**

This study demonstrates a development in flexible electronics by introducing a polymer-perovskite composite fabricated *via* a scalable spin coating route directly onto robust hydrophilic Teflon (PTFE) membranes. This approach allows us to investigate factors contributing to high performance in X-ray detection, specifically the role of the poly (methyl acrylate) (PMA). Our findings highlight the important role of a dual-action interaction mechanism, investigated *via* spectroscopy (FTIR, solution-state and solid-state NMR). This dual-action influence is apparent: the PMA additive appears to not only passivate the inorganic Pb^2+^ lattice (enhancing phase stability) but also interact with the organic FA^+^ cations (promoting a more ordered environment). By utilizing polymer-perovskite composite (PMA-PEM), we achieve higher sensitivity (2.3 × 10^5^ µC Gy_air_^−1^ cm^−2^) and a lower detection limit (0.09 nGy_air_ s^−1^) than a control (PEM) at 100 V. Notably, this high performance is paired with improved mechanical durability. The devices retained ∼56% sensitivity after 1000 bending fatigue cycles and ∼77.6% (corrected) sensitivity during static bending. Our work suggests that this rational molecular level design, addressing both sub-lattices, offers a promising strategy for bridging the performance-flexibility gap and developing efficient flexible detectors.

## Introduction

**Fig. 1:**
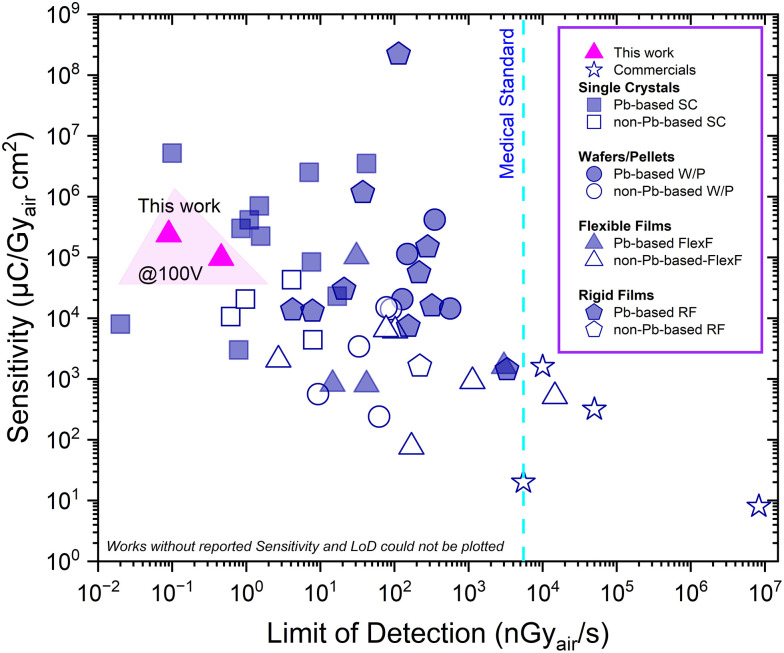
Performance comparison of Pb-based and non-Pb-based X-ray detectors, including flexible films (FlexF), rigid films (RF), solution-grown single crystals (SC), wafers/pellets (W/P), and commercial detectors. Sensitivity and detection limits were obtained from literature, with pink triangles representing this work. Our results indicate that PMA-PEM devices achieve one of the highest reported sensitivities and among the lowest detection limits compared to previously reported Pb-based and non-Pb- based flexible detectors. Additionally, both detectors exhibit a detection limit below the medical imaging standard, demonstrating their potential for real life applications. References corresponding to each data point, along with details, are provided in Table S1 of the Supporting Information.

The most commonly used materials for radiation detectors in medical diagnostics, radiation therapy, nuclear safety and environmental monitoring, are based on inorganic semiconductors like silicon,^1^ germanium,^1^ amorphous selenium (a-Se),^2^ cadmium telluride (CdTe),^3^ and cadmium zinc telluride (CZT).^3^ Despite the high performance obtained with such materials, they are compromised by high manufacturer costs, rigidity and high challenges for the mass fabrication of large area pixelated detector arrays, which could be used in flexible or curved substrates.^4^ As a result, their limited flexibility poses challenges for integration into wearable radiation monitors, portable medical devices, and curved imaging applications. While mechanical constraints remain a major bottleneck, there is also a growing strong emphasis on reducing the radiation doses in the medical imaging. Next generation detectors with lower detection limits would allow to use lower doses, thereby lowering cancer risks in computed tomography (CT) scans of *e.g.* the chest, which is known to cause about 2% of cancer diagnosed in the US.^5^ This highlights the urgent need for new generation radiation detectors with high sensitivity and lower detection limits to reduce radiation exposure without compromising imaging accuracy. Metal halide perovskites (MHP's) have marked considerable interest owing to their high atomic number, excellent X-ray absorbing property, low exciton binding energy, and unique electronic structure, which can accelerate the mobility and collection efficiency of charge carriers under a flexible form factor.^6–8^ On the other hand, organic–inorganic hybrid materials based on bismuth (Bi) have drawn interest as promising Pb-free alternatives. Based on variations in their composition and degree of crystallinity, the Young's moduli of MHPs range from 2.51 to 20 GPa, which is comparable to, or even lower than, those of typical polymers (0.92–4.3 GPa).^9^ This mechanical softness allows MHPs to better accommodate stress in flexible devices, thereby minimizing issues such as delamination. To leverage these excellent properties, researchers have developed Pb-based perovskite X-ray detectors in various forms, including single crystals,^10–19^ wafers/pellets,^20–25^ and films on/in both rigid and flexible substrates.^26–38^ Similarly, these emerging Bi-based materials offer remarkable X-ray detection performance across diverse forms, including single crystals,^39–43^ pellet/wafers,^44–51^ and flexible/rigid films.^52–55^ However, while acknowledging the potential of lead free alternatives, this study focuses on Pb-based perovskites. Among these forms (single crystals, pellets and thin/thick films), single crystals demonstrate outstanding performance due to their near perfect crystallinity and lack of grain boundaries. However, a significant challenge remains; single crystals are too fragile for integration into flexible substrates, and the fabrication method requires time demanding processes. Flexible X-ray detectors come with several advantages: they are lightweight, and can be bent onto uneven or curved surfaces, making them especially advantageous for wearable X-ray imaging applications, and potentially reducing cone-beam computed tomography (CBCT) distortion.^56^ Beyond reducing distortion, recent strategies adding organic frameworks and polymer matrices have significantly enhanced environmental robustness for new imaging possibilities. Notably, waterproof copper halide screens have enabled advanced 3D X-ray imaging *via* color and space reconfiguration.^57^ While solvent-free synthesis combined with polymer addition has facilitated stable and dual-functional flexible films for wireless communication and X-ray imaging.^58^ One common approach to achieve flexibility is to fabricate thin perovskite films, usually in the range of tens of nanometers up to a few micrometers, which have been successfully applied in devices such as flexible solar cells and LEDs.^59–61^ However, due to the exponential dependence of X-ray attenuation on thickness, substantially thicker films are required to achieve effective attenuation.^38^ To address this fundamental challenge of achieving sufficient thickness while maintaining flexibility, porous membranes have recently been applied as a substrate to improve the trade-off among mechanical flexibility, structural integrity, and detection performance.^9,62–66^ This membrane based approach has shown great promise in several early studies. For example, Liu *et al.* reported a high performance flexible detector based on MA_3_Bi_2_I_9_-filled nylon membranes, achieving a sensitivity of 2065 µC Gy_air_^−1^ cm^−2^ and an ultra-low detection limit of 2.71 nGy_air_ s^−1^.^64^ Zhao *et al.* fabricating perovskite infiltrated nylon membranes through a vacuum-assisted method that achieve an outstanding sensitivity of 8696 ± 228 µC Gy_air_^−1^ cm^−2^.^9^ Many reports typically utilize porous nylon membranes,^9,62,64,65^ and as a membrane filling strategy, use a process that adds time consuming and expensive steps, such as vacuum filtration. In some cases, dip coating has also been applied to simplify and expedite the process.^63^ In Fig. 1, several reported X-ray detectors are summarized and compared with the present work, including the state of the art of the Pb-based and non-Pb-based X-ray detectors in recent years. This work is highlighted by pink triangles, showing that our polymer-treated device outperforms previously reported flexible Pb-based and non-Pb-based X-ray detectors in terms of both sensitivity and detection limit, and even exceed commercial X-ray detectors. Despite these advances, a fundamental challenge remains. Developing a simple, scalable fabrication route for a flexible device that does not compromise on the high sensitivity characteristic of the best rigid detectors. In this work, we directly bridge this performance-flexibility gap by introducing polymer-perovskite composite architecture. We demonstrate that by embedding triple-cation perovskite solution into a mechanically robust and chemically inert hydrophilic Teflon (PTFE) membrane (∼200 µm) and free standing active layer can be fabricated *via* a simple spin coating process. The perovskite embedded membrane (PEM) device already achieves a high sensitivity of 0.9 × 10^5^ µC Gy_air_^−1^ cm^−2^ and a low detection limit of 0.46 nGy_air_ s^−1^ at bias voltage of 100 V. We then show that the incorporation of poly (methyl acrylate) (PMA) as a bulk additive is crucial for modulating perovskite crystallization and enhancing optoelectronic quality. Consequently, with the PMA additive, the sensitivity of our flexible detector dramatically increases to a remarkable 2.3 × 10^5^ µC Gy_air_^−1^ cm^−2^ while the detection limit improves to an ultra-low 0.09 nGy_air_ s^−1^ at 100 V bias, outperforming most flexible X-Ray detectors reported to date. We provide comprehensive evidence for the practical viability of this approach through extensive characterization of the devices’ high operational stability, device to device reproducibility, excellent durability under mechanical stress, and reliable operation over a broad X-ray energy spectrum (7–15 keV). In addition, PMA-PEM device exhibits shelf life stability, retaining ∼ 90.5% of its initial sensitivity even after 17 months of aging in mixed ambient/glovebox conditions.

## Results & discussion

### 1. Polymer selection, membrane choice, and film deposition

**Fig. 2:**
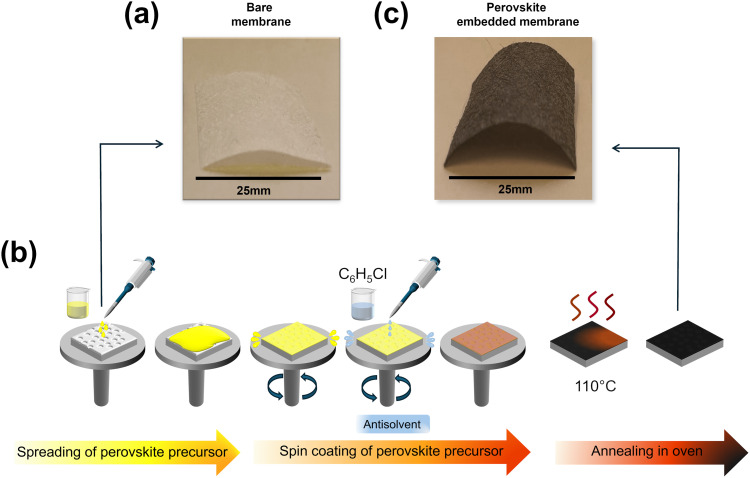
Fabrication process of flexible perovskite films. (a) Photograph of the bare membrane before the deposition of the perovskite precursor. (b) Schematic illustration of the spin coating process used to apply the perovskite precursor onto the membrane. (c) Photograph of the perovskite embedded membrane after the spin coating and annealing process, demonstrating the flexible structure of the final film.

**Fig. 3:**
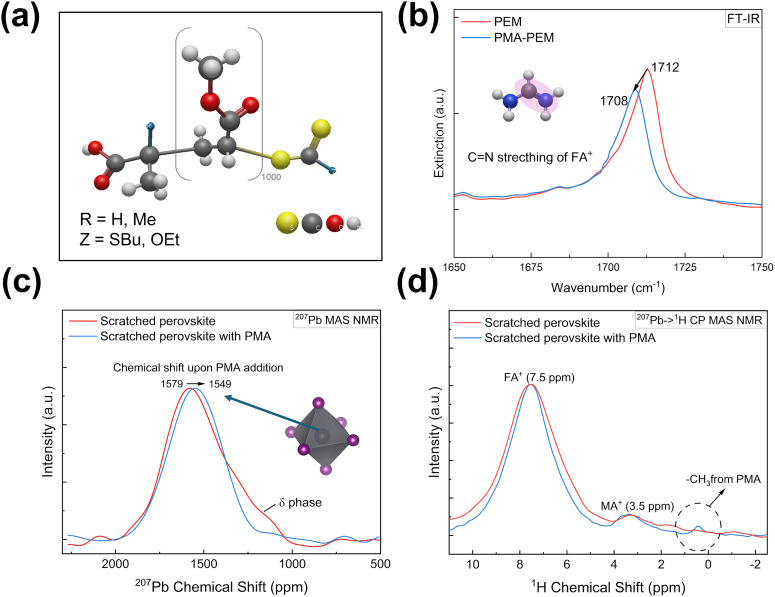
Spectroscopic evidence for the dual-action mechanism of PMA. (a) Chemical structure of the PMA additive. (b) FT-IR spectra showing a shift in the C=N stretching vibration upon PMA incorporation, indicating interaction with the organic cation. (c) ^207^Pb MAS NMR spectra revealing that PMA enhances phase stability (suppression of the δ-phase) and directly interacts with the Pb^2+^ lattice (chemical shift). (d) ^207^Pb → ^1^H CP-MAS NMR spectra confirming an interwoven nanostructure *via* the detection of PMA protons near Pb^2+^ nuclei.

To fabricate flexible PEMs, we chose hydrophilic PTFE membranes supported on polypropylene (PP) with high melting point, chemical resistance to dimethylformamide (DMF)/dimethyl sulfoxide (DMSO) solvent system and non-stick properties. In contrast, other substrates can compromise the perovskite layer, leading to reduced optoelectronic performance.^67^ PTFE is chemically inert, preventing protonation as well as other adverse reactions in the perovskite film. The PTFE membrane used in this study (see SI for supplier details) features a pore size of 0.45 µm at a thickness of ∼200 µm, as shown in the photograph in Fig. 2a. The infiltration is achieved through a facile spin-coating process, schematically illustrated in Fig. 2b, which enables the deposition of the triple-cation triple-halide perovskite Cs_0.05_(MA_0.02_FA_0.98_)_0.95_Pb(I_0.98_Br_0.02_Cl_*x*_)_3_ into the porous network. The resulting perovskite embedded membrane is shown in the photograph in Fig. 2c, confirming the successful formation of the active layer. During the deposition, we used an antisolvent treatment (chlorobenzene) to promote uniform rapid crystallization and subsequently annealed the coated membranes at 110 °C. To stabilize the precursor solution and regulate crystallization, PMA was introduced as a bulk additive. First, PMA was fully dissolved in a mixed solvent system of DMF and DMSO (5 : 1 v/v). Subsequently, the perovskite precursors (CsI, FAI, MABr, MACl, and PbI_2_) were added to the polymer solution. The final PMA concentration was fixed at 5 mg mL^−1^, a ratio optimized to yield uniform films without causing polymer precipitation. For this purpose, PMA was synthesized *via* xanthate-supported photo-iniferter (XPI)-reversible addition–fragmentation chain-transfer (RAFT) polymerization,^68^ as detailed in the SI. Fig. 3a shows the chemical structure of PMA, while the proton nuclear magnetic resonance (^1^H-NMR) spectrum and size exclusion chromatography (SEC) results for this polymer are shown in Fig. S1a and b. PMA was selected as an ideal additive over more common polymers like PMMA for our flexible device architecture. The established backbone structure of PMA can coordinate with perovskite through its carbonyl (C=O) group to Pb^2+^ and leads to enhanced carrier lifetimes and film morphology.^69^ While it shares the beneficial carbonyl group with PMMA for passivating Pb^2+^ ions,^70^ PMA is intrinsically more flexible. It lacks the alpha-methyl group found on the PMMA backbone, which reduces steric hindrance and chain rigidity, making PMA better suited to accommodate mechanical stress.^71^ Additionally, PMA's excellent solubility in our precursor solution was critical for achieving the uniform membrane embedding necessary for high performance devices.

### 2. Spectroscopic investigation of PMA-perovskite interaction

To understand the mechanism behind the PMA-PEM performance enhancement, we employed spectroscopic techniques, including Fourier transform infrared spectroscopy (FT-IR) and advanced solid-state NMR. The results, summarized in Fig. 3, provide direct evidence for dual interaction between the PMA polymer and both the organic and inorganic components of the perovskite. The interaction between PMA and the organic cations of the perovskite was first characterized by FT-IR spectroscopy (Fig. 3b). The spectrum of the PEM shows a peak at ∼1712 cm^−1^, which is characteristic of the C=N stretching vibration of the FA^+^ cation.^72–74^ Upon PMA incorporation, this peak clearly shifts to a lower wavenumber of ∼1708 cm^−1^. A shift in a vibrational frequency is a classic indicator of a change in a molecule's local chemical environment, and interpreting such shifts as proof of molecular interaction is a well-established method.^75^ This result therefore provides strong evidence of a direct interaction between the PMA polymer and the organic FA^+^ cation. To further corroborate the interaction with the FA^+^ cation observed in FT-IR, we also investigated the effect of PMA on the FAI solution using solution-state ^1^H NMR spectroscopy (Fig. S2a). The ^1^H NMR spectrum of FAI in DMSO-d_6_ shows two distinct signals for the FA^+^ cation: a singlet at ∼7.9 ppm corresponding to the imine (=CH) proton, and a doublet at ∼8.8 ppm corresponding to the amino (–NH_2_) protons.^76^ Upon the addition of PMA, the =CH proton peak broadened, with its FWHM nearly doubling from 0.011 ppm to 0.020 ppm, and more strikingly, the coupling constant of the amino proton doublet increased substantially from 78.8 Hz to 126.4 Hz. The splitting of FA^+^ protons is known to be related to the formation of hydrogen-bonding complexes.^77^ These changes therefore corroborate the FT-IR findings, strongly suggesting a direct interaction between the PMA and the FA^+^ cation in the solution phase, likely occurring *via* hydrogen bonding. To directly probe interactions between PMA and the inorganic perovskite lattice, we performed ^207^Pb MAS NMR on scratched films (Fig. 3c). The spectrum of the scratched perovskite exhibits the main perovskite resonance near +1550 ppm, but also displays an asymmetric shoulder around +1200 ppm, which is attributed to the δ-FAPbI_3_ degradation phase.^78^ Strikingly, this degradation related feature is completely suppressed in the scratched perovskite with PMA, which shows a very symmetric line shape, providing clear evidence that PMA enhances the phase stability of the perovskite. Furthermore, the main resonance shifts from 1579 ppm in the scratched perovskite to 1549 ppm with PMA. Since the ^207^Pb chemical shift is highly sensitive to the local electronic environment, this shift confirms a direct interaction between the polymer and the Pb^2+^ ions, which alters the average electronic structure of the perovskite lattice. Direct excitation ^1^H MAS NMR was used to confirm the film compositions (Fig. S2b) revealing the expected presence of FA^+^ and MA^+^ (at 7.5 ppm and 3.5 ppm) cations as well as of PMA (at 0.5 ppm) for PMA-treated samples. Finally, to definitively probe the spatial proximity between the polymer and the perovskite lattice, we utilized ^207^Pb → ^1^H cross-polarization (CP)-MAS NMR (Fig. 3d), a technique that selectively detects protons within nanometers of the lead atoms. The resulting spectrum of the scratched perovskite with PMA reveals two critical features that are not present in the scratched perovskite: a new resonance at 0.5 ppm, assigned to the PMA methyl groups, and a significant narrowing of the FA^+^ cation peak at 7.5 ppm. The detection of the PMA signal is direct and strong evidence for interwoven structure at the nanoscale, ruling out the hypothesis of large, segregated domains. In parallel, the narrowing of the FA^+^ peak indicates that PMA also creates a more uniform and ordered local environment for the organic cations. Together, these results confirm a true molecular level integration and a dual-action interaction that affects both the inorganic lattice and the organic cations. Specifically, we highlighted that the reduced steric hindrance around the carbonyl group in PMA resulting from the absence of the alpha-methyl group present in PMMA facilitates stronger coordination with Pb^2+^ defects, a structural advantage supported by previous studies on acrylate side-group orientations.^79^ Fig. S3c shows ^207^Pb → ^1^H CP-MAS NMR spectra of PMA-PEM signal where a maximum at 1.3 ppm is observed, whereas no sharp signal is observed in that range for the scratched perovskite with or without PMA. This is the position of the maximum for the ^1^H direct polarization peak of the pure membrane (Fig. S3a). These results indicate that perovskite crystallites were successfully grown in the pores of Teflon membrane supported on polypropylene.

### 3. Infiltration and morphology

**Fig. 4:**
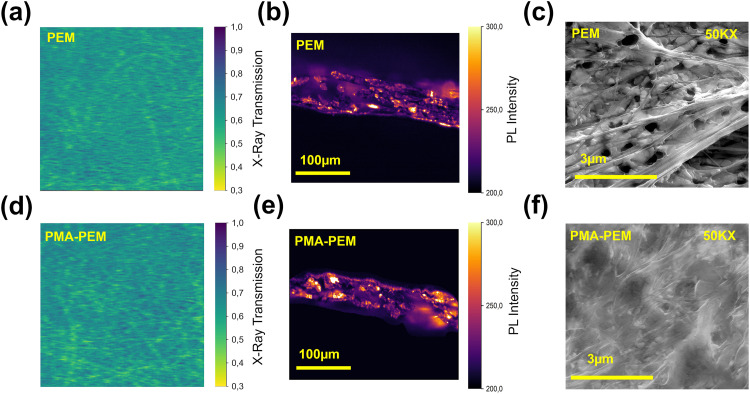
X-ray transmission, cross-sectional photoluminescence (PL), and scanning electron microscopy (SEM) images of perovskite embedded membranes with and without PMA. (a) and (d) X-ray transmission images of the PEM and PMA-PEM, respectively, show no significant difference in transmitted X-ray intensity, indicating similar perovskite loading in both films. (b) and (e) Cross-sectional PL images of the PEM and PMA-PEM under blue light illumination show a slight increase in PL intensity for the PMA-PEM, suggesting improved perovskite crystallization and reduced non-radiative recombination. (c) and (f) SEM images at high magnification illustrate morphological differences: the control film exhibits a more granular structure with distinct perovskite domains, while the PMA-PEM appears more uniform and interconnected, implying enhanced perovskite distribution and improved film formation.

To gain insight on the role of PMA for perovskite infiltration, we measured X-ray transmission images and cross-sectional photoluminescence images. As displayed in Fig. 4a and d, these reveal similar X-ray absorption suggesting comparable infiltration for PEM and PMA-PEM. The cross-sectional photoluminescence (PL) images of the two Fig. 4b and e show comparable emission intensities between PEM and PMA-PEM, with just a slight increase in the membrane with PMA. However, scanning electron microscopy (SEM) reveals significant differences in the quality of the film and microstructure. Top view SEM images at high magnification (Fig. 4c and f) show that while the PEM consists of dispersed perovskite aggregates with noticeable voids, the PMA-PEM exhibits a much smoother, more continuous, and interconnected morphology. This enhanced large area uniformity of the PMA-PEM is further highlighted in low magnification SEM images provided in Fig. S4. This morphological improvement from isolated aggregates in PEM to a dense, continuous layer in PMA-PEM is driven by the polymer-assisted modulation of crystallization dynamics. Specifically, the morphological improvement stems from two key mechanisms supported by the interaction between PMA and the perovskite precursors. First, the incorporation of the polymer modifies the rheological properties of the precursor solution. As described in recent studies on polymer-perovskite composites, adding long chain polymers typically transitions the fluid behavior from Newtonian to pseudoplastic.^80^ This rheological modification is critical; the pseudoplastic nature allows the solution to spread easily under shear force, but immediately regain high viscosity once spin coating ends. This prevents the solution from retracting or dewetting on the porous membrane surface, ensuring that the material forms a stable, continuous liquid film rather than breaking into discontinuous islands.^80^ Second, PMA acts as a supramolecular template that regulates nucleation. Consistent with findings on biopolymer-mediated crystallization, the coordination between the polymer functional groups and lead species induces the disruption of colloidal aggregates in the solution phase.^81^ This suppression of uncontrolled aggregation promotes a heterogeneous nucleation mechanism, where the polymer network guides the growth of interconnected perovskite grains and fills the interstitial voids (binder effect).^80,81^ Consequently, PMA effectively stitches the crystallites into a continuous composite layer. This structural interconnectivity stabilizes the morphology against crack propagation and delamination, resulting in the superior film continuity observed in SEM without requiring an increase in total material loading. Such improvements in film morphology are consistent with previous reports, where carbonyl rich polymers such as poly(vinyl alcohol) (PVA), poly(acrylic acid) (PAA), and PMA have been shown to alter the crystallization kinetics and help guide nucleation pathways and thereby improving the uniformity and reducing pinholes of the films.^69^ Further cross-sectional SEM micrographs of PEM and PMA-PEM can be found in Fig. S5 and Fig. S6. Ideally, the active layer thickness in this porous membrane should be defined by the effective absorber thickness rather than the geometric membrane thickness. While SEM cross-sections indicate a geometric thickness of 200 µm (porous membrane), the actual amount of X-ray absorbing material was quantified *via* gravimetric analysis. The average perovskite mass loading was determined to be 11.60 mg cm^−2^. Based on the theoretical density of the triple-cation perovskite 4.09 g cm^−3^, this corresponds to an equivalent active thickness (*t*_eq_) ∼28 µm. Therefore, the perovskite occupies approximately 14.2% of the total membrane volume (detailed data in Table S4). As shown in Fig. S7b, the attenuation efficiency curves indicate that the triple-cation perovskite achieves >95% attenuation with a thickness of only ∼28 µm at 8 keV. This performance significantly outperforms Si and is comparable to CdTe, highlighting its potential for use in low-energy X-ray detection.

### 4. Structural and optoelectronic properties

**Fig. 5:**
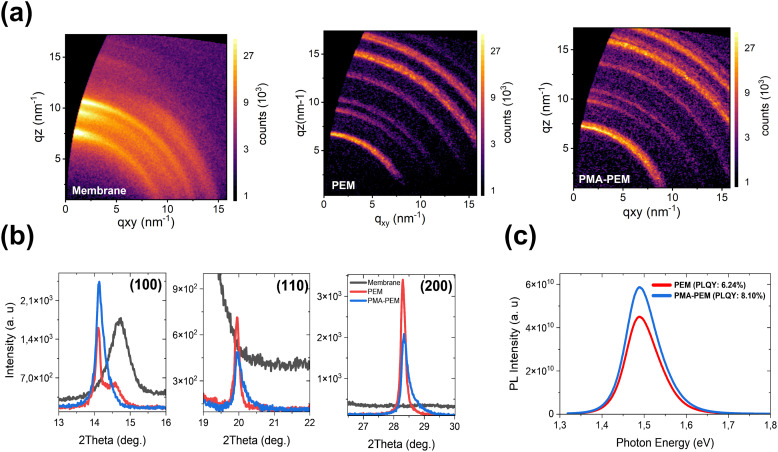
Structural and optoelectronic characterization of PEM and PMA-PEM. (a) Grazing incidence wide-angle X-ray scattering (GIWAXS) patterns of bare membrane (left), PEM (middle), and PMA-PEM (right). (b) X-ray diffraction patterns of the bare membrane, PEM and PMA-PEM, comparing the (100), (110), and (200) crystal planes. The PMA-PEM exhibits an enhanced (100) peak without a shift in peak position, suggesting improved crystallinity without phase transition. (c) Photoluminescence (PL) spectra of flexible films, showing an increase in PL intensity and photoluminescence quantum yield (PLQY) from 6.2% (PEM) to 8.1% (PMA-PEM), indicating reduced non-radiative recombination.

To further investigate the impact of PMA on perovskite crystallization and optoelectronic properties, we performed grazing incidence wide-angle X-ray scattering (GIWAXS). GIWAXS patterns (Fig. 5a) show that the incorporation of PMA intensifies the diffraction signal at *q*_*z*_ ≈ 10 nm^−1^, corresponding to the (100) crystallographic plane. Notably, no shift is observed in any of the peak positions. The increased intensity of the diffraction rings suggests that there is an increased preferential orientation along the (100) plane. Bragg–Brentano (B–B) X-ray diffraction (XRD) scans (Fig. 5b) further corroborate that PMA enhances crystallinity, evidenced by higher (100) and lower (110) and (200) diffraction peaks, indicating that it stabilizes the perovskite structure rather than inducing a new phase or lattice strain. A closer look at the (100) diffraction peak reveals a broad diffraction peak from the bare membrane (gray curve) that overlaps with the higher angle side of the (100) diffraction peak. For the PEM sample (red curve), a small shoulder is evident at this position, directly correlating with the peak from the underlying membrane support. This finding suggests that X-rays are diffracting from the membrane support through pinholes or discontinuities in the perovskite layer. Notably, this shoulder is entirely absent in the PMA-PEM (blue curve). This result indicates that PMA provides a more complete and uniform coverage, effectively masking the diffraction signal from the membrane support. This finding is in good agreement with our SEM observations, which showed a denser, more continuous film morphology for PMA-PEM sample. These structural improvements in our system are a direct consequence of the dual-action interaction mechanism we identified in the interaction section. The interaction of PMA with both the Pb^2+^ ions and FA^+^ cations effectively modulates the crystallization and slows down nucleation, promoting controlled growth along more thermodynamically stable facets, leading to a more ordered and preferentially oriented film.^82,83^ Next, we evaluated the optoelectronic quality of PEM and PMA-PEM by measuring their photoluminescence (Fig. 5c). The incorporation of PMA leads to significant enhancement in PL intensity. This is quantitatively confirmed by the photoluminescence quantum yield (PLQY), which increased from 6.2% for the PEM to 8.1% for the PMA-PEM. This enhancement in PLQY is a clear indicator of reduced non-radiative recombination losses, and has been previously reported for PMA additives.^69^ This reduction of defects can be directly attributed to the passivation of defect sites (such as uncoordinated Pb^2+^) and the improved structural order resulting from the interwoven nature of the PMA and perovskite, as proven by our NMR analysis. This result is consistent with the established principle that suppressing trap-mediated, non-radiative recombination enhances carrier lifetime and the mobility-lifetime (µτ) product, which directly correlates with improved sensitivity and lower detection limits in perovskite X-ray detectors.^84–86^

### 5. Fabrication of perovskite embedded membrane device and their X-ray performance under continuous and pulsed source illumination

#### 5.1. Device fabrication

**Fig. 6:**
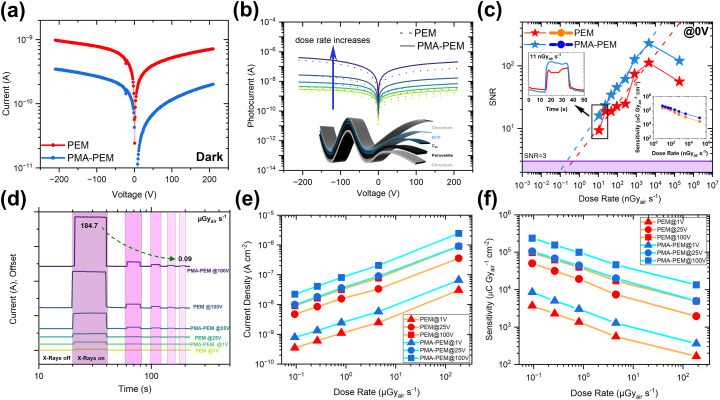
Electrical and X-ray detection performance of PEM and PMA-PEM devices under continuous Cu anode irradiation (primary Kα (8.05 keV) emission line). (a) Dark current–voltage characteristics of PEM and PMA-PEM devices, demonstrating lower dark current with PMA. (b) Current–voltage characteristics under continuous Cu anode irradiation, showing increasing photocurrent with dose rate for both PEM and PMA-PEM devices, and the enhanced response of the PMA-PEM device. The inset shows the device structure. (c) Signal to noise ratio (SNR) as a function of dose rate at 0 V bias, highlighting the enhanced performance of PMA-PEM device and its lower detection limit compared to PEM. Inset shows the current response at a dose rate of 11 nGy_air_ s^−1^ and sensitivity trends as a function of dose rate at 0 V bias. (d) Current response of the devices as a function of dose rate at different bias voltages (1 V, 25 V, and 100 V) (e) Current density as a function of dose rate at different bias voltages (1 V, 25 V, and 100 V). (f) Sensitivity as a function of dose rate at different bias voltages (1 V, 25 V, and 100 V), indicating superior sensitivity in PMA-PEM device across all conditions.

Having achieved uniform infiltration of the PTFE membranes using PMA-treated triple-cation, triple-halide perovskites, we fabricated flexible X-ray detectors by thermally depositing chromium (Cr) and a C_60_/bathocuproine (BCP)/Cr contact stack on the back and front sides, respectively, following the structure reported by Zhao *et al.*^9^ The final device architecture is shown in the inset of Fig. 6b. The active area of the devices was 0.06 cm^2^, defined by the overlap between the top and bottom electrodes.

#### 5.2. X-ray performance under continuous source illumination

Fig. 6a presents dark current–voltage (*I*–*V*) characteristics of PEM and PMA-PEM devices, revealing a lower dark current in detectors utilizing PMA. The addition of PMA facilitates the formation of homogenous and compact perovskite film with minimized pinholes that physically reduce leakage paths. Thus, this leads to higher shunt resistance. Moreover, the interaction of the carbonyl groups of PMA with defects effectively passivates trap states. In addition, the improved crystallinity confirmed by XRD indicates reduction in bulk defects. This minimizes thermally generated carriers, leading to lower dark current. The enhanced PL intensity and PLQY observed in PMA-PEM films show that a decrease in the density of non-radiative recombination centers (traps). Collectively, this minimized defect density results in lowering the dark current. We then investigated the X-ray response under varying exposure conditions by recording *I*–*V* characteristics at multiple dose rates (0.09 to 184.7 µGy_air_ s^−1^) for both the PEM and PMA-PEM devices under continuous Cu anode irradiation (primary Kα (8.05 keV) emission line) without encapsulation of the device. The results show an increase in photocurrent, see Fig. 6b with an increasing dose rate, confirming the consistent X-ray responsivity of both devices. Notably, the PMA-PEM device demonstrates a higher photocurrent across all dose rates. Investigating the sensitivity as a function of applied bias voltage under continuous Cu anode irradiation at the dose rate of 184.7 µGy_air_ s^−1^ reveals a clear trend of increasing sensitivity with voltage, with the PMA-PEM device outperforming the PEM across the entire voltage range as shown in Fig. S8c. This behavior suggests that a stronger internal electric field facilitates more efficient charge transport and extraction. Notably, both detectors allow reliable operation under both reverse and forward biases even up to a high voltage of ±200 V that enables high sensitivity, see also photocurrent response measurements during repeated X-ray on/off switching displayed in Fig. S8(a,b) and S9(a,b). Furthermore, although the typical operating bias does not exceed ±200 V, the detectors remained electrically stable under a continuously increasing bias from 0 to +1000 V, highlighting their exceptional dielectric strength. As shown in Fig. S8d, no breakdown was observed under dark conditions. In addition to the high voltage stability that previously has been associated with thick single crystal or pressed pellet based devices, our flexible membrane detectors also allow the self-powered operation of the detectors, as indicated in Fig. S10 displaying time dependent photocurrent response of both PEM and PMA-PEM devices at different X-ray dose rates (0.01–184.7 µGy_air_ s^−1^) at 0 V bias. These measurements demonstrate stable and reproducible on/off switching behavior. This self-powered capability arises from the asymmetric device architecture, depicted in the inset of Fig. 6b. The asymmetry is also evident in the dark current–voltage characteristics (Fig. 6a), which show clear rectifying behavior. This is because the different work functions of the bottom electrode (Cr) and the top contact stack (C_60_/BCP/Cr) create a built-in potential across the perovskite absorber layer. This internal field is sufficient to facilitate the separation and collection of X-ray-generated charge carriers even at zero applied external bias, enabling the self-powered operation. Fig. 6c presents the 0 V bias detection performance of the devices, highlighting the impact of PMA on sensitivity and detection limits. The PMA-PEM detector achieves a significantly lower limit of detection (LoD) of 0.22 nGy_air_ s^−1^, compared to 0.46 nGy_air_ s^−1^ for PEM. Additionally, the exceptional sensitivities are 4.2 × 10^3^ µC Gy_air_^−1^ cm^−2^ for PMA-PEM and 2.2 × 10^3^ µC Gy_air_^−1^ cm^−2^ for PEM, demonstrating a two-fold improvement by using PMA as bulk additive even under the dose rate of 11 nGy_air_ s^−1^.The insets illustrate the current response at the lowest dose rate and the sensitivity trend *vs.* dose rate, further confirming the superior low dose detection capability. Next, we investigated the X-ray response at 1 V, 25 V, and 100 V applied bias over a broad dose rate range (0.09 to 184.7 µGy_air_ s^−1^) to showcase robust detector performance across different bias and dose rate conditions. As shown in the time dependent current response in Fig. 6d, the PMA-PEM detectors deliver a higher photocurrent response across all bias voltages and throughout the full range of X-ray dose rates, down to 0.09 µGy_air_ s^−1^. Consistently, the current density *versus* dose rate curves in Fig. 6e further confirm this enhancement, revealing that the PMA-PEM devices generate significantly higher photocurrent than the PEM devices at each dose rate and bias condition. Fig. 6f presents the sensitivity of the detectors as a function of X-ray dose rate at different voltages. The ability to operate effectively at 1 V bias, that is, at almost/practically zero bias, further highlights the potential of these detectors for low power X-ray detection applications. Maintaining low operation voltages further will be crucial to ensure long-term stability for commercial products using halide perovskites where ion migration, electromigration of contact materials, and electrochemical degradation of the active halide perovskite material may impact stability.^87–90^ At a low dose rate of 0.09 µGy_air_ s^−1^ under 1 V bias, the PMA-PEM detector achieves a sensitivity of 8 × 10^3^ µC Gy_air_^−1^ cm^−2^, compared to 4 × 10^3^ µC Gy_air_^−1^ cm^−2^ for the PEM. At 100 V bias, the sensitivity increases to 2.3 × 10^5^ µC Gy_air_^−1^ cm^−2^ for the PMA-PEM device, significantly higher than the 0.9 × 10^5^ µC Gy_air_^−1^ cm^−2^ of PEM. We note that the trend of increasing sensitivity with decreasing dose rate (Fig. 6f) is attributed to a gain factor enhancement at lower X-ray intensities, commonly observed for perovskites and PbS quantum dot-based photodetectors.^91^ Such behavior is typically linked to enhanced photoconductive gain mechanisms, arising from long-lived carriers and injection assisted amplification under bias.^92^ We extrapolated the limit of detection (LoD), according to the IUPAC standard where LoD is defined as the dose rate at which the signal-to-noise ratio (SNR) reaches 3,^93^ see also Fig. S12. At a bias of 100 V, the LoD values were approximately 0.09 nGy_air_ s^−1^ for the PMA-PEM device and 0.46 nGy_air_ s^−1^ for PEM device. As evident in Fig. 1, these values are low especially in comparison with all the Pb and non Pb-based flexible detectors. They also lie below the typical medical imaging threshold of 5.5 µGy_air_ s^−1^. These ultra-low detection limits make these devices promising candidates for next generation radiation detection applications, where high sensitivities at low levels of exposure are required.

#### 5.3. Device stability, reproducibility and durability

**Fig. 7:**
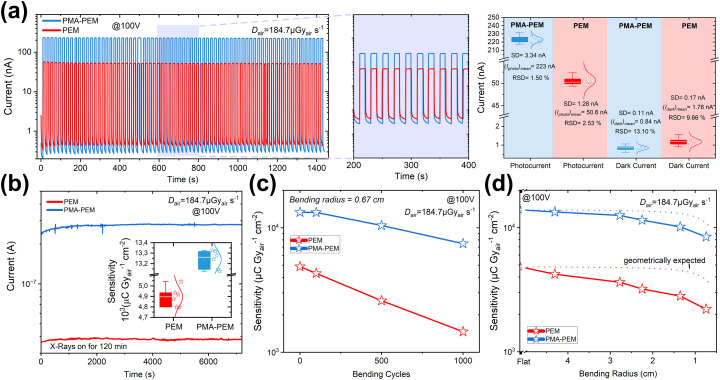
Operational stability, device to device reproducibility, and mechanical robustness of PEM and PMA-PEM X-ray detectors. (a) Time-resolved current response during 70 X-ray on/off cycles at 100 V bias and the dose of 184.7 µGy_air_ s^−1^. Right: Statistical summary of photocurrent and dark current. The PMA-PEM device exhibits a significantly higher mean photocurrent (223 nA) with lower RSD (1.50%) compared to the PEM (50.6 nA, RSD = 2.53%). PMA-PEM also shows lower dark current (0.84 nA *vs.* 1.76 nA). (b) Long term continuous X-ray exposure for 120 minutes confirms stable operation without degradation, with PMA-PEM consistently generating higher current. (c) Sensitivity retention after cyclic bending fatigue (1000 bending cycles, *R* = 0.6725 cm), where PMA-PEM retains ∼56% of its initial sensitivity, outperforming PEM (∼30%). (d) Static bending test results under varying curvature. PMA-PEM maintains ∼60.5% of its sensitivity at the bending radius of 0.741 cm. When corrected for projected pixel area, the retention improves to ∼77.6%, compared to ∼58.3% for PEM, highlighting the mechanical durability of PMA-PEM devices.

Beyond high sensitivity, the practical viability of the detectors was assessed through a comprehensive evaluation of their long-term stability under irradiation, device to device reproducibility, and mechanical durability. All tests were performed on non-encapsulated devices under ambient condition, and the results are summarized in Fig. 7. First, device stability was evaluated under both pulsed and continuous X-ray exposure (Fig. 7a and b). During 70 repeated X-ray on/off cycles at 100 V bias and exposed the X-ray dose of 184.7 µGy_air_ s^−1^ (Fig. 7a), both PEM and PMA-PEM devices exhibited highly stable and reproducible photocurrent responses. The PMA-PEM device demonstrated superior operational stability, with a mean photocurrent of 223 nA, approximately 4.4 times higher than that of PEM (50.6 nA) and a relative standard deviation (RSD) of only 1.50%, compared to 2.53% for PEM. Additionally, dark current measurements revealed a significant reduction in baseline noise for the PMA-PEM architecture, with a mean dark current of 0.84 nA and RSD of 13.10%, compared to 1.76 nA and 9.66% for PEM. This corresponds to an increase in dark resistance from 5.68 × 10^10^ Ω (PEM) to 1.19 × 10^11^ Ω (PMA-PEM) at 100 V. Under extended continuous X-ray exposure of 120 minutes (Fig. 7b), both detectors maintained stable photocurrent output with no observable degradation, while the PMA-PEM consistently delivered higher current levels throughout the irradiation period. Next, device to device reproducibility was statistically analyzed across a range of dose rates (0.09–184.7 µGy_air_ s^−1^), using six independently fabricated devices per architecture. The inset in Fig. 7b shows a representative box plot of sensitivity distributions at 184.7 µGy_air_ s^−1^ under 100 V bias. Full distributions are provided in Fig. S13, clearly revealing the enhanced mean sensitivity and narrower statistical distribution of the PMA-PEM devices. As summarized in Table S5, the PMA-PEM detectors exhibited excellent reproducibility, with RSD values in sensitivity remaining below 1% in most cases and never exceeding 0.85%. Finally, mechanical durability was assessed via both cyclic bending fatigue and static bending tests (Fig. 7c and d). In the fatigue test, after 1000 bending cycles at a fixed radius of 0.67 cm, the PMA-PEM retained approximately 56% of its initial sensitivity, while PEM retained only ∼30% (Fig. 7c). Table S6 provides a detailed summary of sensitivity and corresponding retention values at various bending cycles. In static bending tests, performance was measured under decreasing bending radii. At the smallest bending radius of 0.741 cm, the PMA-PEM retained ∼60.5% of its initial sensitivity, significantly outperforming PEM, which retained only ∼45.5% (Fig. 7d). Since these measurements were performed while the devices were bent, the effective pixel area was geometrically reduced. Corrected sensitivity values, using the projected pixel length under curvature, reveal that the PMA-PEM retained ∼77.6% of its initial sensitivity at the bending radius of 0.741 cm, compared to ∼58.3% for the PEM. These corrected results, presented also in Table S7 alongside a detailed description of the correction (SI, eqn (S7)), further confirm the superior mechanical integrity and strain resilience of the PMA-PEM architecture. To examine the mechanical durability, we performed SEM analysis on films after 1000 bending cycles (Fig. S14). The PEM films show brittleness, characterized by numerous wide, deep cracks. In contrast, the PMA-PEM films remain largely intact. While minor damage is expected, the cracks in the PMA-PEM films are significantly fewer and appear only as narrow, hairline fractures, unlike the wide gaps seen in the PEM. This indicates that the crack widening and propagation are effectively suppressed. We attribute this resistance to the synergistic interplay between the viscoelastic state and chemical functionality of the PMA additive. First, unlike rigid polyacrylates such as PMMA (glass transition temperature (*T*_g_) ∼105 °C),^94^ PMA possesses a low glass transition temperature (*T*_g_ ∼8 °C),^95^ ensuring it remains in a highly viscoelastic, rubbery state at room temperature. As highlighted in recent studies on flexible perovskites, elevated viscoelasticity is critical for facilitating interfacial adhesion and dissipating mechanical stress.^96^ Crucially, the PMA is not merely confined to interfaces but forms a homogeneously distributed network throughout the perovskite film, as illustrated in the schematic in Fig. S15. Within this network, the carbonyl (C=O) groups chemically anchor to uncoordinated Pb^2+^ ions, while the flexible polymer chains act as a bulk molecular shock absorber. This homogenously distributed viscoelastic reinforcement effectively bridges the micro cracks, preventing them from opening into wide fractures and ensuring high sensitivity retention under static and cyclic bending fatigue. To address long term reliability, we remeasured unencapsulated devices aged for 17 months in mixed ambient/glovebox conditions. Remarkably, PMA**-**PEM devices demonstrated exceptional long term stability after 17 months of aging (Fig. S16), They exhibited a suppressed dark current increase of only ∼2.2-fold (*vs.* ∼4.5-fold for PEM) and retained ∼90.5% of their initial sensitivity, significantly outperforming PEM devices (∼74% retention).

#### 5.4. X-ray energy dependent response

**Fig. 8:**
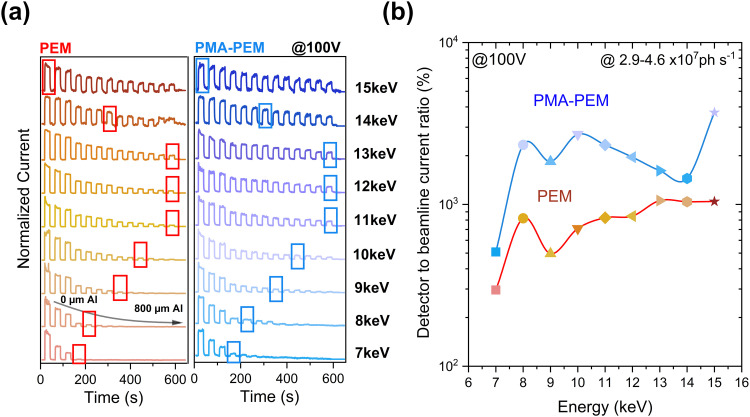
X-ray energy-dependent detection performance of PEM and PMA-PEM devices at BESSY II. (a) Time dependent normalized current response of PEM and PMA-PEM devices at 100 V bias under pulsed X-ray irradiation at different photon energies (7–15 keV). The PMA-PEM devices exhibit higher and more stable responses across all energies. Highlighted boxes indicate the response peaks corresponding to selected irradiation conditions. (b) Detector to beamline current ratio (%) as a function of X-ray energy, demonstrating enhanced response in PMA-PEM devices across the measured energy range.

To investigate the energy-dependent response of the detectors, we conducted experiments at the time-resolved hard X-ray diffraction end station (KMC-3 XPP) at BESSY II, which allows excitation with monochromatic X-rays covering an energy range of 2 keV to 16 keV utilizing a monochromatic X-ray beam. The X-ray beam at BESSY II exhibits ∼50 ps-long pulses spaced by 2 ns, with a short gap of 56 ns containing only a single bunch middle.^97^ The detailed experimental setup illustrated and described in Fig. S17. The intensity of the X-rays varies with X-ray energy according to the characteristic bending magnet output and was adjusted using aluminum absorbers of different thicknesses, see Fig. S18. We then recorded the X-ray photocurrent, for different X-ray energies as a function of time, while increasing the Al-absorber thickness during X-ray off periods. The resulting photocurrent was normalized to the maximal X-ray photon current at each photon energy without absorber, shown in Fig. 8a. This reveals reliable X-ray detection across all photon energies, considering the increasing X-ray attenuation of the Al absorber at low photon energies. Additionally, we wish to emphasize outstanding dark current stability in the ambient (non-encapsulated) condition during nearly 2 hours of repeated X-ray on/off cycling at different photon energies and intensities. Consistent and reproducible dark current levels were observed for the same set of PEM and PMA-PEM devices throughout the week long beamtime, despite being kept under ambient (non-encapsulated) conditions for the entire duration. In order to gain insight into energy dependent response of the devices, we evaluated the detector to beamline current ratio at bias of 100 V (see Fig. 8b) by choosing, as indicated by the boxes in Fig. 8a, comparable photon flux values of (2.9–4.6) × 10^7^ ph s^−1^. Fig. S19a and b present the corresponding data acquired at 25 V bias, using the same methodology as in Fig. 8a and 8b. Across all photon flux levels, the detector current at both 25 V and 100 V biases was normalized to the incident photon current (derived from the beamline flux) to determine the device efficiency across the tested energy range (Fig. S19c,d). We demonstrate that both detectors can detect X-ray energies in the range of 7–15 keV. As illustrated in Fig. 8a,b and S19a,b, the PMA-PEM detector consistently yields higher photocurrent and detector to beamline current ratio than the PEM device across all tested bias voltages, X-ray energies, and photon flux levels. This is in line with the performance of the PMA based devices that we have observed using our continuous Cu anode X-ray source (primary Kα (8.05 keV) emission line).

## Conclusion

In conclusion, we have demonstrated a highly effective strategy for fabricating high-performance flexible X-ray detectors by incorporating PMA as bulk additive into a triple cation perovskite embedded Teflon membrane. Through the comprehensive spectroscopic investigation combining both solution-state and solid-state NMR, alongside FT-IR analysis, we provided direct evidence for the dual-action interaction mechanism, where PMA passivates the inorganic Pb^2+^ lattice to enhance phase stability while also interacting with the organic FA^+^ cations to promote a more ordered crystal structure. This cascade of improvements at the molecular level results in a device with not only exceptional sensitivity (2.3 × 10^5^ µC Gy_air_^−1^ cm^−2^) and an ultra-low detection limit (0.09 nGy_air_ s^−1^) at 100 V bias, but also efficient self-powered operation at 0 V bias with the sensitivity of 4.2 × 10^3^ µC Gy_air_^−1^ cm^−2^. This high performance is coupled with proven reproducibility, long term stability, and mechanical durability, as well as reliable operation over a broad X-ray energy spectrum (7–15 keV). This work establishes polymer embedded membrane scaffolds as a robust and promising platform for next-generation, practical X-ray detection technologies.

## Conflicts of interest

There are no conflicts to declare.

## Supplementary Material

MH-013-D5MH02084K-s001

## Data Availability

The data supporting this article have been included as part of the main text and supplementary information (SI). Supplementary information includes a comprehensive set of additional figures and tables, detailed synthesis protocols, literature comparison data, and further characterization/analysis results. See DOI: https://doi.org/10.1039/d5mh02084k.

## References

[cit1] Knoll G. F. , Radiation Detection and Measurement, 4th edn, John Wiley & Sons, Inc, New York, 2010, pp. 402–437

[cit2] Kabir M. Z., Yunus M., Kasap S. O., Tousignant O., Mani H., Gauthier P. (2006). Sensitivity of Stabilized A-Se Based X-Ray Photoconductors. Curr. Appl. Phys..

[cit3] Takahashi T., Watanabe S. (2001). Recent Progress in CdTe and CdZnTe Detectors. IEEE Trans. Nucl. Sci..

[cit4] Basiricò L., Ciavatti A., Fraboni B. (2021). Solution-Grown Organic and Perovskite X-Ray Detectors: A New Paradigm for the Direct Detection of Ionizing Radiation. Adv. Mater. Technol..

[cit5] Lin E. C. (2010). Radiation Risk from Medical Imaging. Mayo Clin. Proc..

[cit6] Wang D., Li G. (2022). Advances in Photoelectric Detection Units for Imaging Based on Perovskite Materials. Laser Photonics Rev..

[cit7] Fan J., Li W., Zhou Q., Yang G., Tang P., He J., Ma L., Zhang J., Xiao J., Yan Z., Li A., Han X. (2025). Metal Halide Perovskites for Direct X-Ray Detection in Medical Imaging: To Higher Performance. Adv. Funct. Mater..

[cit8] Geng X., Chen Y. A., Li Y. Y., Ren J., Dun G. H., Qin K., Lin Z., Peng J., Tian H., Yang Y., Xie D., Ren T. L. (2023). Lead-Free Halide Perovskites for Direct X-Ray Detectors. Adv. Sci..

[cit9] Zhao J., Zhao L., Deng Y., Xiao X., Ni Z., Xu S., Huang J. (2020). Perovskite-Filled Membranes for Flexible and Large-Area Direct-Conversion X-Ray Detector Arrays. Nat. Photonics.

[cit10] He Y., Hadar I., De Siena M. C., Klepov V. V., Pan L., Chung D. Y., Kanatzidis M. G. (2022). Sensitivity and Detection Limit of Spectroscopic-Grade Perovskite CsPbBr3 Crystal for Hard X-Ray Detection. Adv. Funct. Mater..

[cit11] Song Y., Li L., Bi W., Hao M., Kang Y., Wang A., Wang Z., Li H., Li X., Fang Y., Yang D., Dong Q. (2020). Atomistic Surface Passivation of CH 3 NH 3 PbI 3 Perovskite Single Crystals for Highly Sensitive Coplanar-Structure X-Ray Detectors. Research.

[cit12] Wei H., Desantis D., Wei W., Deng Y., Guo D., Savenije T. J., Cao L., Huang J. (2017). Dopant Compensation in Alloyed CH3NH3PbBr3-x Clx Perovskite Single Crystals for Gamma-Ray Spectroscopy. Nat. Mater..

[cit13] Liu Y., Zhang Y., Zhu X., Feng J., Spanopoulos I., Ke W., He Y., Ren X., Yang Z., Xiao F., Zhao K., Kanatzidis M., Liu S. (2021). Triple-Cation and Mixed-Halide Perovskite Single Crystal for High-Performance X-Ray Imaging. Adv. Mater..

[cit14] Chu D., Jia B., Liu N., Zhang Y., Li X., Feng J., Pi J., Yang Z., Zhao G., Liu Y., Liu S., Park N. G. (2023). Lattice Engineering for Stabilized Black FAPbI3 Perovskite Single Crystals for High-Resolution x-Ray Imaging at the Lowest Dose. Sci. Adv..

[cit15] Huang Y., Qiao L., Jiang Y., He T., Long R., Yang F., Wang L., Lei X., Yuan M., Chen J. (2019). A-Site Cation Engineering for Highly Efficient MAPbI3 Single-Crystal X-Ray Detector. Angew. Chem., Int. Ed..

[cit16] Jiang W., Li H., Liu D., Ren J., Zhao Y., Wu J., Chen J., Zhou L., Wang F., Zhao Y. (2024). Synergetic Electrostatic and Steric Effects in α-FAPbI3 Single Crystals For X-Ray Detection and Imaging. Small.

[cit17] Wang L., Yan Y., Bu M., Wang J., Li L., Li Y., Liu H., Zhang H., Pi X., Yang D., Fang Y. (2024). Silicone-Assisted Autonomous Growth of Strainless Perovskite Single Crystals for Integrated Low-Dose X-Ray Imaging Arrays. Adv. Funct. Mater..

[cit18] Song Y., Li L., Hao M., Bi W., Wang A., Kang Y., Li H., Li X., Fang Y., Yang D., Dong Q. (2021). Elimination of Interfacial-Electrochemical-Reaction-Induced Polarization in Perovskite Single Crystals for Ultrasensitive and Stable X-Ray Detector Arrays. Adv. Mater..

[cit19] Wu J., Wang L., Feng A., Yang S., Li N., Jiang X., Liu N., Xie S., Guo X., Fang Y., Chen Z., Yang D., Tao X. (2022). Self-Powered FA0.55MA0.45PbI3 Single-Crystal Perovskite X-Ray Detectors with High Sensitivity. Adv. Funct. Mater..

[cit20] Xiao Y., Jia S., Bu N., Li N., Liu Y., Liu M., Yang Z., Liu S. (2021). Grain and Stoichiometry Engineering for Ultra-Sensitive Perovskite X-Ray Detectors. J. Mater. Chem. A.

[cit21] Ba Y., Han Y., Zhu W., Wang T., Chi J., Xi H., Zhao T., Chen D., Zhang J., Zhang C., Hao Y. (2024). Water-Assisted Mass Preparation of CsPbBr3-CsPb2Br5-CsPbIxBr3-x Composite Wafers for High-Performance X-Ray Detection. Chem. Eng. J..

[cit22] Li X., Chai Y., Juan Z., Wu Y., Liu Y. (2023). Suppressing the Ion Migration in Halide Perovskite Wafers for Current-Drift Free X ray Detectors. ACS Appl. Electron. Mater..

[cit23] Liu W., Shi T., Zhu J., Zhang Z., Li D., He X., Fan X., Meng L., Wang J., He R., Ge Y., Liu Y., Chu P. K., Yu X. F. (2023). PbI2-DMSO Assisted In Situ Growth of Perovskite Wafers for Sensitive Direct X-Ray Detection. Adv. Sci..

[cit24] Alghamdi S., Bennett S., Crean C., Ghosh J., Gibbard H., Moss R., Reiss J., Wolfe D., Sellin P. (2022). Polycrystalline Formamidinium Lead Bromide X-Ray Detectors. Appl. Sci..

[cit25] Hu M., Jia S., Liu Y., Cui J., Zhang Y., Su H., Cao S., Mo L., Chu D., Zhao G., Zhao K., Yang Z., Liu S. F. (2020). Large and Dense Organic-Inorganic Hybrid Perovskite CH_3_NH_3_PbI_3_ Wafer Fabricated by One-Step Reactive Direct Wafer Production with High X-Ray Sensitivity. ACS Appl. Mater. Interfaces.

[cit26] Qian W., Xu X., Wang J., Xu Y., Chen J., Ge Y., Chen J., Xiao S., Yang S. (2021). An Aerosol-Liquid-Solid Process for the General Synthesis of Halide Perovskite Thick Films for Direct-Conversion X-Ray Detectors. Matter.

[cit27] Lu Y., He D., Yuan X., Yan Q., Shu X., Hu Z., Zhang Z., Liu Z., Jiang Z., Xu R., Wang W., Ma Z., Chen T., Xu H., Xu F., Hong F., Song H. (2024). CsPbIBr2 Thick Film With (100)-Preferred Orientation Enables Highly Sensitive X-Ray Detector by a Spray-Coating Method via Dual-Additive-Assisted Solution Strategy under Atmospheric Environment. Adv. Funct. Mater..

[cit28] Qian W., Qiu W., Yu S., Huang D., Lei R., Huang X., Xiao S., Wang X., Yang S. (2023). Solvent Engineering of MAPbI3 Perovskite Thick Film for a Direct X-Ray Detector. Nanoscale.

[cit29] Glushkova A., Andričević P., Smajda R., Náfrádi B., Kollár M., Djokić V., Arakcheeva A., Forró L., Pugin R., Horváth E. (2021). Ultrasensitive 3D Aerosol-Jet-Printed Perovskite X-Ray Photodetector. ACS Nano.

[cit30] Jin P., Tang Y., Li D., Wang Y., Ran P., Zhou C., Yuan Y., Zhu W., Liu T., Liang K., Kuang C., Liu X., Zhu B., (Michael) Yang Y. (2023). Realizing Nearly-Zero Dark Current and Ultrahigh Signal-to-Noise Ratio Perovskite X-Ray Detector and Image Array by Dark-Current-Shunting Strategy. Nat. Commun..

[cit31] Chen S., Liu W., Xu M., Shi P., Zhu M. (2023). Electrospray Prepared Flexible CsPbBr3 Perovskite Film for Efficient X-Ray Detection. J. Mater. Chem. C.

[cit32] Liu Y., Gao C., Li D., Zhang X., Zhu J., Wu M., Liu W., Shi T., He X., Wang J., Huang H., Sheng Z., Liang D., Yu X. F., Zheng H., Sun X., Ge Y. (2024). Dynamic X-Ray Imaging with Screen-Printed Perovskite CMOS Array. Nat. Commun..

[cit33] Huang H., Zheng Y., Liu C., Zhang Z., Gao M., Wang J., Liu Y., Chu P. K., Yu X. F. (2023). Interfacial Engineering Enables Perovskite Heteroepitaxial Growth on Black Phosphorus for Flexible X-Ray Detectors. Small.

[cit34] Mescher H., Schackmar F., Eggers H., Abzieher T., Zuber M., Hamann E., Baumbach T., Richards B. S., Hernandez-Sosa G., Paetzold U. W., Lemmer U. (2020). Flexible Inkjet-Printed Triple Cation Perovskite X-Ray Detectors. ACS Appl. Mater. Interfaces.

[cit35] Demchyshyn S., Verdi M., Basiricò L., Ciavatti A., Hailegnaw B., Cavalcoli D., Scharber M. C., Sariciftci N. S., Kaltenbrunner M., Fraboni B. (2020). Designing Ultraflexible Perovskite X-Ray Detectors through Interface Engineering. Adv. Sci..

[cit36] Li W., Xu Y., Peng J., Li R., Song J., Huang H., Cui L., Lin Q. (2021). Evaporated Perovskite Thick Junctions for X-Ray Detection. ACS Appl. Mater. Interfaces.

[cit37] Verdi M., Giuri A., Ciavatti A., Rizzo A., Esposito Corcione C., Basiricò L., Colella S., Fraboni B. (2023). Record Stability for Fully Passive Perovskite-Based X-Ray Detectors Through the Use of Starch as Templating Agent. Adv. Mater. Interfaces.

[cit38] Kim Y. C., Kim K. H., Son D. Y., Jeong D. N., Seo J. Y., Choi Y. S., Han I. T., Lee S. Y., Park N. G. (2017). Printable Organometallic Perovskite Enables Large-Area, Low-Dose X-Ray Imaging. Nature.

[cit39] Yin L., Wu H., Pan W., Yang B., Li P., Luo J., Niu G., Tang J. (2019). Controlled Cooling for Synthesis of Cs2AgBiBr6 Single Crystals and Its Application for X-Ray Detection. Adv. Opt. Mater..

[cit40] Jagt R. A., Bravić I., Eyre L., Gałkowski K., Borowiec J., Dudipala K. R., Baranowski M., Dyksik M., van de Goor T. W. J., Kreouzis T., Xiao M., Bevan A., Płochocka P., Stranks S. D., Deschler F., Monserrat B., MacManus-Driscoll J. L., Hoye R. L. Z. (2023). Layered BiOI Single Crystals Capable of Detecting Low Dose Rates of X-Rays. Nat. Commun..

[cit41] Xia M., Yuan J. H., Niu G., Du X., Yin L., Pan W., Luo J., Li Z., Zhao H., Xue K. H., Miao X., Tang J. (2020). Unveiling the Structural Descriptor of A3B2X9 Perovskite Derivatives toward X-Ray Detectors with Low Detection Limit and High Stability. Adv. Funct. Mater..

[cit42] Zhang J., Li A., Li B., Yang M., Hao X., Wu L., Zhao D., Xia G., Ren Z., Tian W., Yang D., Zhang J. (2022). Top-Seed Solution-Based Growth of Perovskite Cs3Bi2I9Single Crystal for High Performance X-Ray Detection. ACS Photonics.

[cit43] Zhao Z., Fan Q., Liu Y., Rong H., Ni H., Wei L., Zhao X., Luo J., Sun Z. (2024). Lead-Free Bismuth-Based Perovskite X-Ray Detector with High Sensitivity and Low Detection Limit. ACS Appl. Mater. Interfaces.

[cit44] Gupta S., Sarisozen S., Kumar Khuntia S., Lang F., Mahadevan P., Bhattacharyya S. (2025). X-Ray Detection with High Dynamic Sensitivity
and Ultra-Low Detection Limits by Low-Dimensional Hybrid Bismuth-Iodides. Adv. Funct. Mater..

[cit45] Starkholm A., Al-Sabbagh D., Sarisozen S., von Reppert A., Rössle M., Ostermann M., Unger E., Emmerling F., Kloo L., Svensson P. H., Lang F., Maslyanchuk O. (2025). Green Fabrication of Sulfonium-Containing Bismuth Materials for High-Sensitivity X-Ray Detection. Adv. Mater..

[cit46] Daum M., Deumel S., Sytnyk M., Afify H. A., Hock R., Eigen A., Zhao B., Halik M., These A., Matt G. J., Brabec C. J., Tedde S. F., Heiss W. (2021). Self-Healing Cs_3_Bi_2_Br_3_I_6_ Perovskite Wafers for X-Ray Detection. Adv. Funct. Mater..

[cit47] Yang B., Pan W., Wu H., Niu G., Yuan J. H., Xue K. H., Yin L., Du X., Miao X. S., Yang X., Xie Q., Tang J. (2019). Heteroepitaxial Passivation of Cs_2_AgBiBr_6_ Wafers with Suppressed Ionic Migration for X-Ray Imaging. Nat. Commun..

[cit48] Tie S., Zhao W., Xin D., Zhang M., Long J., Chen Q., Zheng X., Zhu J., Zhang W. H. (2020). Robust Fabrication of Hybrid Lead-Free Perovskite Pellets for Stable X-Ray Detectors with Low Detection Limit. Adv. Mater..

[cit49] Li M., Li H., Li W., Li B., Lu T., Feng X., Guo C., Zhang H., Wei H., Yang B. (2022). Oriented 2D Perovskite Wafers for Anisotropic X-Ray Detection through a Fast Tableting Strategy. Adv. Mater..

[cit50] Bu N., Jia S., Xiao Y., Li H., Li N., Liu X., Yang Z., Zhao K., Liu S. (2022). Inch-Size Cs3Bi2I9 Polycrystalline Wafers with near-Intrinsic Properties for Ultralow-Detection-Limit X-Ray Detection. J. Mater. Chem. C.

[cit51] Jia S., Xiao Y., Bu N., Li N., Li D., Yang Z., Liu S. (2023). Sintered Polycrystalline BiVO4 Pellet for Stable X-Ray Detector with Low Detection Limit. Adv. Funct. Mater..

[cit52] Wang X., Wei W., Zhang S., Li Z., Wang J., Sun J., Chang Y., Yang Y., Meng X. (2025). Self-Powered Ferroelectric Bi-Based Perovskite X-Ray Detector for Radiation Monitoring and Intelligent Imaging Recognition. Laser Photonics Rev..

[cit53] Mao L., Li Y., Chen H., Yu L., Zhang J. (2021). A High-Sensitivity Flexible Direct X-Ray Detector Based on Bi2O3/Pdms Nanocomposite Thin Film. Nanomaterials.

[cit54] Karthieka R. R., Begum R. N., Prakash T. (2021). Direct Conversion X-Ray Sensing Nature of Bismuth (III) Iodide Thick Films. Chin. J. Phys..

[cit55] Dong S., Xin D., Zhang M., Tie S., Cai B., Ma Q., Zheng X. (2022). Green Solvent Blade-Coated MA3Bi2I9 for Direct-Conversion X-Ray Detectors. J. Mater. Chem. C.

[cit56] Kuo T.-T., Wu C.-M., Lu H.-H., Chan I., Wang K., Leou K.-C. (2014). Flexible X-Ray Imaging Detector Based on Direct Conversion in Amorphous Selenium. J. Vac. Sci. Technol., A.

[cit57] Zhao S., Wu Y., Jin Z., Zhao J., An K., Peng R., Wu X., Liang D., Qian Q., Mohammed O. F., Zang Z. (2025). Waterproof Scintillator for Efficient 3D X-Ray Imaging Enabled by Color and Space Reconfiguration. Matter.

[cit58] Zhao S., Jia Z., Huang Y., Qian Q., Lin Q., Zang Z. (2023). Solvent-Free Synthesis of Inorganic Rubidium Copper Halides for Efficient Wireless Light Communication and X-Ray Imaging. Adv. Funct. Mater..

[cit59] Li Y., Meng L., Yang Y., Xu G., Hong Z., Chen Q., You J., Li G., Yang Y., Li Y. (2016). High-Efficiency Robust Perovskite Solar Cells on Ultrathin Flexible Substrates. Nat. Commun..

[cit60] Bade S. G. R., Shan X., Hoang P. T., Li J., Geske T., Cai L., Pei Q., Wang C., Yu Z. (2017). Stretchable Light-Emitting Diodes with Organometal-Halide-Perovskite–Polymer Composite Emitters. Adv. Mater..

[cit61] Bade S. G. R., Li J., Shan X., Ling Y., Tian Y., Dilbeck T., Besara T., Geske T., Gao H., Ma B., Hanson K., Siegrist T., Xu C., Yu Z. (2016). Fully Printed Halide Perovskite Light-Emitting Diodes with Silver Nanowire Electrodes. ACS Nano.

[cit62] Li H., Wang C. F., Luo Q. F., Ma C., Zhang J., Zhao R., Yang T., Du Y., Chen X., Li T., Liu X., Song X., Yang Y., Yang Z., Liu S., Zhang Y., Zhao K. (2024). Wearable Photoferroelectric Perovskite X-Ray Detectors. Adv. Funct. Mater..

[cit63] Zhou Y., Zhao L., Ni Z., Xu S., Zhao J., Xiao X., Huang J. (2021). Heterojunction Structures for Reduced Noise in Large-Area and Sensitive Perovskite X-Ray Detectors. Sci. Adv..

[cit64] Liu X., Li H., Cui Q., Wang S., Ma C., Li N., Bu N., Yang T., Song X., Liu Y., Yang Z., Zhao K., Liu S. (2022). Molecular Doping of Flexible Lead-Free Perovskite-Polymer Thick Membranes for High-Performance X-Ray Detection. Angew. Chem., Int. Ed..

[cit65] Liu X., Cui Q., Li H., Wang S., Zhang Q., Huang W., Liu C., Cai W., Li T., Yang Z., Ma C., Ren L., Liu S. F., Zhao K. (2024). Biocompatible Metal-Free Perovskite Membranes for Wearable X-Ray Detectors. ACS Appl. Mater. Interfaces.

[cit66] Li H., Liu X., Yang T., Ma C., Du Y., Xu P., Zhang L., Song X., Cui Q., Zhao S., Yang Z., Liu S. F., Jin S., Zhao K. (2024). Flexible Large-Scale Self-Driven Perovskite X-Ray Detector by Precise Heterogeneous Integration. ACS Energy Lett..

[cit67] Liu C., Wu W., Zhang D., Li Z., Ren G., Han W., Guo W. (2021). Effective Stability Enhancement in ZnO-Based Perovskite Solar Cells by MACl Modification. J. Mater. Chem. A.

[cit68] Lehnen A. C., Gurke J., Bapolisi A. M., Reifarth M., Bekir M., Hartlieb M. (2022). Xanthate-Supported Photo-Iniferter (XPI)-RAFT Polymerization: Facile and Rapid Access to Complex Macromolecules. Chem. Sci..

[cit69] Li X., Sheng W., Duan X., Lin Z., Yang J., Tan L., Chen Y. (2022). Defect Passivation Effect of Chemical Groups on Perovskite Solar Cells. ACS Appl. Mater. Interfaces.

[cit70] Peng J., Khan J. I., Liu W., Ugur E., Duong T., Wu Y., Shen H., Wang K., Dang H., Aydin E., Yang X., Wan Y., Weber K. J., Catchpole K. R., Laquai F., De Wolf S., White T. P. (2018). A Universal Double-Side Passivation for High Open-Circuit Voltage in Perovskite Solar Cells: Role of Carbonyl Groups in Poly(Methyl Methacrylate). Adv. Energy Mater..

[cit71] Hong Y., Zhou H., Qian W., Zuo B., Wang X. (2017). Impact of the α-Methyl Group (α-CH_3_) on the Aggregation States and Interfacial Isotherms of Poly(Acrylates) Monolayers at the Water Surface. J. Phys. Chem. C.

[cit72] Zhou Z., Pang S., Ji F., Zhang B., Cui G. (2016). The Fabrication of Formamidinium Lead Iodide Perovskite Thin Films via Organic Cation Exchange. Chem. Commun..

[cit73] Hills-Kimball K., Nagaoka Y., Cao C., Chaykovsky E., Chen O. (2017). Synthesis of Formamidinium Lead Halide Perovskite Nanocrystals through Solid-Liquid-Solid Cation Exchange. J. Mater. Chem. C.

[cit74] Taylor V. C. A., Tiwari D., Duchi M., Donaldson P. M., Clark I. P., Fermin D. J., Oliver T. A. A. (2018). Investigating the Role of the Organic Cation in Formamidinium Lead Iodide Perovskite Using Ultrafast Spectroscopy. J. Phys. Chem. Lett..

[cit75] Yang Z., Zhang W., Wu S., Zhu H., Liu Z., Liu Z., Jiang Z., Chen R., Zhou J., Lu Q., Xiao Z., Shi L., Chen H., Ono L. K., Zhang S., Zhang Y., Qi Y., Han L., Chen W. (2021). Slot-Die Coating Large-Area Formamidinium-Cesium Perovskite Film for Efficient and Stable Parallel Solar Module. Sci. Adv..

[cit76] Zhu L., Xu S., Liu G., Liu L., Zhou H., Ai Z., Pan X., Zhang F. (2024). Engineering the Passivation Routes of Perovskite Films towards High Performance Solar Cells. Chem. Sci..

[cit77] Yuan S., Qian F., Yang S., Cai Y., Wang Q., Sun J., Liu Z., Liu S. (2019). NbF5: A Novel α-Phase Stabilizer for FA-Based Perovskite Solar Cells with High Efficiency. Adv. Funct. Mater..

[cit78] Askar A. M., Karmakar A., Bernard G. M., Ha M., Terskikh V. V., Wiltshire B. D., Patel S., Fleet J., Shankar K., Michaelis V. K. (2018). Composition-Tunable Formamidinium Lead Mixed Halide Perovskites via Solvent-Free Mechanochemical Synthesis: Decoding the Pb Environments Using Solid-State NMR Spectroscopy. J. Phys. Chem. Lett..

[cit79] Zhang Y., Fan H., Wang Y., Zuo B., Zhang W., Wang S., Wang X. (2015). Influence of the Linkage Type between the Polymer Backbone and Side Groups on the Surface Segregation of Methyl Groups during Film Formation. Soft Matter.

[cit80] Giuri A., Vanni N., Ahmad M., Rolston N., Esposito Corcione C., Listorti A., Colella S., Rizzo A. (2023). Incorporation of Functional Polymers into Metal Halide Perovskite Thin-Films: From Interactions in Solution to Crystallization. Mater. Adv..

[cit81] Giuri A., Vanni N., Mastria R., Carallo S., Colella S., Aiello F., Balzano F., Uccello-Barretta G., Ahmad M., Rolston N., Rizzo A., Esposito Corcione C. (2025). Crystallization Dynamics of Hybrid Perovskite Mediated by a Biopolymer: From Precursor Solution to Thin Film. Adv. Mater. Interfaces.

[cit82] Bi D., Yi C., Luo J., Décoppet J. D., Zhang F., Zakeeruddin S. M., Li X., Hagfeldt A., Grätzel M. (2016). Polymer-Templated Nucleation and Crystal Growth of Perovskite Films for Solar Cells with Efficiency Greater than 21%. Nat. Energy.

[cit83] Xu Y., Liu G., Hu J., Wang G., Chen M., Chen Y., Li M., Zhang H., Chen Y. (2022). In Situ Polymer Network in Perovskite Solar Cells Enabled Superior Moisture and Thermal Resistance. J. Phys. Chem. Lett..

[cit84] Wu Y., Feng J., Yang Z., Liu Y., Liu S. (2023). Halide Perovskite: A Promising Candidate for Next-Generation X-Ray Detectors. Adv. Sci..

[cit85] Jeon N. J., Cho J. M., Lee J.-K. (2022). Halide Perovskites for X ray Detection: The Future of Diagnostic Imaging. Prog. Med. Radiat. Phys..

[cit86] Zhao X., Wang S., Song Y., Aoki T., Gnatyuk V., You L., Deng Z., Tao R., Fang X., Meng G. (2024). Freezing Non-Radiative Recombination in High-Performance CsPbBr3 Single Crystal X-Ray Detector. Appl. Phys. Lett..

[cit87] Sakhatskyi K., Turedi B., Matt G. J., Wu E., Sakhatska A., Bartosh V., Lintangpradipto M. N., Naphade R., Shorubalko I., Mohammed O. F., Yakunin S., Bakr O. M., Kovalenko M. V. (2023). Stable Perovskite Single-Crystal X-Ray Imaging Detectors with Single-Photon Sensitivity. Nat. Photonics.

[cit88] Domanski K., Correa-Baena J. P., Mine N., Nazeeruddin M. K., Abate A., Saliba M., Tress W., Hagfeldt A., Grätzel M. (2016). Not All That Glitters Is Gold: Metal-Migration-Induced Degradation in Perovskite Solar Cells. ACS Nano.

[cit89] Futscher M. H., Lee J. M., McGovern L., Muscarella L. A., Wang T., Haider M. I., Fakharuddin A., Schmidt-Mende L., Ehrler B. (2019). Quantification of Ion Migration in CH3NH3PbI3 Perovskite Solar Cells by Transient Capacitance Measurements. Mater. Horiz..

[cit90] Hui Y., Tan Y. Y., Chen L., Nan Z. A., Zhou J. Z., Yan J. W., Mao B. W. (2021). Stability of Perovskite Thin Films under Working Condition: Bias-Dependent Degradation and Grain Boundary Effects. Adv. Funct. Mater..

[cit91] Konstantatos G., Howard I., Fischer A., Hoogland S., Clifford J., Klem E., Levina L., Sargent E. H. (2006). Ultrasensitive Solution-Cast Quantum Dot Photodetectors. Nature.

[cit92] Lu X., Xin D., Lei L., Fan Z., Dong S., Tie S., Yuan R., Lin P., Zhu J., Zheng X. (2024). High-Performance Flat-Panel Perovskite X-Ray Detectors Enabled by Defect Passivation in Ruddlesden-Popper Perovskites. ACS Appl. Mater. Interfaces.

[cit93] Thompson M., Ellison S. L. R., Wood R. (2002). Resulting from the Symposium on Harmonization of Quality Assurance Systems for Analytical Laboratories.. Pure Appl. Chem..

[cit94] Lee C. H., Chien A. T., Yen M. H., Lin K. F. (2008). Poly(Methyl Acrylate-Co-Methyl Methacrylate)/Montmorillonite Nanocomposites Fabricated by Soap-Free Emulsion Polymerization. J. Polym. Res..

[cit95] Tu C. W., Liu K. Y., Chien A. T., Lee C. H., Ho K. C., Lin K. F. (2008). Performance of Gelled-Type Dye-Sensitized Solar Cells Associated with Glass Transition Temperature of the Gelatinizing Polymers. Eur. Polym. J..

[cit96] Khan S. A., Ali W., Wang M., Ouyang Z., Li G. (2025). Critical Roles of Polymers in the Development of Flexible Perovskite Solar Cells. Macromol. Rapid Commun..

[cit97] Rössle M., Leitenberger W., Reinhardt M., Koç A., Pudell J., Kwamen C., Bargheer M. (2021). The Time-Resolved Hard X-Ray Diffraction Endstation KMC-3 XPP at BESSY II. J. Synchrotron Radiat..

