# Supplementary material for: Highly sensitive X-ray detectors with polymer-perovskite-embedded flexible teflon membranes
Source: Mater Horiz. 2026 May 28;13(14):7128–42. doi: 10.1039/d5mh02084k (PMC13218327; doi:10.1039/d5mh02084k)
Supplement: MH-013-D5MH02084K-s001 [file MH-013-D5MH02084K-s001.pdf]

Supplementary Information (SI) for Materials Horizons.
This journal is © The Royal Society of Chemistry 2026

Supplementary Material to

Highly Sensitive X-ray Detectors with Polymer-Perovskite-Embedded Flexible Teflon Membranes

Sema Sarisozen[a], Anne-Catherine Lehnen[bc], Fan Hu[d], Gonul Ofkeli[ae], Alexander von Reppert[a], Matthias Rössle[f], Sercan Ozen[a], Seydanur Kaya[ag], Lucas Holte[a], Olena Maslyanchuk[h], Pedro B. Groszewicz[di], Matthias Hartlieb[bc], Dieter Neher[a], Felix Lang[a] *

[a]Institute of Physics and Astronomy, University of Potsdam, Karl-Liebknecht-Straße 24-25, 14476 Potsdam, Germany

[b]Institute of Chemistry, University of Potsdam, Karl-Liebknecht-Straße 24-25, Potsdam, 14476 Germany

[c]Fraunhofer Institute for Applied Polymer Research (IAP), Geiselbergstraße 69, Potsdam, Germany

[d]SE-ASPIN, Helmholtz-Zentrum Berlin für Materialien und Energie (HZB), 12489 Berlin, Germany

[e]Department of Chemistry, Izmir Institute of Technology, 35430 Izmir, Turkey

[f]Helmholtz-Zentrum Berlin für Materialien und Energie GmbH, Wilhelm-Conrad-Röntgen Campus, BESSY II, 12489 Berlin, Germany

[g]Central Research Laboratory, Kastamonu University, 37100, Kastamonu, Turkey

[h]Department for Solution-Processing of Hybrid Materials and Devices, Helmholtz-Zentrum Berlin, Berlin, Germany

[i]Department of Radiation Science and Technology, Delft University of Technology, Delft 2629JB, Netherlands

* Corresponding Author: Felix Lang; Email: felix.lang.1@uni-potsdam.de

Supplementary Materials and Methods:

Materials: Cesium iodide (CsI), formamidinium iodide (FAI) and methylammonium bromide (MABr), lead iodide (PbI_2), methylammonium chloride (MACl) and bathocuproine (BCP, purity of 99.9%), C_{60}, bathocuproine (BCP), and chromium (Cr) were purchased from Sigma-Aldrich. The solvents, including dimethyl formamide (DMF), dimethyl sulfoxide (DMSO), isopropanol (IPA) and chlorobenzene (CB) were purchased from Sigma Aldrich. PTFE hydrophilic membrane was purchased from Amazon, supplied by Labfil (Part of ALWSCI, REF C0002033), LOT L04488MF).

Preparation of the Teflon (PTFE) membranes: The membranes were immersed in ethanol for 10 minutes to remove surface contaminants. Following the ethanol treatment, they were dried in a vacuum oven at 40°C for 30 minutes to eliminate any residual ethanol and moisture completely.

Preparation of the triple cation perovskite (TCP)solution: The perovskite solution was prepared according to the approach published by Li et al (2022).[1] Typically, 1.73M perovskite solution was prepared by dissolving MABr (3.68 mg), MACl (18.105 mg), CsI (22.47 mg), FAI (276.98 mg), and PbI_2 (909.19 mg) were dissolved in a 5:1 DMF/DMSO in solvent mixture, consisting of 833.33 µL of DMF and 166.66 µL of DMSO, and stirred at 55°C for 3 hours. Before use, the solution was filtered through a Teflon filter.

Preparation of the PEM: 80µL of triple-cation perovskite solution was spin-coated onto the membranes in two steps; first 10s at 1000 rpm, then 40s at 5000 rpm. Twelve seconds before the spin coating ended, 250 µL of chlorobenzene (CB) slowly dripped onto the center of the membranes. The PEMs were then annealed in a preheated oven at 110 °C for 20 min.

Synthesis of Poly(methyl acrylate) PMA: Methyl acrylate (3 mmol, 258 mg, 275 µL), 2-(((butylthio)carbonothioyl)thio)propanoic acid (0.0027 mmol, 0.64 mg), 2 ((ethoxycarbonothioyl)thio)-2-methylpropanoic acid (0.0003 mmol, 0.1 mg) and 1,4-dioxane (2728 µL) were added to a reaction vial (4 mL) equipped with a rubber septum. Oxygen was removed by degassing the solution for 10 min using nitrogen. Then the reaction mixture was irradiated for 3 h at 365 nm (44 W). Solvent and residuals were removed via precipitation in cold diethyl ether.

Preparation of the PMA-PEM: 5mg of PMA was dissolved in 1ml of 5:1 DMF/DMSO mixture. Afterwards, PMA containing solution was added to perovskite powder and stirred at 55°C for 3 hours Then**,** 80µL of the mixture was spin coated onto the membranes in two steps: first 10s at 1000 rpm, then 40s at 5000 rpm. Twelve seconds before the end of spin coating, 250 µL of chlorobenzene (CB) slowly dripped onto the center of the membranes. The PEMs were then annealed in a preheated oven at 110 °C for 20 min.

Solution-State Nuclear Magnetic Resonance: ^1H-NMR spectra were recorded on a Bruker AVANCE NEO 400 MHz spectrometer using $CDCl_3$ as solvent. The residual proton signal of the solvent was used as an internal standard and chemical shift values were reported in ppm.

Size Exclusion Chromatography (SEC): Measurements were recorded with parallel RI and UV detection, and the stationary phase was a 300 × 8 mm^2 PSS SDV linear M column at room temperature and at a flow rate of 0.5 mL min^{-1}. Tetrahydrofuran (THF) served as eluent, and a polystyrene (PS) calibration was used.

Solid State-Nuclear Magnetic Resonance (SS-NMR): Solid-state NMR spectra were recorded with a 400 MHz Ascend NEO spectrometer using a 1.9mm HX MAS probe. Rotors were centre packed, closed with Vespel caps and spun to an MAS frequency of 40 kHz. ^{1}H MAS NMR spectra with direct excitation were collected using a 90-180-180 pulse sequence for background suppression. A total of 64 scans were collected using an RF-field strength of 153 kHz and a recycle delay of 300 s. ^{207}Pb MAS NMR spectra were acquired at 83.84 MHz using a rotor-synchronized Hahn-echo sequence. Pulses were set to an RF-field strength of ~96 kHz to ensure that excitation width encompasses the spectral region of interest. A total of 32768 scans were accumulated with a recycle delay of 0.5 s. ^{207}Pb->^{1}H cross polarization experiments were optimized to a Hartmann-Hahn condition under MAS with RF-field strengths of 96kHz for ^{1}H and 60 kHz for ^{207}Pb, whereas a 90° pulse of 2.6 μs was employed for ^{207}Pb excitation. An 80-100 ramp during the 5 ms long contact time, with recycle delay of 0.5 s and a number of scans equal to 32768. Chemical shift scale of ^{1}H and ^{207}Pb were calibrated using adamantane (+1.8 ppm) and lead nitrate (-3494 ppm) as secondary references, respectively.

Fourier Transform-Infrared Spectroscopy (FT-IR): FTIR spectra were recorded in extinction mode using a Nicolet iS10 FTIR spectrometer (Thermo Fisher Scientific) equipped with a Smart OMNI-Transmission accessory. Data collection and processing were performed via OMNIC software, where the y-axis was set to extinction to accurately account for both absorption and scattering effects within the film samples.

X-Ray Transmission Images: X-ray transmission images were captured by placing the perovskite films and a blank membrane between the X-ray source and the detector. A 4 mm mask and a Ni filter were used to prevent detector saturation. The measurements were performed using an X-ray diffractometer (Empyrean Series 3, Malvern Panalytical) operated at a current of 5 mA and a voltage of 15 kV, and image collection was performed using a 3D PIXel detector (Medipix3 collaboration).

Photoluminescence (PL) Imaging: PL imaging was performed using a fluorescence microscope (Olympus BX51) with a halogen lamp (Olympus U-RFL-T-200) as the excitation source. Samples were placed on a custom-designed holder and exposed to blue light in the 400–440 nm range. The emitted photoluminescence from the perovskite was collected using a CCD camera (Hamamatsu Photonics C4742-80-12AG) equipped with a long-pass filter (> 475 nm) to block the excitation light.

Scanning Electron Microscope (SEM): Scanning electron microscopy (SEM) imaging was performed using an FEI Quanta FEG 250 equipped with an Everhart-Thornley Detector (ETD) under high vacuum conditions. The measurements were conducted at an accelerating voltage of 10 kV.

Grazing-Incidence Wide-Angle X-ray Scattering (GIWAXS): Grazing-incidence wide-angle X-ray scattering (GIWAXS) measurements were performed using the Empyrean Series 3 diffractometer with Cu anode X-ray source (primary K_α emission line), operating at 40 kV and 40 mA. The incident X-ray beam was directed onto the sample via a collimating mirror, and the scattered X-rays were collected using a 3D PIXcel detector (256 * 256 pixels of 55 μm pitch). To investigate the crystallographic orientation and molecular packing of the films, measurements were conducted at 2θ angle of 16°, with an incident angle (ω) of 3° and an ω offset of −5°. Structural investigation in reciprocal space was facilitated by mapping the raw GIWAXS data to q-space using PyGIX based azimuthal angle integration. This process

followed a rigorous geometric calibration of the detector's distance and spatial orientation (tilts), conducted with a Cr_2O_3 reference through the PyFAI library.

X-ray Diffraction (XRD): X-ray diffraction (XRD) patterns were acquired using an Empyrean Series 3 X-ray diffractometer (Malvern Panalytical) in Bragg-Brentano geometry with Cu anode X-ray source (primary K_α emission line), operating at 40 kV and 40 mA. Data collection was performed using a 3D PIXel detector (Medipix3 collaboration), ensuring high-resolution diffraction analysis.

Absolute Photoluminescence Measurements: Photoluminescence (PL) measurements were performed using a 520 nm continuous-wave (CW) laser coupled with an optical fiber into an integrating sphere. The laser intensity was adjusted to match the equivalent of 1 sun intensity. A second optical fiber connected the output of the integrating sphere to an Andor SR393iB spectrometer equipped with a silicon CCD camera (DU420A-BR-DD, iDus). The system was calibrated with a halogen lamp of known spectral irradiance, which was directed into the integrating sphere. A spectral correction factor was established to align the detector's spectral response with the lamp's spectral irradiance. The spectral photon density was calculated from the corrected detector signal (spectral irradiance) by dividing by the photon energy (*hf*), and the photon counts for both excitation and emission were determined through numerical integration using a Matlab script.

Preparation of the X-ray detectors: A thermal evaporator was used to fabricate a complete device from free-standing perovskite membranes. Using a 6-pixel mask, 30 nm C_{60} at 0.2 Å/s, 8 nm BCP at 0.2 Å/s and 20 nm Cr at 0.1 Å/s were evaporated on one side of the membrane. On the opposite side, only 100 nm Cr was deposited at 0.1 Å/s using a u-shaped mask. All layers were evaporated under 10^{-7} hPa vacuum.

X-Ray Response Measurements Under Continuous Illumination: The X-ray response curves were obtained using a Cu anode X-ray source with primary K_α emission line, integrated into an X-ray diffractometer (Empyrean Series 3, Malvern Panalytical). The X-ray tube operated at an acceleration voltage of 40 kV and a current of 40 mA, positioned perpendicularly to the perovskite embedded membranes PEMs at 22 cm. The X-ray dose rates were modulated by placing a series of filters between the source and the sample. The filters used for attenuation were 0.1 mm Cu, 0.2 mm Cu, 0.3 mm Cu, and 0.4 mm Cu combined with 0.02 mm Ni. The dose rates were calibrated using the dosimeter (STEP OD-01, Pockau, Germany). The response of the PEMs to X-rays were measured directly with a Keithley 2400 Source Meter. Additionally, a Keithley 617 programmable electrometer was utilized for 0 V measurements, and a Keithley 237 was used for measurements exceeding 210 V.

X-ray Response Measurements Under Pulsed Illumination at the KMC-3 XPP Beamline at BESSY II: X-ray measurements were performed at the KMC-3 XPP beamline of the BESSY II synchrotron (Helmholtz-Zentrum Berlin) using photon energies from 7 keV to 15 keV. The photon flux was calibrated using a Hybrid Photon Counting detector (DECTRIS PILATUS 100k) and adjusted by the combination of aluminum absorbers with thicknesses ranging from 50 to 900 μm. The experiment was conducted under BESSY's standard operational mode, commonly referred to as the multibunch mode, in which X-ray timing is dictated by the charge distribution of short electron bunches circulating in the storage ring at 2 ns (60 cm distance) intervals, with bunch lengths varying between 45 ps and 80 ps (rms).[2] Despite these operational conditions, the detectors under investigation exhibited a stable and reliable response. A

Keithley 2400 Source Meter was used in both measurement setups to apply the bias voltage and record the photocurrent. All characterizations were carried out at room temperature and under ambient air conditions, with optical and electrical shielding in place to mitigate the effects of electromagnetic interference and ambient light.

Dose Rate Calculations for the Continuous Illumination Measurements: The STEP OD-01 dosimeter (Pockau, Germany) has been used to calculate the dose rate, *D*. The ionizing radiation dose in air, D_{air}, was evaluated as

$$D_{\mathrm{air}} = \frac{D \cdot A}{k \cdot T_{07}} \cdot \frac{\left(\frac{\mu}{\rho}\right)_{\mathrm{air}}}{\left(\frac{\mu}{\rho}\right)_{\mathrm{tissue}}} \approx D \cdot A \cdot 1.286 \left(\frac{\mathrm{Gy}}{\mathrm{s}}\right) \tag{1}$$

where A is the contact area of the detector, $(\mu/\rho)_{\mathrm{air}}$ and $(\mu/\rho)_{\mathrm{tissue}}$ are the mass attenuation coefficients of 8 keV photons in air and soft tissue, respectively. k represents the energy-dependent response of the dosimeter at 8 keV and T_{07} denotes the transmittance of photons through 0.07 mm of soft tissue.[3,4]

X-Ray Sensitivity: The X-ray sensitivity (S) is defined as the signal current density per unit of radiation exposure, and is calculated as follows:[4]

$$S = \frac{\bar{I}_{photo} - \bar{I}_{dark}}{A \cdot D_{air}} = \frac{I_{signal}}{A \cdot D_{air}} \tag{2}$$

with A being the electrode area and D_{air} is the dose rate of the X-Rays.

Signal-to-Noise (SNR) Ratio: The signal-to-noise ratio (SNR) is defined as the ratio of the signal current (I_{signal}) to the noise current (I_{noise}). The signal current is determined by subtracting the average dark current ($\bar{I}_{dark}$) from the average photocurrent ($\bar{I}_{photo}$). The noise current is calculated as the standard deviation of the photocurrent.[5–7]

$$SNR = \frac{I_{signal}}{I_{noise}} = \frac{\bar{I}_{photo} - \bar{I}_{dark}}{\sqrt{\frac{1}{N}\sum_i^N \left(I_i - \bar{I}_{photo}\right)^2}} \tag{3}$$

Detection Limit (LoD): The International Union of Pure and Applied Chemistry (IUPAC) defined the dose rate with a signal to noise ratio (SNR) value of 3 as the detection limit at a specified electric field.[8]

Supplementary Data

Table S1. Comparison of the sensitivities and limits of detection (LoDs) of Pb based, non-Pb-based and commercial X-ray detectors across different operating biases, including those above 0.1 V bias.

Materials	Sensitivity (μC Gy_{air}^{-1} cm^{-2})	Limit of detection (LoD) (nGy_{air} s^{-1})	Flexibility	Fabrication method	Ref.
PEA_2PbBr_4 (SC)	2.99× 10^3	0.79	No	SC growth	9
$CsPbBr_3$ (SC)	8× 10^3	0.02	No	SC growth	10
MAPbI3 (SC)	7× 10^5	1.5	No	SC growth	11
$MAPbBr_{3-x}Cl_x$ (SC)	8.4× 10^4	7.6	No	SC growth	12
$FA_{0.85}MA_{0.1}Cs_{0.05}$ $PbI_{2.55}Br_{0.45}$ (SC)	(3.5 ± 0.2) × 10^6	42 nGy s^{-1}	No	SC growth	13
$MAPbI_3$ (Pellet)	4.2× 10^5	350	No	Solvent evaporation+ Hot Pressing+ Annealing	14
$CsPbBr_3$-$CsPb_2Br_5$-$CsPbI_xBr_{3-x}$ (Wafer)	2× 10^4	127,7 nGy s^{-1}	No	Coprecipitation+ pressing+ spray coating+ annealing	15
$MAGeI_3$ (SC)	4.2× 10^4 μC Gy^{-1} cm^{-2}	4.1 nGy s^{-1}	No	SC growth	16
$CsPbBr_3$ (Pellet)	1.44× 10^4	564	No	Ball milling+ pressing+ annealing	17
$MA_3Bi_2I_9$ (SC)	1.06× 10^4	0.62	No	SC growth	18
$FAPbI_3$ (SC)	4.15 × 10^5	1.1	No	SC growth	19
$GAMAPbI_3$ (SC)	2.3×10^4	16.9	No	SC growth	20

$FA_{0.96}$ $DMA_{0.04}$ PbI_3 (SC)	2.24×10^5	1.57	No	SC growth	21
$MAPbBr_3$ (wafer)	1.14×10^5	149	No	Low temperature hot pressing	22
$BiVO_4$ (Pellet)	241.3	62	No	Pressing+ sinter-ing	23
$MA_3Bi_2I_9$ (Pellet)	563	9.3	No	Cold isostatic pressing	24
$Cs_3Cu_2I_5$ (Wafer)	3.4×10^3	33.17	No	Hot pressing	25
$(DPA)_2BiI_9$ (0D SC)	2×10^4	0.98	No	SC growth	26
$MAPbBr_3$ (SC)	2×10^5	0.85	No	SC growth	27
$Cs_3Bi_2I_9$ (SC)	4.38×10^3	7.93	No	SC growth	28
Cd(Zn)Te	318	5×10^4	No		29
α-Se	20	5.5×10^3	No		30
HgI_2	1.6×10^3	1×10^4	No		31
Si	8	$<8.3\times 10^6$	No		29
$MAPbI_3$ (SC)	5.2×10^6	0.1	No	SC growth	32
CsFAGA:Sr (SC)	$(2.6 \pm 0.1) \times 10^4$ $(2.5 \pm 0.2) \times 10^6$	7.09 N/A	No	SC growth	33
Cs_2TeI_6 (Film)	76.27	170	Yes	Electrospraying	34
$CsPbBr_3$ (Film)	5.57×10^4	215	No	Hot pressing	35

$CsPbI_2Br$ (Film)	1.48×10^5	280	No	Aerosol-liquid-solid processing	36
$CsPbIBr_2$ (Film)	1.4×10^3	3.3×10^3	No	Pneumatic spraying	37
$MA_3Bi_2I_9$ (Film)	2.07×10^3	2.71	Yes	Membrane filling	38
$CsAg_2I_3$ (SC Microbelts)	515.49	1.46×10^4	Yes	Vapor assisted growth	39
$MAPbI_3$ (Film)	7.3×10^3	154	No	Aerosol-liquid-solid processing	40
DABCO-N_2H_5-I_3 (Film)	$(6.20\pm0.27)\times 10^3$	102.3	Yes	Membrane Filling	41
MAPbI3 (Film)	2.2×10^8	120 nGy s^{-1}	No	Aerosol-Jet-Printed	42
$(BA_2PbBr_4)_{0.5}FAPbI_3$ (Film)	1.36×10^4	4.2	No	Spin Coating	43
$FA_{0.92}Cs_{0.04}MA_{0.04}PbI_3$ (Film)	1.3×10^4	7.84	No	Spin Coating	44
$MA_{0.42}FA_{0.58}PbI_3$ (Quasi-monocrystalline Film)	1.16×10^6	37.4	No	Ultrasound-assisted crystallization+ hot pressing	45
MDABCO-NH_4I_3 (Film)	$(6.52\pm0.7)\times10^3$	77	Yes	Membrane filling	46
$BA_2MA_9Pb_{10}I_{31}$ (Quasi-2D perovskite Film)	2.97×10^4	20.9	No	Scraper-based coating	47
CsPbBr3 (Film)	823,12	14.61	Yes	Electrospraying	48
$CsPbBr_3$ (Film)	1.6×10^4	321	No	Screen-printing	49
PEA_2PbBr_4 (Micro-crystalline film)	806±6 μC Gy^{-1} cm^{-2}	42±4 nGy s^{-1}	Yes	Spin coating	50

$MAPbBr_3$ (Film)	(1.61±0.12) ×10^3 μC Gy^{-1} cm^{-2}	3×10^3 nGy s^{-1}	Yes	Electrochemical intercalation+ activation+ deposition+ epitaxial growth	51
$CsBi_3I_{10}$ (Film)	1.62×10^3 (on glass) 911.70 (on PET)	220 (on glass) 1130 (on PET)	No Yes	Spin coating	52
$[(CH_3CH_2)_3S]_6BiI_{30}$ (Pellet)	1.41×10^4	90	No	mechanosynthesis and isostatic compression	4
$[(CH_3CH_2)_3S]AgBiI_5$ (Pellet)	1.519×10^4	78	No	mechanosynthesis and isostatic compression	4
$Cs_{0.05}(MA_{0.02}FA_{0.98})_{0.95}Pb(I_{0.98}Br_{0.02}Cl_x)_3$ (Film)	0.9 × 10^5 @100V	0.46	Yes	Membrane filling& Spin coating	This work
$Cs_{0.05}(MA_{0.02}FA_{0.98})_{0.95}Pb(I_{0.98}Br_{0.02}Cl_x)_3$ -PMA (Film)	2.3 × 10^5 @100V	0.09	Yes	Membrane filling& Spin coating	This work

Note: Not all relevant studies are included in this table. The availability of both sensitivity and limit of detection (LoD) values was the focus, as these parameters are plotted in the manuscript. While an effort was made to include relevant literature, some studies may not be listed.

Table S2. Comparison of the sensitivities of Pb-based and non-Pb-based X-ray detectors operating at or below bias voltage of 0.1 V.

Materials	Sensitivity ($\mu C/Gy_{air}.cm^2$)	Operating Voltage(V)	Flexibility	Fabrication method	Ref.
$Cs_{0.1}(FA_{0.83}MA_{0.17})_{0.9}Pb(Br_{0.17}I_{0.83})_3$ (Film)	59.9	0.1	Yes	Inkjet printing	53
$CsPbBr_3$ (QD films)	17. $\mu C\ Gy^{-1}\ cm^{-2}$ (on PET)	0.1	Yes	Inkjet printing	54
$[NH_3(CH_2)_4NH_3]BiI_5$ films	394 ± 67	0	Yes	Membrane filling	55
$Cs_{0.05}(FA_{0.83}MA_{0.17})_{0.95}PbI_{3-x}Br_x$ films	9.3 ± 0.5	0	Yes	Spin coating	56
2D/3D ($MAPbI_3$) perovskite bilayer heterojunction films	292	0	Yes	Membrane filling	57
$FAPbI_3$ films	122.5	0	Yes	Thermal co-evaporation	58
$Cs_{0.05}FA_{0.79}MA_{0.16}Pb(I_{0.8}Br_{0.2})_3$ films	3.7 ± 0.1	0	No	Spin coating	59
$FAPbBr_3/NiO_x$ heterojunction (SC)	402	0	No	SC growth	60
$FA_{0.55}MA_{0.45}PbI_3$ (SC)	8.7×10^4	0	No	SC growth	61
$MAPbI_3$-PDI-EAI (Film)	$(1.43±0.01)\times10^5$ (on ITO) $(1.03±0.02)\times10^5$ (on PET)	0	No Yes	Spin coating	62
$Cs_{0.05}(MA_{0.02}FA_{0.98})_{0.95}Pb(I_{0.98}Br_{0.02}Cl_x)_3$ (Film)	2.2×10^3	0	Yes	Membrane filling& Spin coating	This work

$Cs_{0.05}(MA_{0.02}FA_{0.98})_{0.95}Pb(I_{0.98}Br_{0.02}Cl_x)_3$ /PMA (Film)	4.2×10^3	0	Yes	Membrane filling& Spin coating	This work

Note: This table summarizes Pb-based and non-Pb-based X-ray detectors operating below 0.1V bias. While an effort was made to include relevant literature, some studies may not be listed.

Table S3. Copper and Nickel absorber thicknesses and their corresponding dose rates in the X-ray setup.

No	Absorber thickness	Dose Rate, (D_{air}) $\mu Gy_{air}\cdot s^{-1}$
1	0	184.66325
2	0.1mm Copper	4.51061
3	0.2mm Copper	0.81258
4	0.3mm Copper	0.26527
5	0.4mm Copper +0.02mm Nickel	0.09391

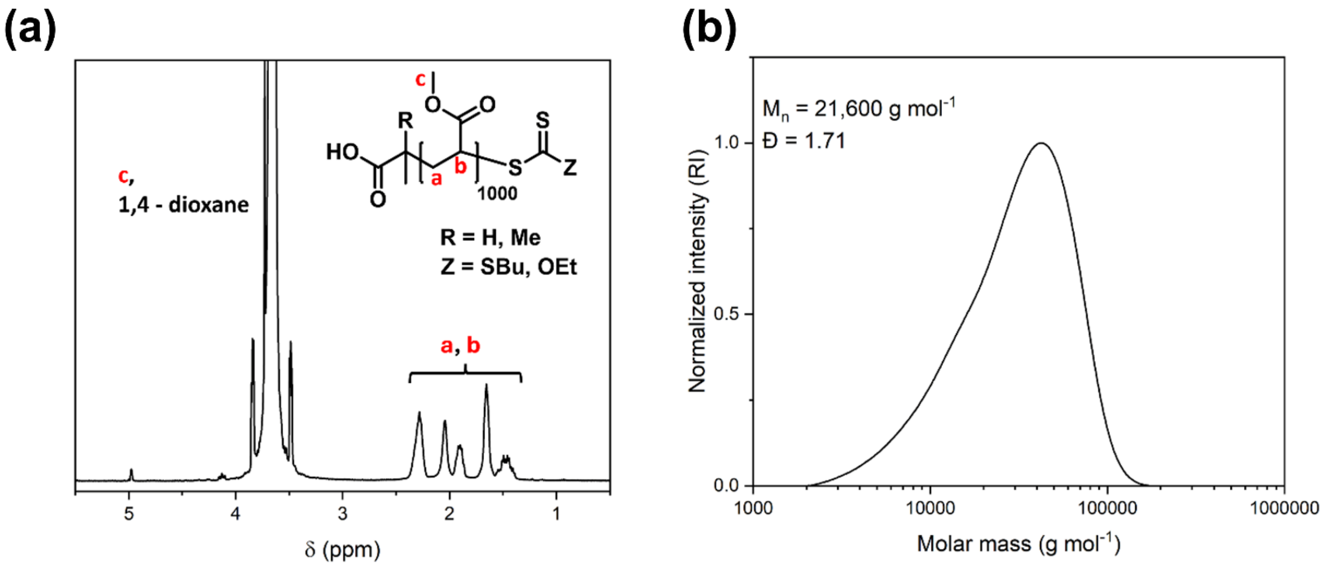

Figure S1. ^{1}H-NMR spectra of the reaction mixture after polymerization measured in deuterated chloroform ($CDCl_3$) (b) SEC curve of purified PMA. Eluent was tetrahydrofuran (THF), and a polystyrene (PS) calibration was used.

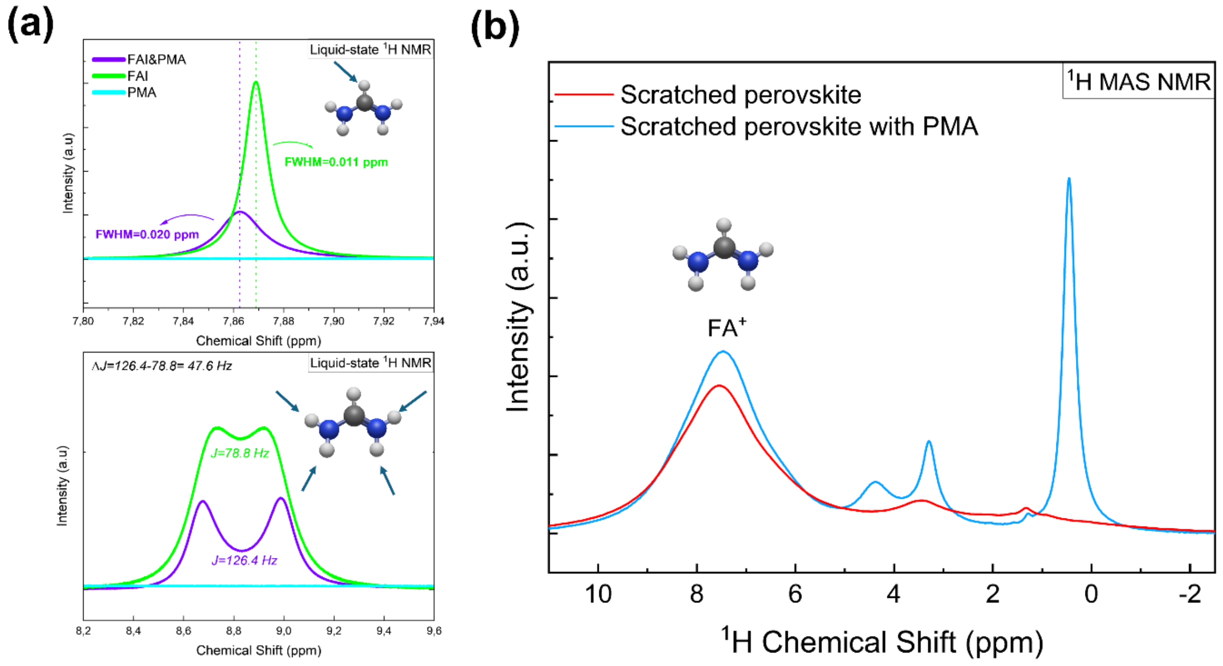

Figure S2. NMR investigation of the PMA-FA^+ interaction in solution and solid-state. (a) Solution-state ^{1}H NMR spectra of FAI with and without PMA in DMSO-d_6. The addition of PMA causes a broadening of the FA^+ -CH peak (~7.9 ppm, top) and a substantial increase in the J-coupling constant of the -NH_2 proton doublet (~8.8 ppm, bottom). (b) Solid-state ^{1}H MAS NMR spectra of the final perovskite films. The change in the line shape of the main FA^+ peak at ~7.5 ppm in the scratched perovskite with PMA sample (blue) compared to the scratched perovskite (red) indicates that this interaction iis maintained in the solid state, influencing the local environment of the organic cations.

In Figure S2b, the spectra of the all scratched perovskites confirms the presence of the expected FA^+ and MA^+ cations, with characteristic signals centered at 7.5 ppm and 3.5 ppm, respectively, which is in excellent agreement with the assignments reported for similar triple-cation perovskites.[63] The minor peak at high field is attributed to remaining solvent molecules from the spin coating procedure. The spectrum of the scratched perovskite with PMA exhibits these same perovskite signals in addition to a new, sharp resonance at 0.5 ppm, which is assigned to the PMA methyl groups and confirms its successful incorporation into the final film.

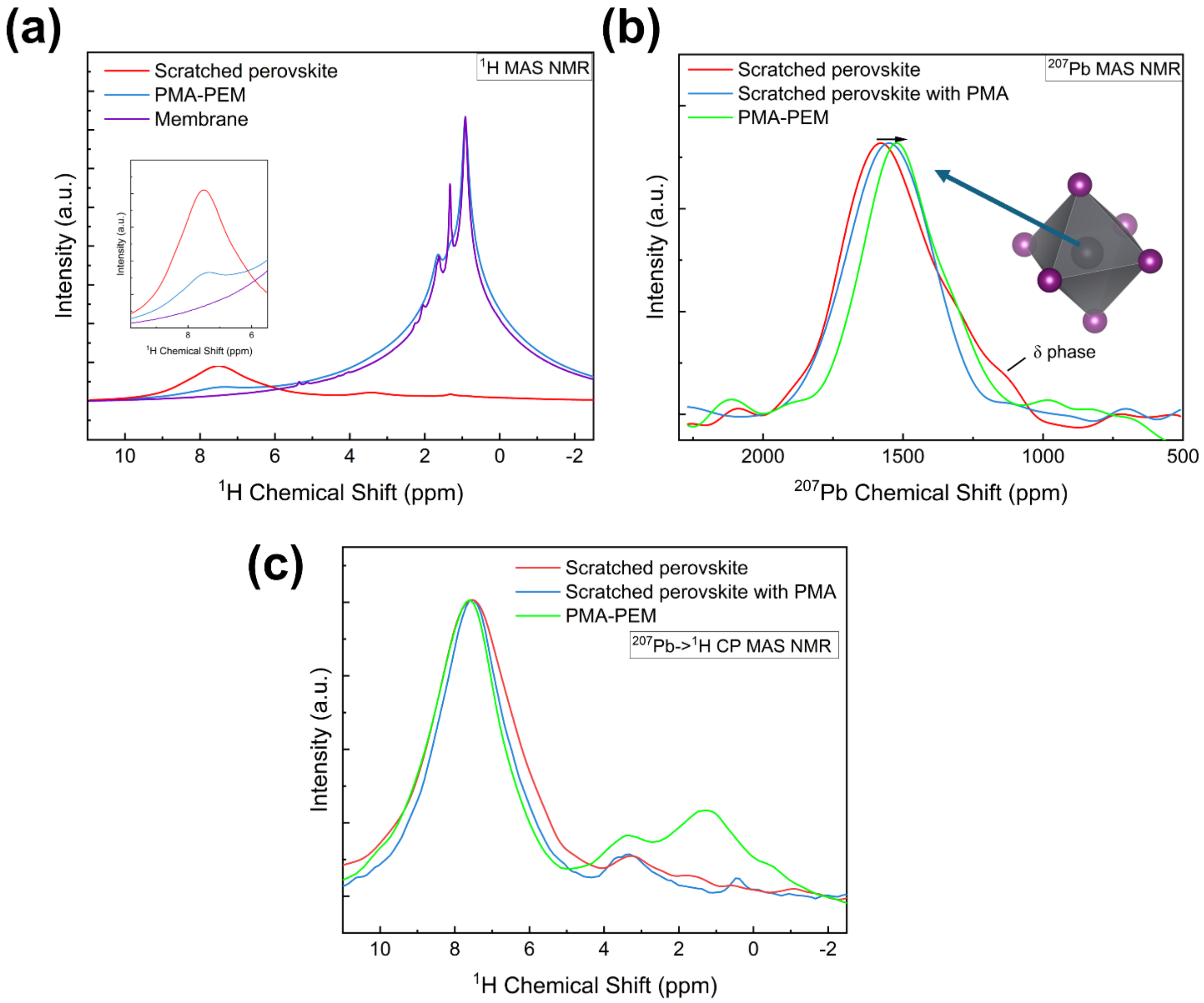

Figure S3. Solid-state NMR analysis of all samples. (a) ^{1}H MAS NMR spectra confirming the composition of the scratched perovskite, PMA-PEM, and the pure membrane. (b) ^{207}Pb NMR spectra revealing that PMA enhances phase stability by suppressing the δ-phase degradation (~1200 ppm). Furthermore, a chemical shift of the main resonance is observed for both PMA-containing samples (blue and green lines) compared to the scratched perovskite without PMA (red line), with the shift being most pronounced for the final PMA-PEM device, confirming a direct interaction with the Pb^{2+} lattice. (c) ^{207}Pb->^{1}H CP MAS NMR spectra of all samples.

Supporting Figure S3a compares the ^{1}H MAS NMR spectra of the membrane (from PTFE supported on polypropylene (PP)) to PMA-PEM. The broad baseline of these spectra is due to the only partial averaging of strong homonuclear dipolar couplings within the PP fibers. Such a broad baseline is absent for the scratched perovskites with PMA sample. This suggests a higher mobility of polymer chains. Whereas all three signals expected from the polypropylene monomer are observed in both samples, a clear signal is present at 7.5 ppm for PMA-PEM. In combination with insights from ^{207}Pb NMR, this signal can be assigned to FA^{+} cations of the perovskite phase and further supports the successful embedding of the membrane with perovskite, as shown in supporting figure S3b.

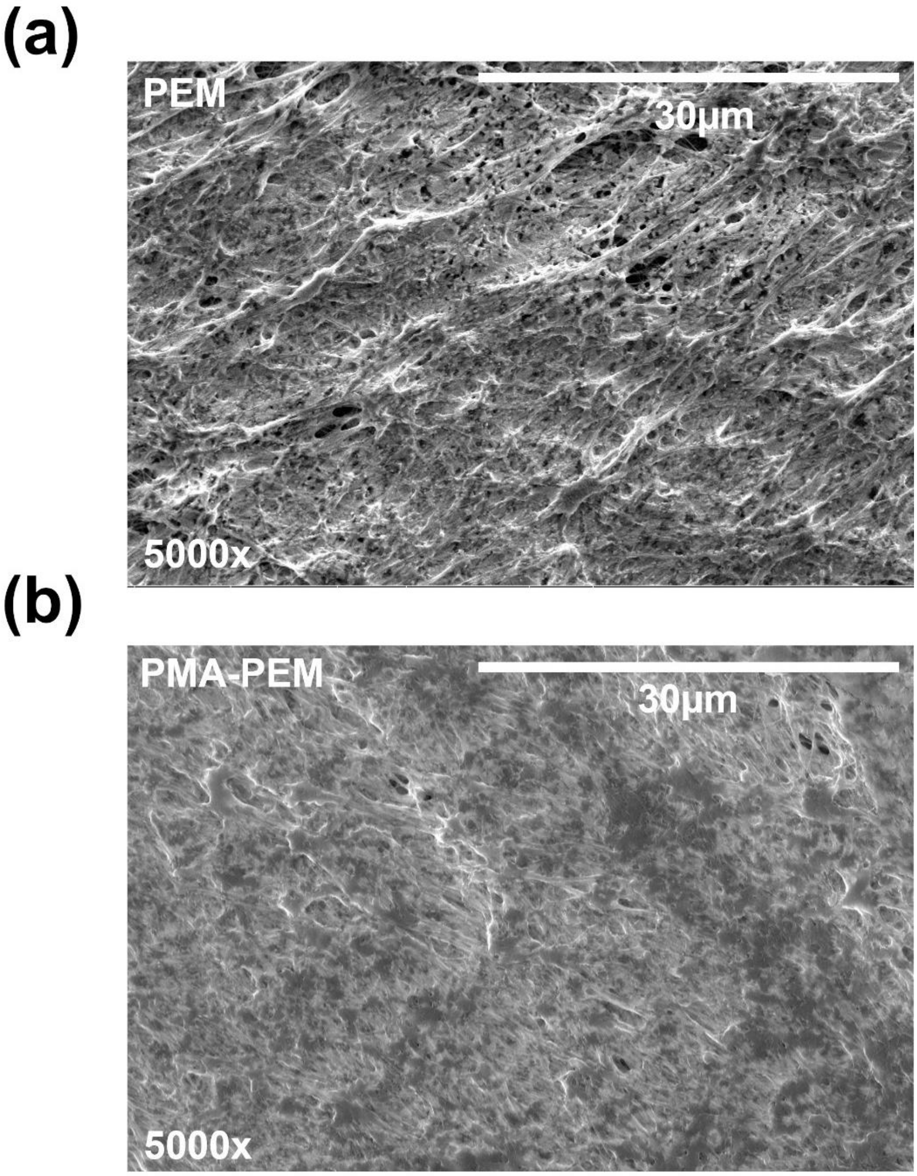

Figure S4. Top view SEM images of the perovskite embedded membranes without and with PMA at 5000x magnification. (a) PEM exhibits rough surface morphology with distinct perovskite aggregates and visible voids, showing incomplete coverage of the underlying membrane fibers. (b) In contrast, PMA-PEM shows a significantly smoother and more continuous surface. This indicates that the PMA additive promotes a more uniform film formation and better coverage of the membrane.

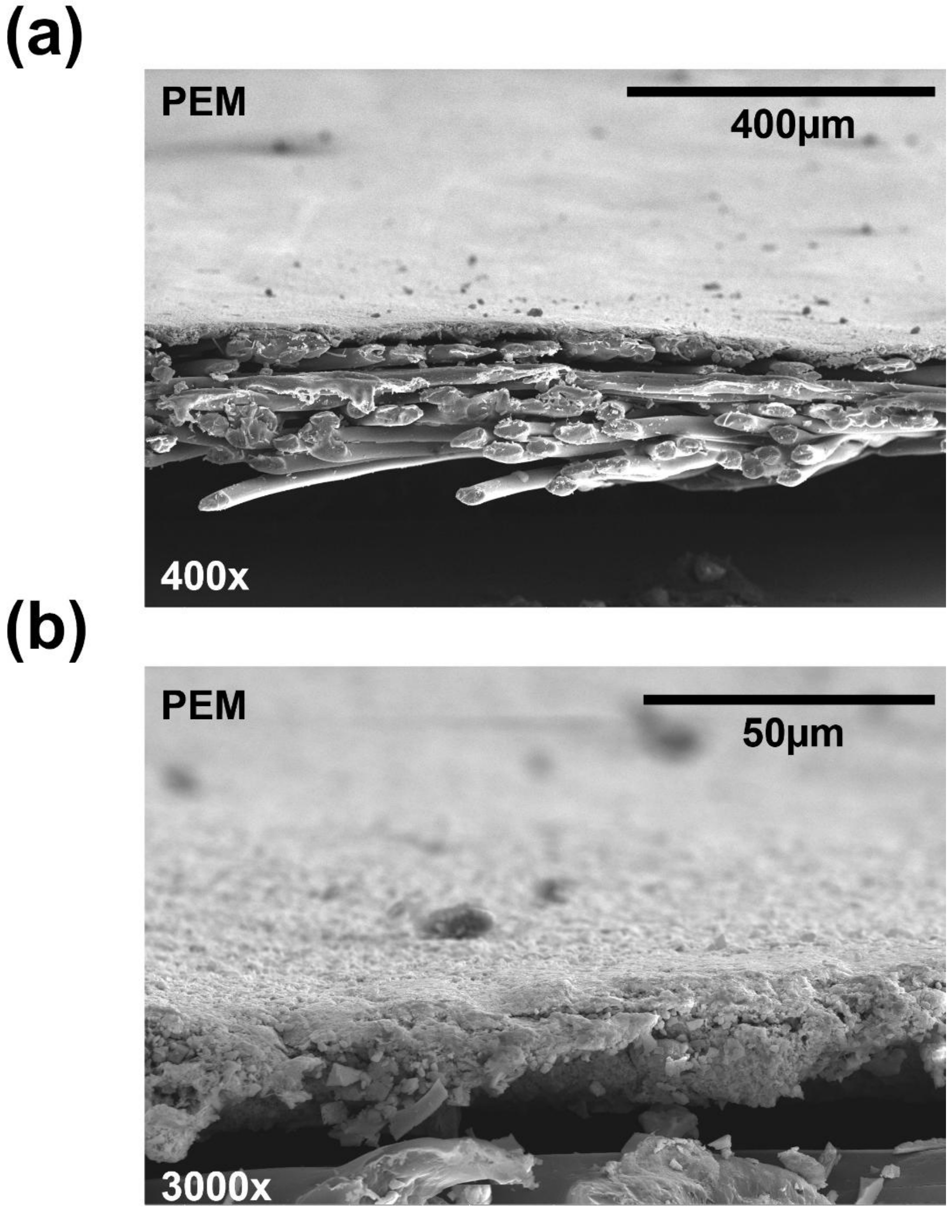

Figure S5. Cross-sectional SEM images of the PEM. (a) Low magnification (400x) image showing the overall cross-section of the thick perovskite layer supported by the porous membrane structure. (b) High magnification (3000x) image detailing the morphology of the embedded perovskite crystals on top of and within the fibrous support.

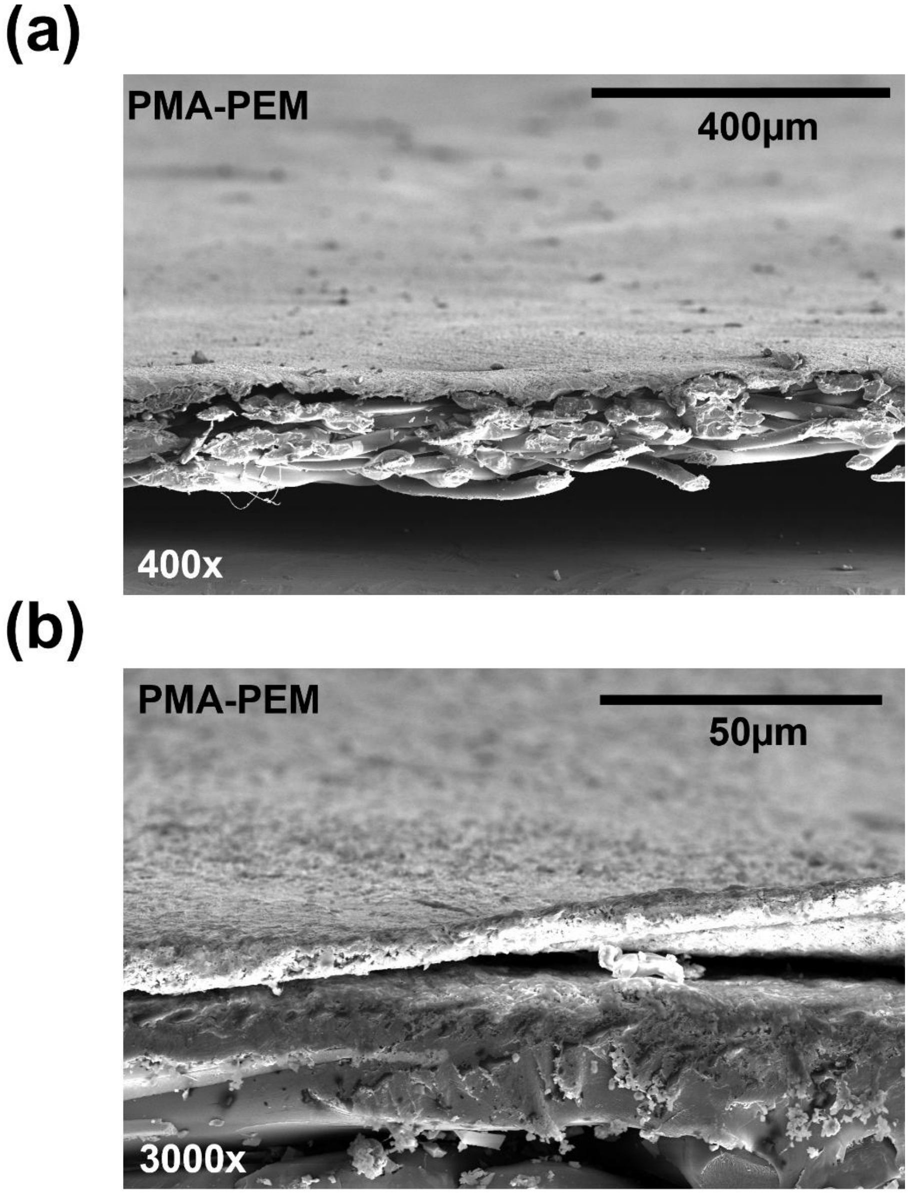

Figure S6. Cross-sectional SEM images of the PMA-PEM. (a) Low-magnification (400x) image showing the overall cross-section of the thick perovskite layer supported by the porous membrane structure. (b) High magnification (3000x) image detailing the morphology of the embedded perovskite crystals on top of and within the fibrous support.

Table S4. Gravimetric determination of perovskite mass loading and calculation of the equivalent active thickness and volume fraction for the perovskite embedded membrane.

**Parameter**	**Value**	**Unit**	**Method / Formula**
Sample Area (A)	~6.25	cm^2	Physical measurement (2.5 X 2.5)
Membrane Thickness	200	µm	Provided by manufacturer & Cross-sectional SEM
Average Infiltrated Mass of the perovskite ($m_{perovskite}$)	~72.52	mg	Gravimetric ($m_{after\ spin\ coating} - m_{before\ spin\ coating}$)
Average Mass Loading	~11.60	$mg.cm^{-2}$	$m_{perovskite}$ / A of the film
Theoretical Density ($\rho_{perovskite}$)	~4.09	$g.cm^{-3}$	Crystallographic calculation (based on unit cell volume)
Equivalent Active Thickness (t_{eq})	~28.4	µm	*Mass Loading* $X\ \rho_{perovskite}^{-1}$
Volume Fraction	~14.2	%	$(t_{eq})(t_{geo})^{-1}$ X 100

Theoretical Density of Perovskite ($\rho_{perovskite}$) = (Z * M) / (V * N_A) (4)

- Z: Number of formula units per unit cell (often 4 for cubic perovskites).
- M: Molar mass of the compound (grams/mole).
- V: Volume of the unit cell (cm^3).
- NA: Avogadro's number (6.022×10^{23} molecules/mole)

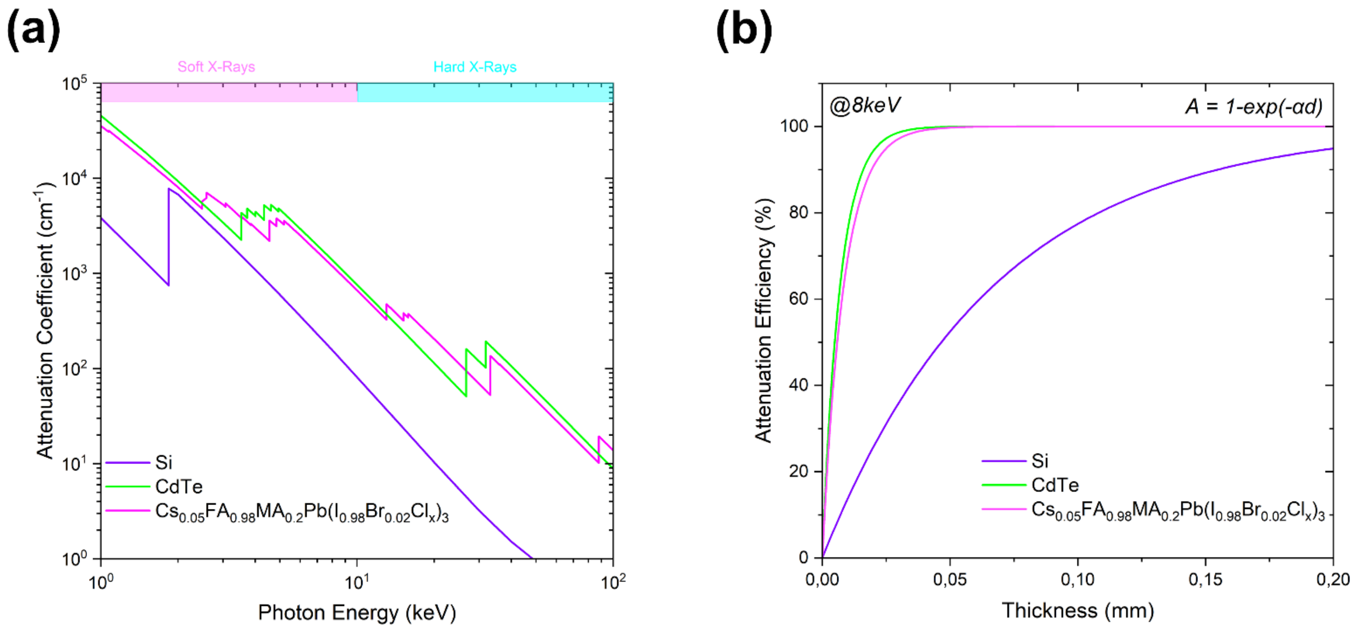

Figure S7. Theoretical X-ray attenuation properties of the triple cation perovskite, Si and CdTe. (a) The X-ray attenuation coefficients of Si (purple), CdTe (green), and triple cation perovskite, $Cs_{0.05}(MA_{0.02}FA_{0.98})_{0.95}Pb(I_{0.98}Br_{0.02}Cl_x)_3$ (magenta) as a function of photon energy, showing the superior absorption of heavy element materials particularly in the soft X-ray regime (1-20 keV). (b) Calculated X-ray attenuation efficiency at 8 keV as a function of material thickness, based on the Beer-Lambert law. The triple cation perovskite achieves >99% attenuation with only ~39 µm thickness, outperforming Si and comparable to CdTe, highlighting its potential for use in low energy X-ray detection.

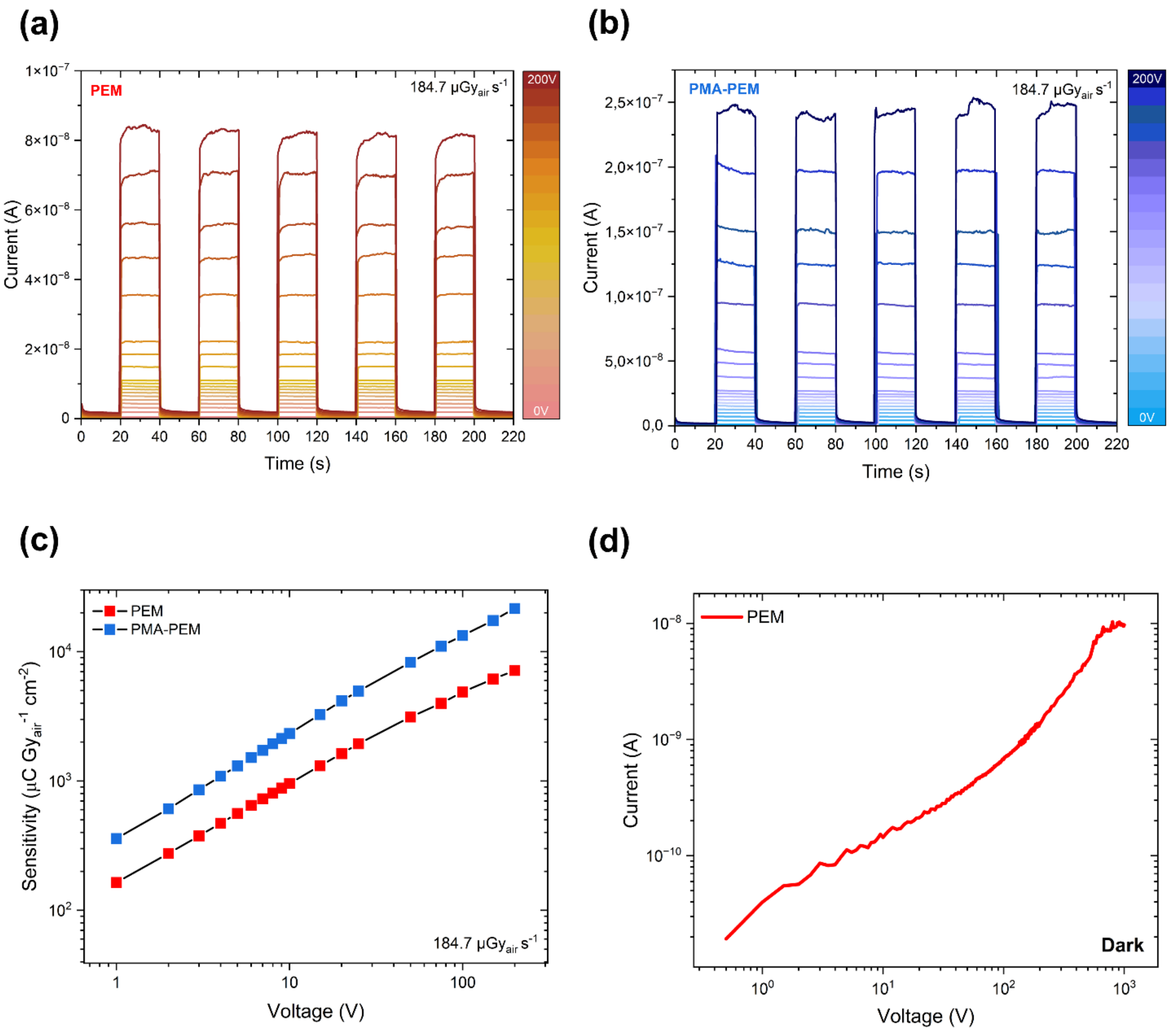

Figure S8. X-ray response and sensitivity of devices at different voltages under continuous Cu anode irradiation (primary Kα (8.05 keV) emission line). (a) Time dependent X-ray photocurrent response of PEM device at high dose rate of 184.7 µGy_{air} s^{-1}, measured under varying applied bias voltages (0-200 V). (b) Corresponding X-ray photocurrent response of the PMA-PEM device, demonstrating enhanced current generation compared to the PEM. (c) Calculated sensitivity as a function of applied voltage for PEM and PMA-PEM device under X-ray dose rate of 184.7 µGy_{air} s^{-1}. (d) The dark current versus voltage for PEM device. The absence of sudden current increase indicates a breaking voltage exceeding 1000 V bias, highlighting the high dielectric strength of the membrane-based architecture.

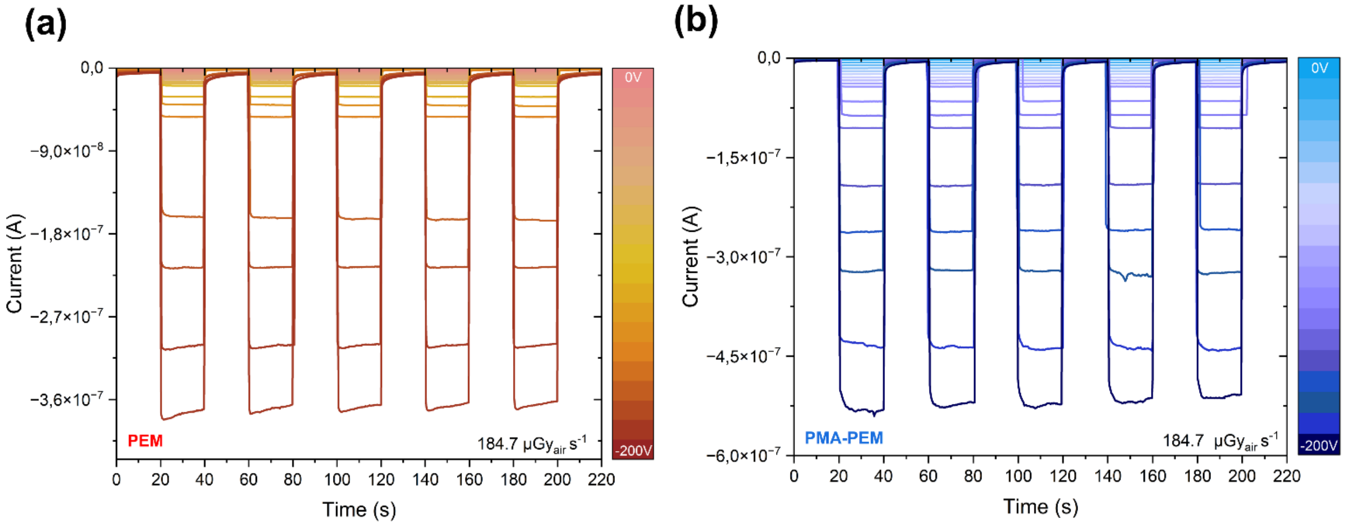

Figure S9. X-ray response of devices under continuous Cu anode irradiation (primary Kα (8.05 keV) emission line) at various negative bias voltages (a) Time-dependent X-ray photocurrent response of PEM device at high dose rate of 184.7 $\mu Gy_{air}\ s^{-1}$, measured under varying applied negative bias voltages (from 0 to -200 V). (b) Corresponding X-ray photocurrent response of the PMA-PEM device, showing enhanced current generation compared to PEM.

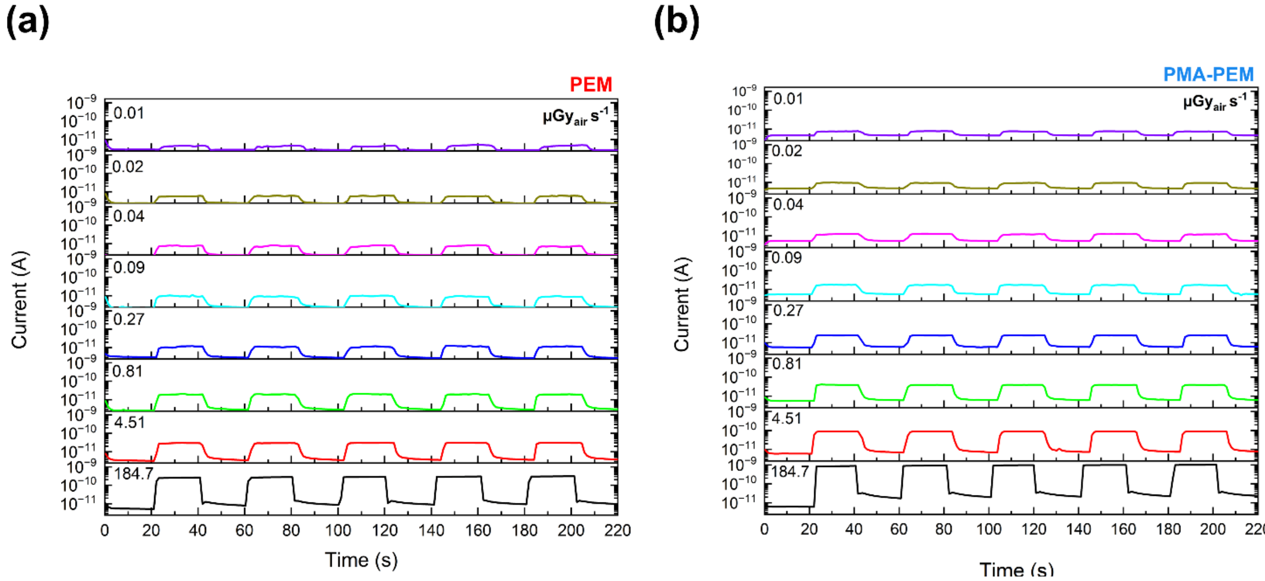

Figure S10. Dose rate-dependent current response of flexible perovskite X-ray detectors measured under continuous Cu anode irradiation (primary Kα (8.05 keV) emission line) at 0 V bias. (a) PEM device and (b) PMA-PEM device under varying X-ray dose rates. The stepwise increase in current corresponds to the on/off cycling of X-ray exposure. The PMA-PEM device demonstrates a higher photocurrent response compared to PEM, highlighting its enhanced sensitivity to X-ray irradiation.

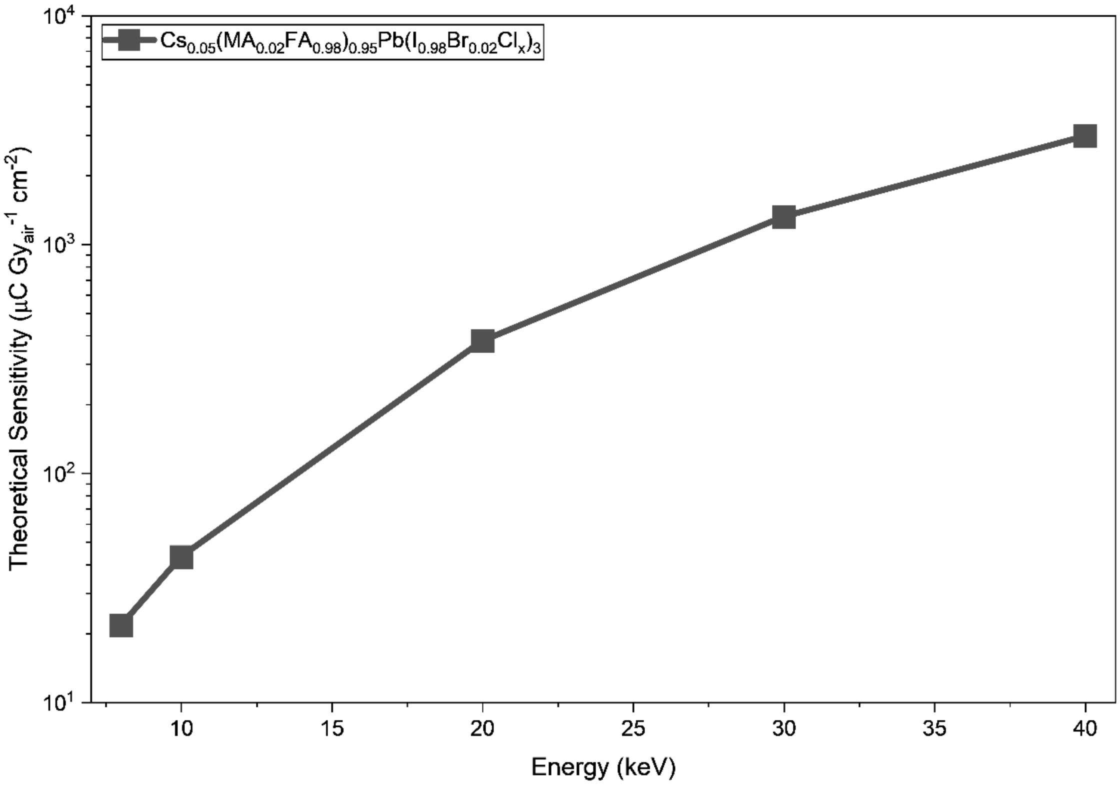

Figure S11. Calculated maximum theoretical sensitivity of the triple cation perovskite.

The X-ray source used in this study is a Cu target tube operated at an accelerating voltage of 40 kV. It is important to clarify that the typically stated energy of ~8.05 keV corresponds strictly to the unfiltered Cu Kα characteristic line. During our measurements, varying thicknesses of metal filters (0.1-0.3 mm (Cu) or 0.4+0.02 mm (Cu+Ni) plates) were used primarily to attenuate the X-ray intensity (dose rate). However, this attenuation introduces a spectral hardening effect: as the Cu filter thickness increases, the spectrum shifts toward higher energies, effectively modifying the average photon energy of the beam. Consequently, the resulting field is not strictly monochromatic; the devices are exposed to a spectrum of energies extending from the characteristic line up to the acceleration limit. To know the theoretical performance limits across this range, we performed calculations at both the characteristic and maximum energy levels.

At 8 keV (Unfiltered Characteristic Line): The calculated maximum theoretical sensitivity is ~21.63 μC $Gyair^{-1}$ cm^{-2} for our perovskite. Comparing this value to our experimental results reveals that the measured values are approximately ~ 4160 and ~ 10633 times higher for PEM and PMA-PEM, respectively. This magnitude of difference serves as strong evidence for a dominant photoconductive gain mechanism. At 40kV (corresponding to the maximum energy 40keV), the theoretical sensitivity for our perovskite is calculated to be ~2972 μC $Gyair^{-1}$ cm^{-2} (Figure S11).

The maximum theoretical sensitivity (S_0) is calculated buy using this equation (assuming no photoconductive gain):[30,64,65]

$$S_0 = \left(\frac{q \cdot 6.21 \cdot 10^{21}}{(\alpha_{air}/\rho_{air}) \cdot W}\right) \cdot \left(\frac{\alpha_{en}}{\alpha}\right) \tag{5}$$

In this expression, q represents the elementary electron charge, while α_{air} and ρ_{air} denote the energy absorption coefficient and density of air, respectively. Regarding the detector material, α and α_{en} correspond to the linear attenuation and energy absorption coefficients. The energy required for electron-hole pair creation, W, is derived from the bandgap energy (Eg) using the empirical relation W(eV) = 2.8Eg + 0.5.[66]

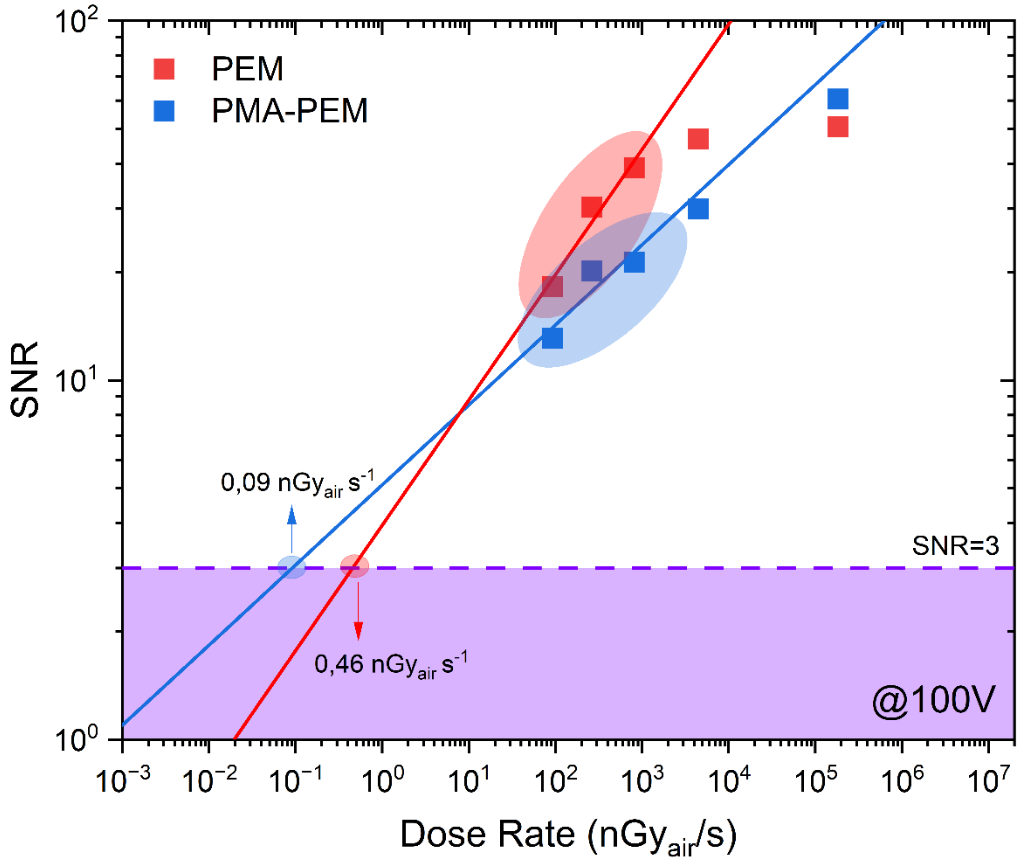

Figure S12. Determination of limit of detection (LoD) based on signal-to-noise ratio (SNR) SNR as a function of X-ray dose rate for PEM (red) and PMA-PEM (blue) perovskite detectors at 100 V bias. The calculated LoD values are 0.09 nGy$_{air}$ s^{-1} for the PMA-treated detector and 0.46 nGy$_{air}$ s^{-1} for PEM detector, demonstrating lower detection threshold for PMA-PEM device.

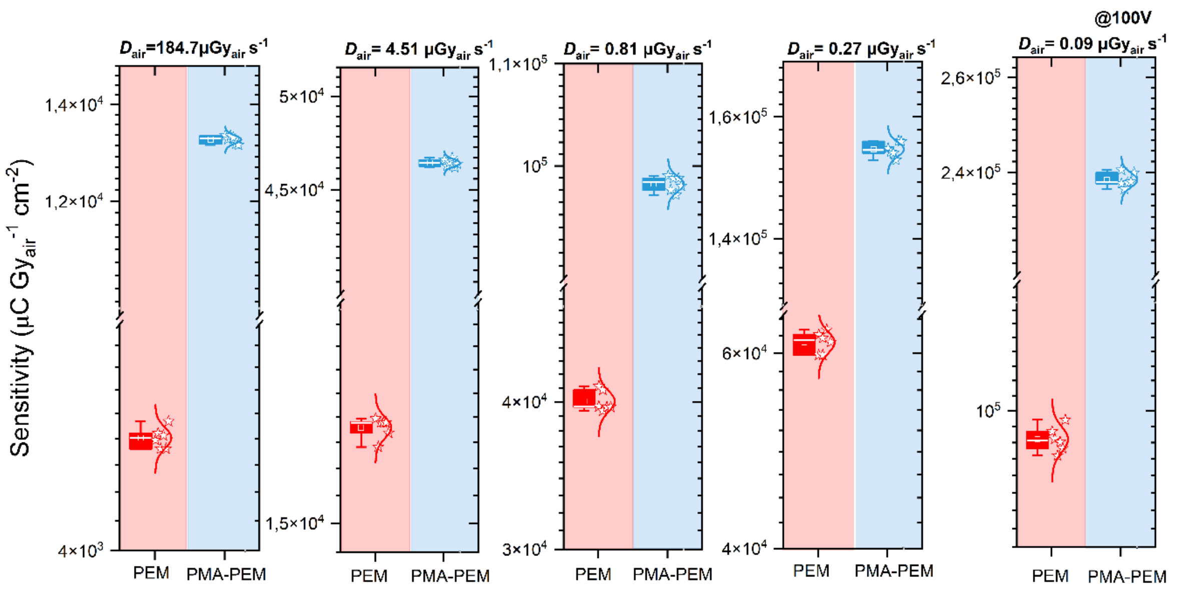

Figure S13. Device to device reproducibility of PEM and PMA-PEM devices at different X-ray dose rates. Box plots display the statistical distribution of sensitivities measured from six individual devices per group (PEM: red, PMA-PEM: blue) under a constant applied bias of 100 V. While sensitivity increases with decreasing dose rate, PMA-PEM detectors consistently exhibit higher mean sensitivity and narrower distributions, indicating both enhanced performance and improved fabrication reproducibility. The corresponding numerical values, including mean sensitivity, standard deviation (SD), and relative standard deviation (RSD), are summarized in Table S5.

Table S5. Statistical analysis of device to device reproducibility. Mean sensitivity, standard deviation (SD), and relative standard deviation (RSD) calculated from six PEM and six PMA-PEM devices at five different dose rates. All measurements were performed at an applied bias of 100 V.

Mean Sensitivity ($\mu Gy_{air} \cdot s^{-1} \cdot cm^{-2}$) PEM	Standard deviation in Sensitivity ($\mu Gy_{air} \cdot s^{-1} \cdot cm^{-2}$) PEM	Relative standard deviation in Sensitivity (%) PEM	Air dose rate $\mu Gy_{air} \cdot s^{-1}$
4894.75418	91.69984	1.87343	184.66325
16992.30725	233.68393	1.37523	4.51061
40045.12259	819.17956	2.04564	0.81258
61353.78708	1347.39836	2.19611	0.26527
96005.43126	1744.04692	1.81661	0.09391
Mean Sensitivity ($\mu Gy_{air} \cdot s^{-1} \cdot cm^{-2}$) PMA-PEM	Standard deviation (SD) in Sensitivity ($\mu Gy_{air} \cdot s^{-1} \cdot cm^{-2}$) PMA-PEM	Relative standard deviation (RSD) in Sensitivity (%) PMA-PEM	Air dose rate $\mu Gy_{air} \cdot s^{-1}$
13240.16427	85.3097	0.64433	184.66325
46386.16327	190.80159	0.41133	4.51061
98339.18189	704.31646	0.71621	0.81258
154396.27452	1239.02466	0.8025	0.26527
238471.81063	1457.11401	0.61102	0.09391

Table S6. Raw data for the mechanical flexibility tests shown in Figure 7. All sensitivity and retention values were measured at an applied bias of 100 V and a dose rate of 184.7 μGy_{air} s^{-1}.The table lists the data for the cyclic bending fatigue test, showing performance after a set number of bending cycles at a fixed radius of 0.6725 cm.

Bending Cycles (Bending Radius: 0.6725 cm)	Sensitivity ($\mu Gy_{air} \cdot s^{-1} \cdot cm^{-2}$) **PEM**	Sensitivity ($\mu Gy_{air} \cdot s^{-1} \cdot cm^{-2}$) **PMA-PEM**	Retention (%) **PEM**	Retention (%) **PMA-PEM**
Flat	4876.53715	13327.6152	100	100
100	4298.98135	13285.68101	88.15644	99.68536
500	2595.60472	10446.05885	53.22639	78.37905
1000	1463.58456	7463.49653	30.01278	56.00024

The formula relating to bending radius (R), chord length (c), and arc height (h) is:

$$R = (c^2 / 8h) + (h / 2) \quad (6)$$

Table S7. Raw data for the mechanical flexibility tests shown in Figure 7. All sensitivity and retention values were measured at an applied bias of 100 V and a dose rate of 184.7 $\mu Gy_{air}\ s^{-1}$. The table presents the performance data from static bending tests, where the device was subjected to a series of defined bending radii. For each bending condition, both the raw sensitivity and the corrected sensitivity values are provided. The corrected sensitivity values account for the change in the projected area of the pixel during bending. Since the pixel surface becomes tilted under bending, its projected area onto the detector plane decreases. This geometrical effect was considered by calculating the projected length based on the bending radius, allowing a more accurate comparison across different curvatures.

Bending Radius (cm)	Sensitivity ($\mu Gy_{air}\cdot s^{-1}\cdot cm^{-2}$) **PEM**	Sensitivity ($\mu Gy_{air}\cdot s^{-1}\cdot cm^{-2}$) **PMA-PEM**	Retention (%) **PEM**	Retention (%) **PMA-PEM**
Flat	4844.1693	13928.3149	100	100
4.3065	4214.3251	13371.18238	86.99789	96
2.776	3631.2492	12538.4688	74.96124	90.02143
2.2505	3207.0631	11483.9668	66.2046	82.45051
1.3525	2809.6221	10143.2707	58.00008	72.82482
0.741	2203.6416	8428.386	45.4906	60.5126
Bending Radius (cm)	Corrected Sensitivity ($\mu Gy_{air}\cdot s^{-1}\cdot cm^{-2}$) **PEM**	Corrected Sensitivity ($\mu Gy_{air}\cdot s^{-1}\cdot cm^{-2}$) **PMA-PEM**	Corrected Retention (%) **PEM**	Corrected Retention (%) **PMA-PEM**
Flat	4844.1693	13928.3149	100	100
4.3065	4256.88978	13506.23132	87.87657	96.9696
2.776	3705.363	12794.37895	76.4912	91.85877
2.2505	3306.22549	11839.05105	68.25165	84.99988
1.3525	3021.07426	10906.65325	62.36517	78.30562
0.741	2825.17871	10805.61227	58.32122	77.58018

During bending, the effective area of the pixel projected onto the detector plane decreases due to curvature. To account for this, the corrected sensitivity was calculated by geometrically correcting the pixel area using the projected length.

The projected length $\ell_{projected}$ was obtained via:

$$\ell_{projected} = \ell \cdot \cos\left(\left(d / (2 \cdot 2R \cdot \pi)\right) \cdot 360^\circ\right) \tag{7}$$

Where:
- ℓ is the original pixel length (in the bending direction)
- d is the distance between the pixels
- R is the bending radius
- The term inside the cosine corresponds to the angular tilt due to bending

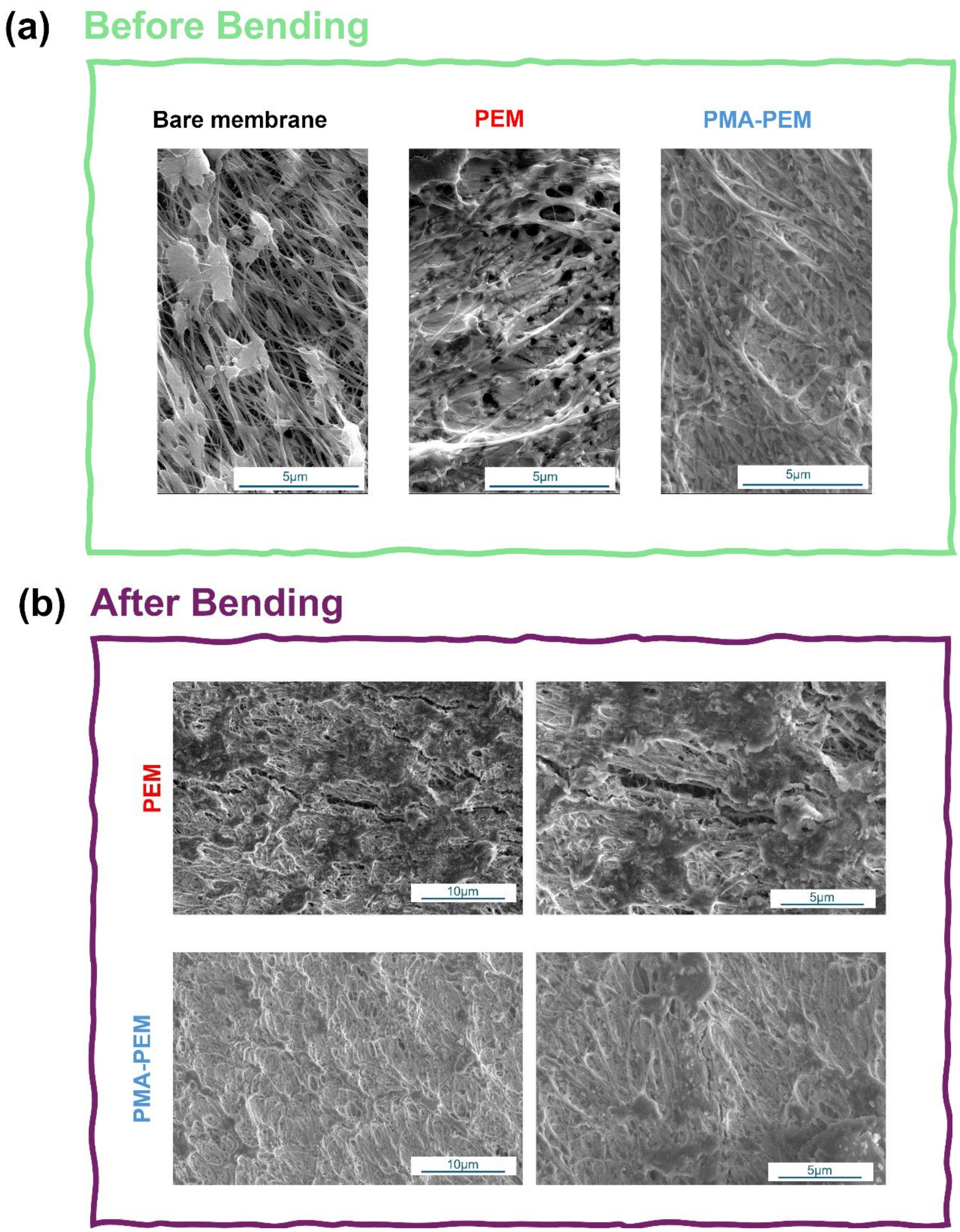

Figure S14. SEM images before and after bending cycles of 1000 times (a) SEM images of bare membrane, PEM and PMA-PEM. (b) SEM images of PEM and PMA-PEM after bending. PEM exhibits mechanical damage, characterized by a high density of wide, propagating cracks, typical of brittle fracture behavior. The PMA-PEM maintains a highly coherent and smooth morphology. Although minor hairline cracks are visible, their propagation and widening are effectively suppressed.

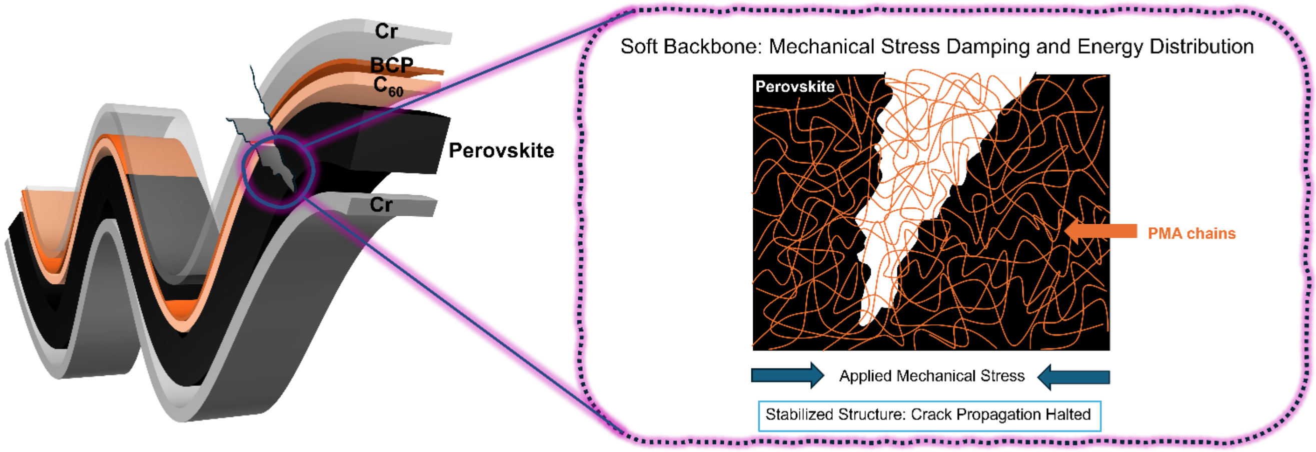

Figure S15. The schematic representation of how PMA prevents crack propagation. The cross-sectional stack of the flexible perovskite X-Ray Detector (left). Schematic representation of the soft backbone mechanism dissipating stress and bridge the film to maintain structural integrity (right).

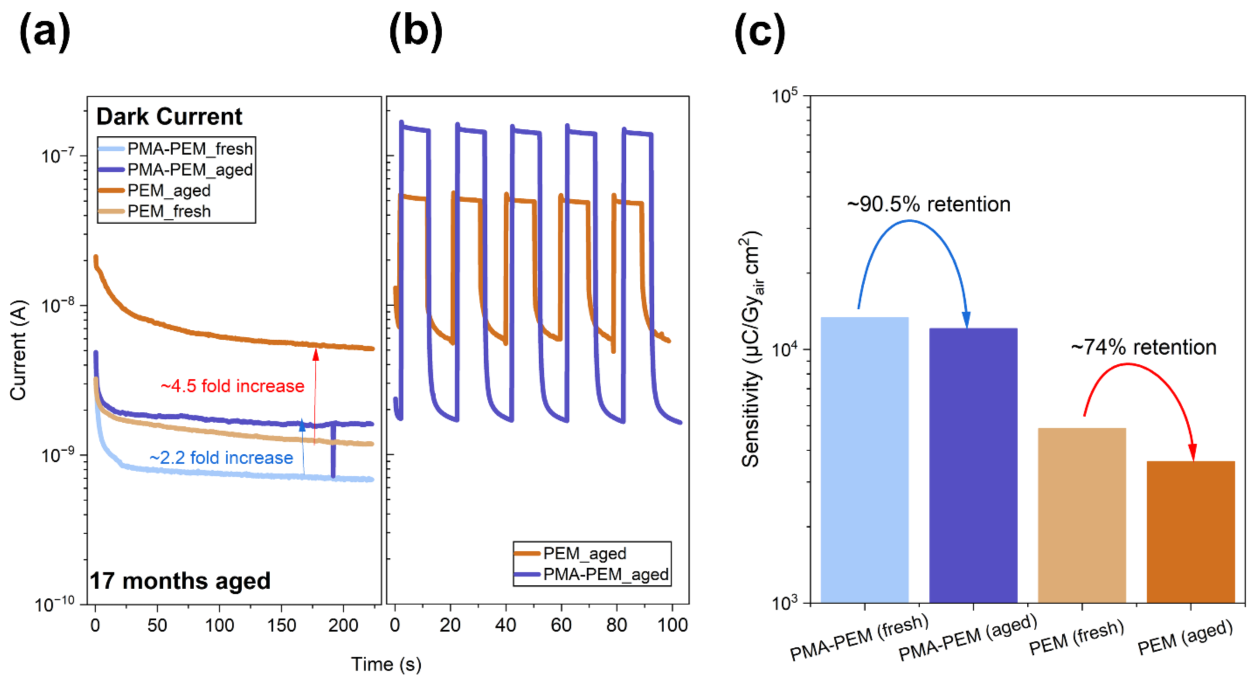

Figure S16. Long term ambient reliability of unencapsulated X-ray detectors. (a) Dark current transients measured at a bias of 100 V for PEM and PMA-PEM devices immediately after fabrication and after aging for 17 months in mixed ambient/glovebox conditions. The aged PEM device exhibits an increase in dark current by ~4.5 fold, whereas the PMA-PEM device shows increase by only ~2.2 fold. (b) X-ray response (on/off cycling) was recorded under 100 V bias and a dose rate of 184.7 $\mu Gy_{air}\ s^{-1}$. (c) Sensitivity retention comparison. PEM device retains ~74% of initial sensitivity, whereas PMA-PEM device retains ~90.5 %, demonstrating the effective protection provided by the PMA additive.

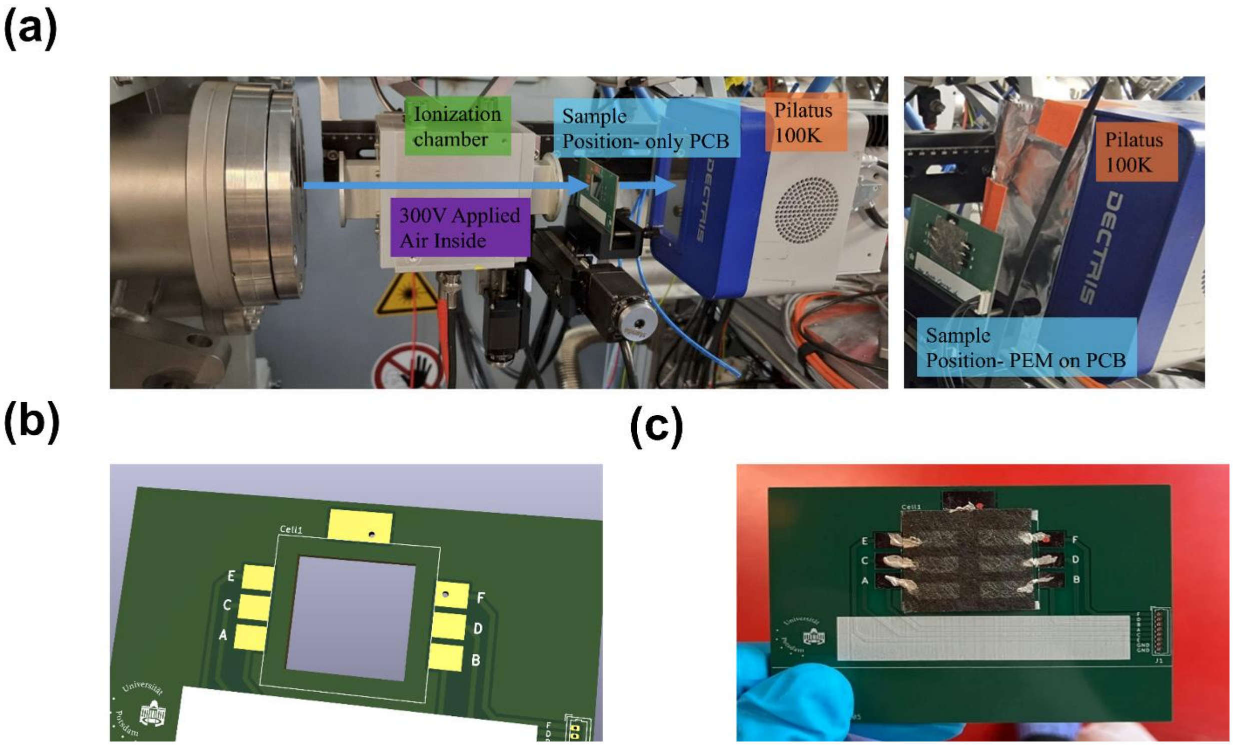

Figure S17. Experimental setup and PCB electronic circuit for beamtime measurements. a) Overview of the experimental setup at the beamline, including an ionization chamber, sample PCB at the sample position without shielding, and the Pilatus 100k detector and close-up of the sample mounted on the PCB in front of the Pilatus 100k detector. b) Schematic representation of the PCB circuit used for electronic connections. c) Actual device integration on the PCB, demonstrating the electrical contact configuration for in-situ X-ray response measurements.

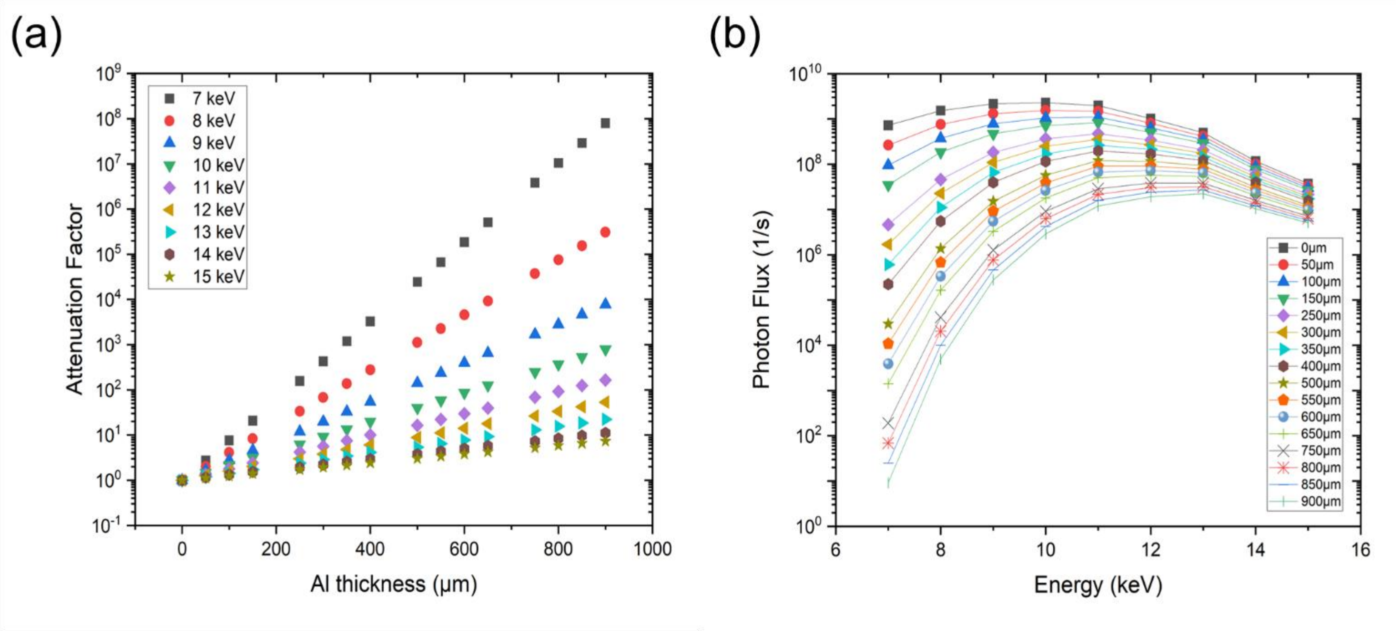

Figure S18. Attenuation Factor and Photon Flux as a Function of Al Thickness and Energy, respectively, at the KMC-3 XPP beamline at BESSY II. (a) Attenuation factor as a function of aluminum absorber thickness for different X-ray energies (7-15 keV). Increasing the absorber thickness leads to a significant reduction in photon flux, with lower-energy X-rays being attenuated more effectively. (b) Photon flux as a function of X-ray energy for different aluminum absorber thicknesses. As photon energy increases, the attenuation effect of the aluminum absorbers decreases, resulting in higher photon flux at higher X-ray energies. These measurements were used to calibrate the X-ray intensity for detector response evaluation.

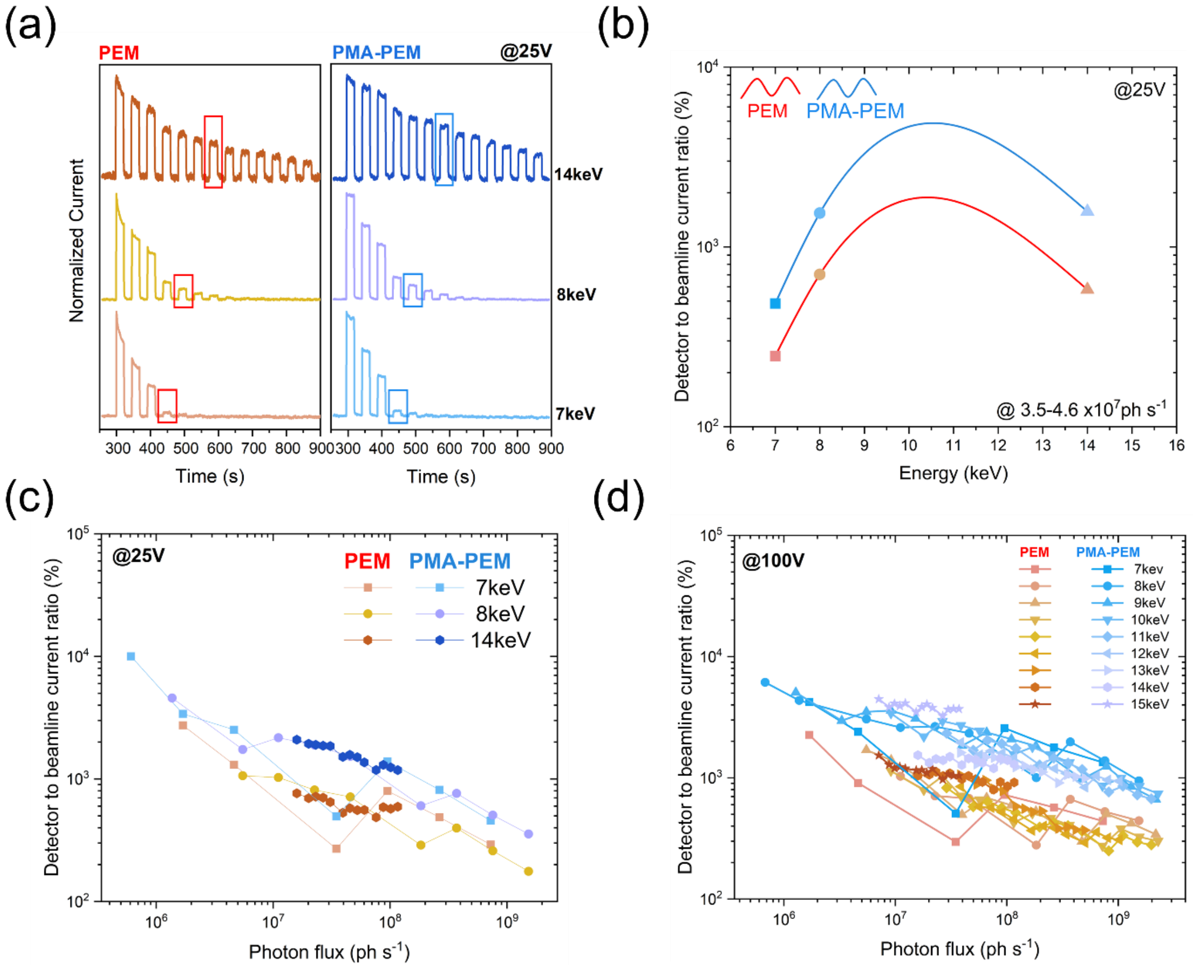

Figure S19. X-ray detection performance of flexible perovskite detectors under varying photon energy, flux and bias conditions at BESSY II. (a) The normalized time-dependent current response of PEM and PMA-PEM devices at 25 V bias under pulsed X-ray irradiation at different energies and photon fluxes. The boxes indicate the current response of the samples corresponding to the photon fluxes. The PMA-treated device exhibits a higher and more stable response. (b) Detector to beamline current ratio (%) as a function of photon flux at 25 V bias for PEM and PMA-PEM devices at different X-ray energies (7 keV, 8 keV, and 14 keV). (c) Detector to beamline current ratio (%) as a function of photon flux at bias voltage of 25 V, and (d) 100 V demonstrating the enhanced response in PMA-PEM devices compared to PEM.

References

(1) Li, Z.; Li, B.; Wu, X.; Sheppard, S. A.; Zhang, S.; Gao, D.; Long, N. J.; Zhu, Z. Organometallic-Functionalized Interfaces for Highly Efficient Inverted Perovskite Solar Cells. *Science (1979).* **2022**, *376* (6591), 416–420. https://doi.org/10.1126/science.abm8566.

(2) Holldack, K.; v. Hartrott, M.; Hoeft, F.; Neitzke, O.; Bauch, E.; Wahl, M. Bunch Fill Pattern at BESSY Monitored by Time-Correlated Single Photon Counting. *Advanced Photon Counting Techniques II* **2007**, *6771*, 281–288. https://doi.org/10.1117/12.734226.

(3) J. H. Hubbell, S. M. Seltzer, *NISTIR 5632: Tables of X-Ray Mass Attenuation Coefficients and Mass Energy-Absorption Coefficients 1 keV to 20 MeV for Elements Z = 1 to 92 and 48 Additional Substances of Dosimetric Interest N1'1S*, 1995. https://nvlpubs.nist.gov/nistpubs/legacy/ir/nistir5632.pdf

(4) Starkholm, A.; Al-Sabbagh, D.; Sarisozen, S.; von Reppert, A.; Rössle, M.; Ostermann, M.; Unger, E.; Emmerling, F.; Kloo, L.; Svensson, P. H.; Lang, F.; Maslyanchuk, O. Green Fabrication of Sulfonium-Containing Bismuth Materials for High-Sensitivity X-Ray Detection. *Advanced Materials* **2025**, *37* (24), 2418626. https://doi.org/10.1002/adma.202418626.

(5) Geng, X.; Chen, Y. A.; Li, Y. Y.; Ren, J.; Dun, G. H.; Qin, K.; Lin, Z.; Peng, J.; Tian, H.; Yang, Y.; Xie, D.; Ren, T. L. Lead-Free Halide Perovskites for Direct X-Ray Detectors. *Advanced Science* **2023**, *10* (23), 2300256. https://doi.org/10.1002/advs.202300256.

(6) Xiao, B.; Wang, F.; Xu, M.; Liu, X.; Sun, Q.; Zhang, B. Bin; Jie, W.; Sellin, P.; Xu, Y. Melt-Grown Large-Sized Cs2TeI6crystals for X-Ray Detection. *CrystEngComm* **2020**, *22* (31), 5130–5136. https://doi.org/10.1039/d0ce00868k.

(7) Chu, D.; Liu, N.; Xie, S.; Li, Y.; Chen, J.; Wei, M.; Feng, Z.; Zhao, L.; Jia, B.; Jiang, Y.; Pi, J.; Shi, R.; Yue, S.; Liu, Y.; Liu, S. Stable and Ultrasensitive X-Ray Detectors Based on Oriented Single-Crystal Perovskite Rods. *Advanced Materials* **2025**, *37* (27), 2500101. https://doi.org/10.1002/adma.202500101.

(8) Thompson, M.; Ellison, S. L. R.; Wood, R. Resulting from the Symposium on Harmonization of Quality Assurance Systems for Analytical Laboratories. *Pure Appl. Chem* **2002**, *74* (5), 835–855. https://doi.org/10.1351/pac200274050835.

(9) Dong, H.; Liu, X.; Wang, H.; Chen, Z.; Li, F.; Wang, P.; Fu, J.; Shao, Y. An X-Ray Detector with an Ultra-Low Detection Limit Based on Bulk Two-Dimensional Perovskite PEA2PbBr4 Single Crystals Grown in HBr Solution. *J. Mater. Chem. A Mater.* **2024**. *12* (43), 29785-29794. https://doi.org/10.1039/d4ta04726e.

(10) He, Y.; Hadar, I.; De Siena, M. C.; Klepov, V. V.; Pan, L.; Chung, D. Y.; Kanatzidis, M. G. Sensitivity and Detection Limit of Spectroscopic-Grade Perovskite CsPbBr3 Crystal for Hard X-Ray Detection. *Adv. Funct. Mater.* **2022**, *32* (24), 2112925. https://doi.org/10.1002/adfm.202112925.

(11) Song, Y.; Li, L.; Bi, W.; Hao, M.; Kang, Y.; Wang, A.; Wang, Z.; Li, H.; Li, X.; Fang, Y.; Yang, D.; Dong, Q. Atomistic Surface Passivation of CH 3 NH 3 PbI 3 Perovskite Single Crystals for Highly Sensitive Coplanar-Structure X-Ray Detectors. *Research* **2020**, *2020,* 5958243.

https://doi.org/10.34133/2020/5958243.

(12) Wei, H.; Desantis, D.; Wei, W.; Deng, Y.; Guo, D.; Savenije, T. J.; Cao, L.; Huang, J. Dopant Compensation in Alloyed CH3NH3PbBr3-x Clx Perovskite Single Crystals for Gamma-Ray Spectroscopy. *Nat. Mater.* **2017**, *16* (8), 826–833. https://doi.org/10.1038/nmat4927.

(13) Liu, Y.; Zhang, Y.; Zhu, X.; Feng, J.; Spanopoulos, I.; Ke, W.; He, Y.; Ren, X.; Yang, Z.; Xiao, F.; Zhao, K.; Kanatzidis, M.; Liu, S. Triple-Cation and Mixed-Halide Perovskite Single Crystal for High-Performance X-Ray Imaging. *Advanced Materials* **2021**, *33* (8), 2006010. https://doi.org/10.1002/adma.202006010.

(14) Xiao, Y.; Jia, S.; Bu, N.; Li, N.; Liu, Y.; Liu, M.; Yang, Z.; Liu, S. Grain and Stoichiometry Engineering for Ultra-Sensitive Perovskite X-Ray Detectors. *J. Mater. Chem. A Mater.* **2021**, *9* (45), 25603–25610. https://doi.org/10.1039/d1ta07585c.

(15) Ba, Y.; Han, Y.; Zhu, W.; Wang, T.; Chi, J.; Xi, H.; Zhao, T.; Chen, D.; Zhang, J.; Zhang, C.; Hao, Y. Water-Assisted Mass Preparation of CsPbBr3-CsPb2Br5-CsPbIxBr3-x Composite Wafers for High-Performance X-Ray Detection. *Chemical Engineering Journal* **2024**, *479*, 147726. https://doi.org/10.1016/j.cej.2023.147726.

(16) Chen, M.; Zheng, X.; Li, Y.; Dong, X.; Pi, J.; Chu, D.; Liu, N.; Jia, B.; Liang, Y.; Zhang, X.; Zhao, Z.; Hao, J.; Zhao, L.; Feng, Z.; Wei, M.; Shi, R.; Liu, S.; Liu, Y. Distorted Lead-Free Germanium-Based Perovskite Single-Crystals for Stable and Efficient X-Ray Detection and Imaging. *Adv. Opt. Mater.* **2024**, *12* (24), 2401028. https://doi.org/10.1002/adom.202401028.

(17) Shi, T.; Liu, W.; Zhu, J.; Fan, X.; Zhang, Z.; He, X.; He, R.; Wang, J.; Chen, K.; Ge, Y.; Sun, X.; Liu, Y.; Chu, P. K.; Yu, X. F. CsPbBr3-DMSO Merged Perovskite Micro-Bricks for Efficient X-Ray Detection. *Nano Res.* **2023**, *16* (7), 9983–9989. https://doi.org/10.1007/s12274-023-5487-3.

(18) Zheng, X.; Zhao, W.; Wang, P.; Tan, H.; Saidaminov, M. I.; Tie, S.; Chen, L.; Peng, Y.; Long, J.; Zhang, W. H. Ultrasensitive and Stable X-Ray Detection Using Zero-Dimensional Lead-Free Perovskites. *Journal of Energy Chemistry* **2020**, *49*, 299–306. https://doi.org/10.1016/j.jechem.2020.02.049.

(19) Chu, D.; Jia, B.; Liu, N.; Zhang, Y.; Li, X.; Feng, J.; Pi, J.; Yang, Z.; Zhao, G.; Liu, Y.; Liu, S. Lattice Engineering for Stabilized Black FAPbI 3 Perovskite Single Crystals for High-Resolution x-Ray Imaging at the Lowest Dose. *Sci. Adv.* **2023**, *9* (35), eadh2255.

(20) Huang, Y.; Qiao, L.; Jiang, Y.; He, T.; Long, R.; Yang, F.; Wang, L.; Lei, X.; Yuan, M.; Chen, J. A-site Cation Engineering for Highly Efficient MAPbI 3 Single-Crystal X-ray Detector . *Angewandte Chemie* **2019**, *131* (49), 17998–18006. https://doi.org/10.1002/ange.201911281.

(21) Jiang, W.; Li, H.; Liu, D.; Ren, J.; Zhao, Y.; Wu, J.; Chen, J.; Zhou, L.; Wang, F.; Zhao, Y. Synergetic Electrostatic and Steric Effects in α-FAPbI3 Single Crystals For X-Ray Detection and Imaging. *Small* **2024**, *20* (38), 2402277. https://doi.org/10.1002/smll.202402277.

(22) Tan, W.; Xiao, Y.; Zhou, C.; Jin, X.; Zhu, S.; Han, M.; Tang, Z.; Zhang, Y.; Su, Z.; Chen, T.; Chen, Q.; Liang, Q.; Chen, W.; Jiang, Y. Transparent Perovskite Wafers via Nanocrystals Ordered Coalescence Toward Sensitive and Stable X-Ray Detection and Imaging. *Adv. Funct. Mater.* **2024**, *34* (42), 2406839. https://doi.org/10.1002/adfm.202406839.

(23) Jia, S.; Xiao, Y.; Bu, N.; Li, N.; Li, D.; Yang, Z.; Liu, S. Sintered Polycrystalline BiVO4 Pellet for Stable X-Ray Detector with Low Detection Limit. *Adv. Funct. Mater.* **2023**, *33* (16), 2213563. https://doi.org/10.1002/adfm.202213563.

(24) Tie, S.; Zhao, W.; Xin, D.; Zhang, M.; Long, J.; Chen, Q.; Zheng, X.; Zhu, J.; Zhang, W. H. Robust Fabrication of Hybrid Lead-Free Perovskite Pellets for Stable X-Ray Detectors with Low Detection Limit. *Advanced Materials* **2020**, *32* (31), 2001981. https://doi.org/10.1002/adma.202001981.

(25) Wu, W.; Zhang, J.; Liu, C.; Zhang, J.; Lai, H.; Hu, Z.; Zhou, H. Spontaneous Cooling Enables High-Quality Perovskite Wafers for High-Sensitivity X-Ray Detectors with a Low-Detection Limit. *Advanced Science* **2024**, *11* (46), 2410303. https://doi.org/10.1002/advs.202410303.

(26) Wang, Y.; Zhang, S.; Wang, Y.; Yan, J.; Yao, X.; Xu, M.; Lei, X. W.; Lin, G.; Yue, C. Y. 0D Triiodide Hybrid Halide Perovskite for X-Ray Detection. *Chemical Communications* **2023**, *59* (60), 9239–9242. https://doi.org/10.1039/d3cc01183f.

(27) Wang, L.; Yan, Y.; Bu, M.; Wang, J.; Li, L.; Li, Y.; Liu, H.; Zhang, H.; Pi, X.; Yang, D.; Fang, Y. Silicone-Assisted Autonomous Growth of Strainless Perovskite Single Crystals for Integrated Low-Dose X-Ray Imaging Arrays. *Adv. Funct. Mater.* **2024**, *35* (7), 2415378. https://doi.org/10.1002/adfm.202415378.

(28) Zhang, J.; Li, A.; Li, B.; Yang, M.; Hao, X.; Wu, L.; Zhao, D.; Xia, G.; Ren, Z.; Tian, W.; Yang, D.; Zhang, J. Top-Seed Solution-Based Growth of Perovskite Cs3Bi2I9Single Crystal for High Performance X-Ray Detection. *ACS Photonics* **2022**, *9* (2), 641–651. https://doi.org/10.1021/acsphotonics.1c01647.

(29) Wei, H.; Huang, J. Halide Lead Perovskites for Ionizing Radiation Detection. *Nat. Commun.* **2019**, *10* (1), 1066. https://doi.org/10.1038/s41467-019-08981-w.

(30) Kasap, S. O. X-Ray Sensitivity of Photoconductors: Application to Stabilized a-Se. *J. Phys. D Appl. Phys.* **2000**, *33* (21), 2853. https://doi.org/10.1088/0022-3727/33/21/326.

(31) Zentai, G.; Partain, L.; Pavlyuchkova, R.; Proano, C.; Virshup, G.; Melekhov, L.; Zuck, A.; Breen, B. N.; Dagan, O.; Vilensky, A.; Schieber, M.; Gilboa, H.; Bennet, P.; Shah, K.; Dmitriev, Y.; Thomas, J.; Yaffe, M.; Hunter, D. Mercuric Iodide and Lead Iodide X-Ray Detectors for Radiographic and Fluoroscopic Medical Imaging. *Physics of Medical Imaging* **2003**, *5030*, 77–91.

(32) Song, Y.; Li, L.; Hao, M.; Bi, W.; Wang, A.; Kang, Y.; Li, H.; Li, X.; Fang, Y.; Yang, D.; Dong, Q. Elimination of Interfacial-Electrochemical-Reaction-Induced Polarization in Perovskite Single Crystals for Ultrasensitive and Stable X-Ray Detector Arrays. *Advanced Materials* **2021**, *33* (52), 2103078. https://doi.org/10.1002/adma.202103078.

(33) Jiang, J.; Xiong, M.; Fan, K.; Bao, C.; Xin, D.; Pan, Z.; Fei, L.; Huang, H.; Zhou, L.; Yao, K.; Zheng, X.; Shen, L.; Gao, F. Synergistic Strain Engineering of Perovskite Single Crystals for Highly Stable and Sensitive X-Ray Detectors with Low-Bias Imaging and Monitoring. *Nat. Photonics* **2022**, *16* (8), 575–581. https://doi.org/10.1038/s41566-022-01024-9.

(34) Guo, J.; Xu, Y.; Yang, W.; Xiao, B.; Sun, Q.; Zhang, X.; Zhang, B.; Zhu, M.; Jie, W. High-Stability Flexible X-Ray Detectors Based on Lead-Free Halide Perovskite Cs2TeI6Films. *ACS Appl. Mater. Interfaces* **2021**, *13* (20), 23928–23935. https://doi.org/10.1021/acsami.1c04252.

(35) Pan, W.; Yang, B.; Niu, G.; Xue, K. H.; Du, X.; Yin, L.; Zhang, M.; Wu, H.; Miao, X. S.; Tang, J. Hot-Pressed CsPbBr3 Quasi-Monocrystalline Film for Sensitive Direct X-Ray Detection. *Advanced Materials* **2019**, *31* (44), 1904405. https://doi.org/10.1002/adma.201904405.

(36) Qian, W.; Xu, X.; Wang, J.; Xu, Y.; Chen, J.; Ge, Y.; Chen, J.; Xiao, S.; Yang, S. An Aerosol-Liquid-Solid Process for the General Synthesis of Halide Perovskite Thick Films for Direct-Conversion X-Ray Detectors. *Matter* **2021**, *4* (3), 942–954. https://doi.org/10.1016/j.matt.2021.01.020.

(37) Lu, Y.; He, D.; Yuan, X.; Yan, Q.; Shu, X.; Hu, Z.; Zhang, Z.; Liu, Z.; Jiang, Z.; Xu, R.; Wang, W.; Ma, Z.; Chen, T.; Xu, H.; Xu, F.; Hong, F.; Song, H. CsPbIBr2 Thick Film With (100)-Preferred Orientation Enables Highly Sensitive X-Ray Detector by a Spray-Coating Method via Dual-Additive-Assisted Solution Strategy under Atmospheric Environment. *Adv. Funct. Mater.* **2024**, *35* (8), 2413507. https://doi.org/10.1002/adfm.202413507.

(38) Liu, X.; Li, H.; Cui, Q.; Wang, S.; Ma, C.; Li, N.; Bu, N.; Yang, T.; Song, X.; Liu, Y.; Yang, Z.; Zhao, K.; Liu, S. Molecular Doping of Flexible Lead-Free Perovskite-Polymer Thick Membranes for High-Performance X-Ray Detection. *Angewandte Chemie - International Edition* **2022**, *61* (41), e202209320. https://doi.org/10.1002/anie.202209320.

(39) Dong, K.; Zhou, H.; Shao, W.; Gao, Z.; Yao, F.; Xiao, M.; Li, J.; Liu, Y.; Wang, S.; Zhou, S.; Cui, H.; Qin, M.; Lu, X.; Tao, C.; Ke, W.; Fang, G. Perovskite-like Silver Halide Single-Crystal Microbelt Enables Ultrasensitive Flexible X-Ray Detectors. *ACS Nano* **2023**, *17* (2), 1495–1504. https://doi.org/10.1021/acsnano.2c10318.

(40) Qian, W.; Qiu, W.; Yu, S.; Huang, D.; Lei, R.; Huang, X.; Xiao, S.; Wang, X.; Yang, S. Solvent Engineering of MAPbI3 Perovskite Thick Film for a Direct X-Ray Detector. *Nanoscale* **2023**, *15* (14), 6664–6672. https://doi.org/10.1039/d2nr07016b.

(41) Li, H.; Li, T.; Ma, C.; Liu, X.; Lang, L.; Yang, T.; Song, X.; Cui, Q.; Yang, Z.; Liu, S.; Zhao, K. "One-Click Restart" Recycling of Metal-Free Perovskite X-Ray Detectors. *Advanced Materials* **2024**, *36* (26), 2400783. https://doi.org/10.1002/adma.202400783.

(42) Glushkova, A.; Andričević, P.; Smajda, R.; Náfrádi, B.; Kollár, M.; Djokić, V.; Arakcheeva, A.; Forró, L.; Pugin, R.; Horváth, E. Ultrasensitive 3D Aerosol-Jet-Printed Perovskite X-Ray Photodetector. *ACS Nano* **2021**, *15* (3), 4077–4084. https://doi.org/10.1021/acsnano.0c07993.

(43) Peng, J.; Xu, Y.; Yao, F.; Huang, H.; Li, R.; Lin, Q. Ion-Exchange-Induced Slow Crystallization of 2D-3D Perovskite Thick Junctions for X-Ray Detection and Imaging. *Matter* **2022**, *5* (7), 2251–2264. https://doi.org/10.1016/j.matt.2022.04.030.

(44) Jin, P.; Tang, Y.; Li, D.; Wang, Y.; Ran, P.; Zhou, C.; Yuan, Y.; Zhu, W.; Liu, T.; Liang, K.; Kuang, C.; Liu, X.; Zhu, B.; Yang, Y. (Michael). Realizing Nearly-Zero Dark Current and Ultrahigh Signal-to-Noise Ratio Perovskite X-Ray Detector and Image Array by Dark-Current-Shunting Strategy. *Nat. Commun.* **2023**, *14* (1), 626. https://doi.org/10.1038/s41467-023-36313-6.

(45) Li, W. G.; Wang, X. D.; Huang, Y. H.; Kuang, D. Bin. Ultrasound-Assisted Crystallization Enables Large-Area Perovskite Quasi-Monocrystalline Film for High-Sensitive X-Ray Detection and Imaging. *Advanced Materials* **2023**, *35* (31), 2210878. https://doi.org/10.1002/adma.202210878.

(46) Liu, X.; Cui, Q.; Li, H.; Wang, S.; Zhang, Q.; Huang, W.; Liu, C.; Cai, W.; Li, T.; Yang, Z.; Ma, C.; Ren, L.; Liu, S. F.; Zhao, K. Biocompatible Metal-Free Perovskite Membranes for Wearable X-Ray Detectors. *ACS Appl. Mater. Interfaces* **2024**, *16* (13), 16300–16308. https://doi.org/10.1021/acsami.4c01069.

(47) Dong, S.; Fan, Z.; Wei, W.; Tie, S.; Yuan, R.; Zhou, B.; Yang, N.; Zheng, X.; Shen, L. Bottom-up Construction of Low-Dimensional Perovskite Thick Films for High-Performance X-Ray Detection and Imaging. *Light Sci. Appl.* **2024**, *13* (1), 174. https://doi.org/10.1038/s41377-024-01521-2.

(48) Chen, S.; Liu, W.; Xu, M.; Shi, P.; Zhu, M. Electrospray Prepared Flexible CsPbBr3 Perovskite Film for Efficient X-Ray Detection. *J. Mater. Chem. C Mater.* **2023**, *11* (25), 8431–8437. https://doi.org/10.1039/d3tc01347b.

(49) Liu, Y.; Gao, C.; Li, D.; Zhang, X.; Zhu, J.; Wu, M.; Liu, W.; Shi, T.; He, X.; Wang, J.; Huang, H.; Sheng, Z.; Liang, D.; Yu, X. F.; Zheng, H.; Sun, X.; Ge, Y. Dynamic X-Ray Imaging with Screen-Printed Perovskite CMOS Array. *Nat. Commun.* **2024**, *15* (1), 1588. https://doi.org/10.1038/s41467-024-45871-2.

(50) Lédée, F.; Ciavatti, A.; Verdi, M.; Basiricò, L.; Fraboni, B. Ultra-Stable and Robust Response to X-Rays in 2D Layered Perovskite Micro-Crystalline Films Directly Deposited on Flexible Substrate. *Adv. Opt. Mater.* **2022**, *10* (1), 2101145. https://doi.org/10.1002/adom.202101145.

(51) Huang, H.; Zheng, Y.; Liu, C.; Zhang, Z.; Gao, M.; Wang, J.; Liu, Y.; Chu, P. K.; Yu, X. F. Interfacial Engineering Enables Perovskite Heteroepitaxial Growth on Black Phosphorus for Flexible X-Ray Detectors. *Small* **2023**, *19* (46), 2303229. https://doi.org/10.1002/smll.202303229.

(52) Zhao, J.; Xu, Y.; Peng, G.; Wu, Y.; Wang, Q.; Jin, Z. Lead-Free Metal Halides for a Stable, Flexible, and High-Performance X-Ray Detector. *J. Mater. Chem. C Mater.* **2025**, *13* (9), 4626–4633. https://doi.org/10.1039/d4tc04083j.

(53) Mescher, H.; Schackmar, F.; Eggers, H.; Abzieher, T.; Zuber, M.; Hamann, E.; Baumbach, T.; Richards, B. S.; Hernandez-Sosa, G.; Paetzold, U. W.; Lemmer, U. Flexible Inkjet-Printed Triple Cation Perovskite X-Ray Detectors. *ACS Appl. Mater. Interfaces* **2020**, *12* (13), 15774–15784. https://doi.org/10.1021/acsami.9b14649.

(54) Liu, J.; Shabbir, B.; Wang, C.; Wan, T.; Ou, Q.; Yu, P.; Tadich, A.; Jiao, X.; Chu, D.; Qi, D.; Li, D.; Kan, R.; Huang, Y.; Dong, Y.; Jasieniak, J.; Zhang, Y.; Bao, Q. Flexible, Printable Soft-X-Ray Detectors Based on All-Inorganic Perovskite Quantum Dots. *Advanced Materials* **2019**, *31* (30), 1901644. https://doi.org/10.1002/adma.201901644.

(55) Li, H.; Wang, C. feng; Luo, Q. feng; Ma, C.; Zhang, J.; Zhao, R.; Yang, T.; Du, Y.; Chen, X.; Li, T.; Liu, X.; Song, X.; Yang, Y.; Yang, Z.; Liu, S.; Zhang, Y.; Zhao, K. Wearable Photoferroelectric Perovskite X-Ray Detectors. *Adv. Funct. Mater.* **2024**, *34* (46), 2407693. https://doi.org/10.1002/adfm.202407693.

(56) Demchyshyn, S.; Verdi, M.; Basiricò, L.; Ciavatti, A.; Hailegnaw, B.; Cavalcoli, D.; Scharber, M. C.; Sariciftci, N. S.; Kaltenbrunner, M.; Fraboni, B. Designing Ultraflexible Perovskite X-Ray Detectors through Interface Engineering. *Advanced Science* **2020**, *7* (24), 2002586. https://doi.org/10.1002/advs.202002586.

(57) Li, H.; Liu, X.; Yang, T.; Ma, C.; Du, Y.; Xu, P.; Zhang, L.; Song, X.; Cui, Q.; Zhao, S.; Yang, Z.; Liu, S. F.; Jin, S.; Zhao, K. Flexible Large-Scale Self-Driven Perovskite X-Ray Detector by Precise Heterogeneous Integration. *ACS Energy Lett.* **2024**, *9* (1), 64–74. https://doi.org/10.1021/acsenergylett.3c02152.

(58) Li, W.; Xu, Y.; Peng, J.; Li, R.; Song, J.; Huang, H.; Cui, L.; Lin, Q. Evaporated Perovskite Thick Junctions for X-Ray Detection. *ACS Appl. Mater. Interfaces* **2021**, *13* (2), 2971–2978. https://doi.org/10.1021/acsami.0c20973.

(59) Basiricò, L.; Senanayak, S. P.; Ciavatti, A.; Abdi-Jalebi, M.; Fraboni, B.; Sirringhaus, H. Detection of X-Rays by Solution-Processed Cesium-Containing Mixed Triple Cation Perovskite Thin Films. *Adv. Funct. Mater.* **2019**, *29* (34), 1902346. https://doi.org/10.1002/adfm.201902346.

(60) Tao, K.; Xiong, C.; Lin, J.; Ma, D.; Lin, S.; Wang, B.; Li, H. Self-Powered Photodetector Based on Perovskite/NiOx Heterostructure for Sensitive Visible Light and X-Ray Detection. *Adv. Electron. Mater.* **2023**, *9* (3), 2201222. https://doi.org/10.1002/aelm.202201222.

(61) Wu, J.; Wang, L.; Feng, A.; Yang, S.; Li, N.; Jiang, X.; Liu, N.; Xie, S.; Guo, X.; Fang, Y.; Chen, Z.; Yang, D.; Tao, X. Self-Powered FA0.55MA0.45PbI3 Single-Crystal

Perovskite X-Ray Detectors with High Sensitivity. *Adv. Funct. Mater.* **2022**, *32* (9), 2109149. https://doi.org/10.1002/adfm.202109149.

(62) Zhao, H.; Qiu, J.; Hou, B.; Yi, L.; Qin, X.; Liu, X. Flexible Perovskite X-Ray Detectors through Interfacial Modification with Perylene Diimide. *Adv. Opt. Mater.* **2023**, *11* (7), 2202668. https://doi.org/10.1002/adom.202202668.

(63) Landi, N.; Maurina, E.; Marongiu, D.; Simbula, A.; Borsacchi, S.; Calucci, L.; Saba, M.; Carignani, E.; Geppi, M. Solid-State Nuclear Magnetic Resonance of Triple-Cation Mixed-Halide Perovskites. *Journal of Physical Chemistry Letters* **2022**, *13* (40), 9517–9525. https://doi.org/10.1021/acs.jpclett.2c02313.

(64) He, Y.; Hadar, I.; Kanatzidis, M. G. Detecting Ionizing Radiation Using Halide Perovskite Semiconductors Processed through Solution and Alternative Methods. *Nat. Photonics* **2022**, *16* (1), 14–26. https://doi.org/10.1038/s41566-021-00909-5.

(65) Dudipala, K. R.; Le, T. H.; Nie, W.; Hoye, R. L. Z. Halide Perovskites and Their Derivatives for Efficient, High-Resolution Direct Radiation Detection: Design Strategies and Applications. *Advanced Materials* **2024**, *36* (8), 2304523. https://doi.org/10.1002/adma.202304523.

(66) Klein, C. A. Bandgap Dependence and Related Features of Radiation Ionization Energies in Semiconductors. *J. Appl. Phys.* **1968**, *39* (4), 2029–2038. https://doi.org/10.1063/1.1656484.
